# Gate-tunable giant negative magnetoresistance in tellurene driven by quantum geometry

**DOI:** 10.1038/s41467-026-74066-0

**Published:** 2026-06-05

**Authors:** Marcello B. Silva Neto, Chang Niu, Marcus V. O. Moutinho, Pierpaolo Fontana, Claudio Iacovelli, Victor Velasco, Caio Lewenkopf, Peide D. Ye

**Affiliations:** 1 Instituto de Física, Universidade Federal do Rio de Janeiro, Rio de Janeiro, RJ Brazil; 2 Elmore Family School of Electrical and Computer Engineering, Purdue University, West Lafayette, Indiana USA; 3 Birck Nanotechnology Center, Purdue University, West Lafayette, Indiana USA; 4 Universidade Federal do Rio de Janeiro - Campus Duque de Caxias, Duque de Caxias, RJ Brazil; 5 Departament de Física, Universitat Autònoma de Barcelona, Bellaterra, Spain; 6 Independent Researcher, Barcelona, Spain; 7 International School for Advanced Studies (SISSA), Trieste, Italy

**Keywords:** Topological matter, Two-dimensional materials, Two-dimensional materials

## Abstract

Negative magnetoresistance in conventional two-dimensional electron gases is a well known phenomenon, but its origin in complex and topological materials endowed with nontrivial quantum geometry remains elusive. Here, we report a giant negative magnetoresistance reaching  −90% of the zero-field resistance, *R*_0_, in *n*-type tellurene films. The effect persists up to 35 T at cryogenic temperatures and is suppressed when the chemical potential moves away from the conduction-band Weyl node, suggesting a quantum geometric origin. We propose two mechanisms: quantum geometric enhancement of diffusion and a magnetoelectric spin interaction that locks the spin of a cyclotron-moving Weyl fermion, in the presence of an intrinsic inversion-breaking polar field ${{\boldsymbol{{\mathcal{E}}}}}$ and an applied magnetic field **B**, to its guiding-center drift, $({{\boldsymbol{{\mathcal{E}}}}}\times {{{\bf{B}}}})\cdot \sigma$. The resulting diffusion enhancement yields $\Delta {R}_{zz}/{R}_{0}=-{\beta }_{g}{({{\boldsymbol{{\mathcal{E}}}}}\times {{{\bf{B}}}})}^{2}$, with *β*_*g*_ set by the quantum metric. Our findings establish a quantum geometric, non-Markovian memory effect in magnetotransport.

## Abstract (web summary)

This study reports gate-tunable giant negative magnetoresistance exceeding  − 90% in n-type tellurene thin films, stable up to 35 T at low temperatures and suppressed when the chemical potential moves off the conduction-band Weyl node.

## Introduction

The discoveries of giant magnetoresistance (GMR) in metallic multilayers^1,2^ and of colossal magnetoresistance (CMR) in correlated oxides^3^ illustrate how intricate electron interactions produce stark resistance changes. In bulk materials, these effects arise from spin-polarized scattering or orbital coupling^4^, while in thin films, reduced dimensionality unlocks magnetoresistance via quantum confinement^5^ or topological protection^6^. The advent of two-dimensional (2D) materials has particularly transformed the landscape, offering atomic-scale thickness and electrostatic gate-control that reveal novel magnetoresistive behaviors inaccessible in conventional systems.

Rare in bulk systems, negative magnetoresistance (NMR) has been observed in various 2D materials with distinct magnitudes, field ranges, and underlying mechanisms. In graphene, for example, weak NMR of −2% at 1 mT arises from weak localization (WL) due to quantum interference of backscattered electron waves^7^. Monolayer MoS_2_ exhibits stronger NMR of −10% at 5 T, attributed to intervalley scattering mediated by spin-orbit coupling^8^, while transition metal dichalcogenide heterostructures (e.g., WS_2_/WSe_2_) show NMR of −12% at 3 T, from moiré-induced Berry curvature^9^. Beyond these, black phosphorus displays NMR of −15% at 2 T due to anisotropic scattering in its puckered lattice^10^, and topological insulators, like Bi_2_Se_3_ thin films, show NMR up to −20% at 1−2 T^11^ linked to helical surface states protected by time-reversal symmetry. Meanwhile, Cr_2_S_3_, a 2D ferromagnet, demonstrates NMR of −25% at 100 K due to spin-filtering effects at magnetic domain walls^12^, and twisted bilayer graphene near magic angles exhibits NMR of −30% at 1 T. Most remarkably, in topological Dirac semimetals such as individual Cd_3_As_2_ nanowires, giant NMR reaches −63% at 60 K, attributed to the chiral anomaly ^13^.

A non-saturating magnetoresistance signals a departure from simple Markovian transport^14^, a memory of the carrier’s past history, encoded in its momentum or phase, influencing its future trajectory. For example, in systems with dilute, strong scatterers (e.g., hard disks or antidots of radius *a*), and at low fields (*ω*_*c*_*τ* ≪ 1, where *ω*_*c*_ is the cyclotron frequency and *τ* is the relaxation time), the dominant memory effect is purely classical and geometric: backscattering events induce a NMR, $\Delta {R}_{xx}/{R}_{0} \sim -{({\omega }_{c}\tau )}^{2}/{\beta }_{0}$, where *β*_0_ = *a*/*ℓ*, due to correlated returns to scatterers of mean-free-path *ℓ*^15^, as described by the Lorentz gas model^16^. For 2DEGs with smooth disorder (e.g., remote impurities), guiding center diffusion leads to $\Delta {R}_{xx}/{R}_{0} \sim -{({\omega }_{c}/{\omega }_{0})}^{2}$ at low fields (*ω*_*c*_ ≪ *ω*_0_), where ${\omega }_{0} \sim {v}_{F}{({a}^{2}{\ell }_{S}{\ell }_{L})}^{-1/4}$^17,18^, with *ℓ*_*S*_ and *ℓ*_*L*_ representing the correlation lengths of the small and large disorder potentials^17,19^. Beyond these classical pictures, NMR can also emerge from the quantum mechanical memory of the electron’s phase in disordered systems, from WL and electron-electron interactions. At low fields ($B\lesssim \hslash /e{\ell }_{\phi }^{2}$, where *ℓ*_*ϕ*_ is the phase coherence length), WL induces NMR, *ΔR*_*xx*_/*R*_0_ ~ − 1%, through constructive interference of time-reversed paths^20^, becoming irrelevant in the classical regime (*ℏω*_*c*_ ≪ *k*_*B*_*T*) or under strong inelastic scattering^21^. In contrast, interactions drive NMR via diffusive (*k*_*B*_*Tτ*/*ℏ* ≪ 1) or ballistic (*k*_*B*_*Tτ*/*ℏ* ≫ 1) corrections, producing a parabolic field dependence, *ΔR*_*xx*_/*R*_0_ ~ −*B*^2^, with larger magnitudes (~ 1−10%) observed in clean, high-density GaAs heterostructures^22,23^.

Here we report the observation of gate-controlled, giant negative magnetoresistance (GNMR) in *n*-type tellurium (Te) flakes, originating from its intrinsic (Weyl) quantum geometry and from the inversion-breaking polar strength associated with the lone-pair texture, conveniently parameterized at low energies by an effective field ${{\boldsymbol{{\mathcal{E}}}}}$^24^. Our GNMR reaches up to −90% at 32 T, far exceeding the field range and magnitude of prior 2D systems. We attribute this GNMR to the quantum geometric enhancement of diffusion, a novel mechanism distinct from chiral anomaly^25,26^, or charge-to-spin conversion^27^, or disorder-driven models^17^, in which the quantum metric promotes velocity fluctuations between the spin-split bands, enhancing diffusion and reducing the resistance. The strength of the effect, −*β*_***g***_(*Δϵ*)^2^, is determined by the quantum metric, ***g***, encoded in the parameter *β*_***g***_, and by the energy splitting, ${(\Delta \epsilon )}^{2} \sim {({{\boldsymbol{{\mathcal{E}}}}}\times {{{\bf{B}}}})}^{2}$, produced by ${H}_{DZ}=-\gamma ({{\boldsymbol{{\mathcal{E}}}}}\times {{{\bf{B}}}})\cdot {{{\boldsymbol{\sigma }}}}$, that we term the Drift-Zeeman coupling, a novel magnetoelectric-spin interaction that locks the spin of carriers crossing a region of ${{\boldsymbol{{\mathcal{E}}}}}\perp {{{\bf{B}}}}$ fields to its drift-momentum, with *γ* determined by the anisotropic spin-orbit interaction. Our work, therefore, establishes a direct manifestation of the non-Markovian memory principle in Te through a previously unexplored quantum mechanical effect: the memory of the wavefunction’s quantum geometric structure across the Brillouin zone.

## Results

### Gate-tunable quantum transport in tellurene

**Fig. 1:**
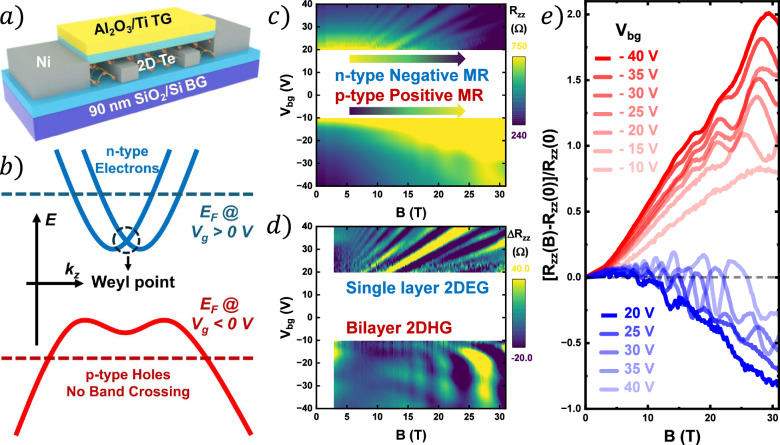
Device schematics and gate-tunable quantum transport in *p*- and *n*-type Te. **a** Schematic illustration of a dual-gated Hall-bar device based on a Te flake for transport measurements, showing the *Al*_2_*O*_3_ top gate (TG) and the *SiO*_2_/*Si* back gate (BG). **b** Band structure of Te. At zero gate bias, Te is nearly intrinsic. Applying a positive gate voltage populates the conduction band, where the spin-split subbands cross at the H point to form a Weyl node. In contrast, a negative gate voltage accesses the valence band without band crossing, enabling comparison between distinct quantum transport regimes governed by quantum geometry. **c** Color map of magnetoresistance (*R*_*zz*_) as a function of back-gate voltage and magnetic field, showing negative MR for electron conduction and positive MR for hole conduction within the same device. **d** Background-subtracted *ΔR*_*zz*_ revealing clear SdH oscillations in both *n*-type and *p*-type regimes. In the *n*-type regime, the SdH oscillations exhibit a single characteristic frequency, consistent with a single-layer 2DEG. In the *p*-type regime, two characteristic frequencies are observed, consistent with a bilayer 2DHG. **e** Overlay of normalized magnetoresistance curves for *p*-type and *n*-type conduction, highlighting their contrasting field responses.

We experimentally investigate the magnetotransport properties of Te using Hall-bar devices fabricated on a silicon dioxide substrate, with a highly doped silicon back gate and an *Al*_2_*O*_3_ top gate, as shown in Fig. 1a. Owing to the narrow bandgap of Te^28^, both carrier type and carrier density can be continuously tuned within a single device through electrostatic gating. Figure 1b presents the band structure of Te, where the conduction band splits and crosses at the H point to form a Weyl node, imparting nontrivial quantum geometry to the charge carriers. In contrast, the valence band shows no such crossing. This distinct band topology provides an exceptional platform for exploring quantum-geometry-driven transport phenomena in Te. Figure 1c presents a color map of the longitudinal magnetoresistance (*R*_*zz*_) as a function of gate voltage (carrier density) and magnetic field. Distinct magnetoresistive behaviors are observed for electrons and holes: the *n*-type regime exhibits NMR, whereas the *p*-type regime shows positive magnetoresistance (PMR). After subtracting the background resistance, Shubnikov-de Haas (SdH) oscillations emerge in both conduction and valence bands, as shown in Fig. 1d. The corresponding Landau fan diagram reveals two distinct oscillation frequencies in the *p*-type regime, originating from the dual-surface (top and bottom) accumulation layers inherent to Te and consistent with bilayer transport behavior (bilayer 2DHG)^29^. In contrast, the *n*-type regime displays a single oscillation series, consistent with depletion of the top surface and single-layer transport behavior (single-layer 2DEG). Figure 1e shows the normalized magnetoresistance measured across different carrier types and densities, revealing a clear transition from PMR to NMR at a cryogenic temperature of 350 mK. This gate-tunable NMR persists over a wide range of perpendicular magnetic fields, up to 35 T. As the carrier density of the 2DEG increases, shifting the chemical potential away from the Weyl node near the conduction band edge, the magnitude of the NMR gradually diminishes. In contrast, only conventional PMR is observed in the 2DHG under negative back-gate bias (Fig. 1f), consistent with the expected two-surface hole accumulation^29^.

**Fig. 2:**
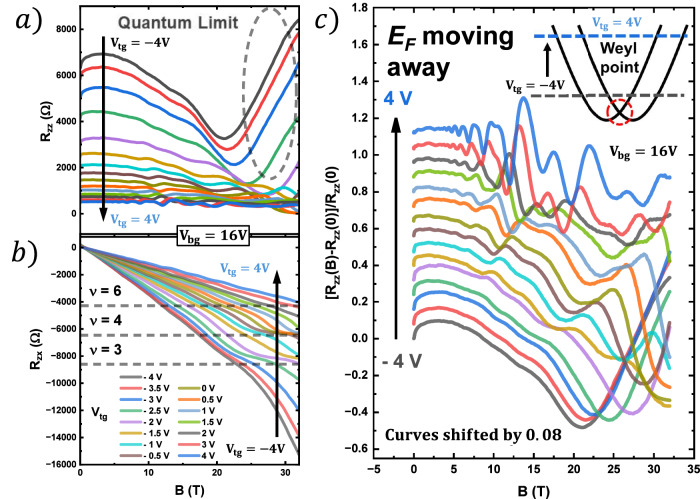
Carrier-density dependence of negative magnetoresistance and the quantum Hall effect. **a** Longitudinal magnetoresistance (*R*_*zz*_) as a function of carrier density, showing a pronounced negative magnetoresistance (NMR) that persists until the system reaches the quantum limit at the lowest Landau level (*n* = 0). **b** Transverse Hall resistance (*R*_*zx*_) versus carrier density, revealing well-developed quantum Hall plateaus at filling factors *ν* = 3, 4, and 6. **c** Normalized magnetoresistance in the Te conduction band as a function of carrier density. The NMR gradually weakens with increasing gate voltage as the Fermi level shifts away from the Weyl node. Inset: schematic illustration of the carrier-density-dependent Fermi-level movement relative to the Weyl node, as the top-gate voltage is swept from −4 V to 4 V in the conduction band.

Figure 2a, b presents the carrier density dependence of the longitudinal and transverse magnetoresistance in the Te conduction band, respectively. Low carrier densities were accessed by fixing the back-gate voltage at *V*_*bg*_ = 16 V, ensuring good contact properties, while tuning the top-gate voltage to modulate the carrier density between 2 × 10^12^ cm^−2^ to 1 × 10^13^ cm^−2^^30^. Well-developed quantum Hall plateaus of filling factor *ν* = 3, 4, and 6 are observed. NMR emerges before the system reaches the quantum limit (Landau level *n* = 0), occurring at high magnetic fields and low carrier densities. Upon increasing the applied magnetic field, the resistance initially decreases to a minimum at a critical crossover field before transitioning to PMR. In the quantum limit, the NMR is suppressed as the electrons become localized by cyclotron motion, eliminating interband contributions from the quantum geometric mechanism (see Section 3). Figure 2c shows the carrier density dependence of the normalized magnetoresistance, illustrating how the NMR evolves with the Fermi level position relative to the Weyl node in the conduction band. Our analysis focuses on the magnetic field range between 2 T and 25 T to 35 T, where the NMR dominates. Below 2 T, the transport is instead governed by weak antilocalization, a signature of strong spin-orbit coupling in Te^31^. Notably, the crossover field between NMR and PMR shifts to higher magnetic fields with increasing carrier density. All curves are quantitatively described by a parabolic dependence, *ΔR*_*zz*_(*B*)/*R*_0_ ∝ −*B*^2^, where the proportionality factor varies with the applied voltage *V*, the orientation of the magnetic field *θ*, and the temperature *T*. This parabolic NMR represents a low-field expansion valid for *B* ≪ B^*^, where B^*^ is a yet-to-be-determined saturation field characteristic of the quantum-geometric mechanism.

### Field rotation and temperature studies

**Fig. 3:**
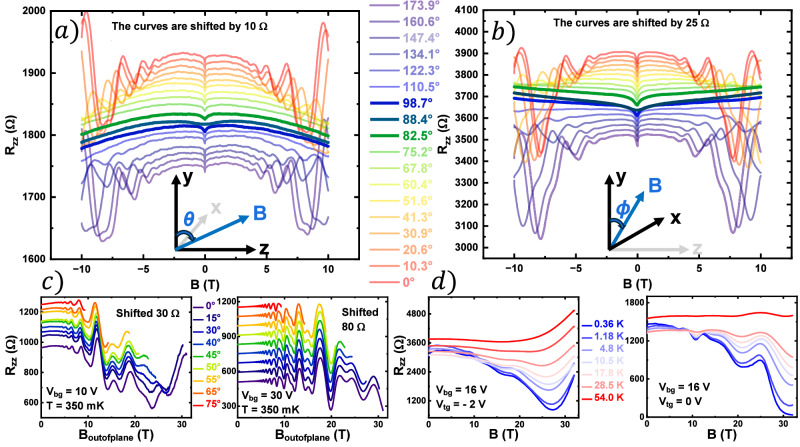
Angular, carrier-density, and temperature dependence of the NMR. **a** Magnetic-field rotation within the *yz* plane, perpendicular to the device in-plane *x* axis. The angle *θ* is measured from the film normal ($\widehat{y}$). The giant NMR weakens but persists as the field approaches the in-plane $\widehat{z}$ direction (*θ* = 90^∘^). **b** Magnetic-field rotation within the *xy* plane, perpendicular to the device in-plane *z* axis. The angle *ϕ* is measured from the film normal ($\widehat{y}$). The giant NMR vanishes completely when the field is aligned along the in-plane $\widehat{x}$ direction (*ϕ* = 90^∘^), which is parallel to the intrinsic polarization field ${{\boldsymbol{{\mathcal{E}}}}}$. **c** Evolution of the SdH oscillations for tilted magnetic fields (0 ≤ *θ *≤ 75^∘^). No significant change in the SdH sequences is observed at low (*V*_bg_ = 10 V) and high (*V*_bg_ = 30 V) carrier densities, indicating an ultra-small *g* factor in the Te conduction band. **d** Temperature dependence of the NMR at different carrier densities. The effect is strongly suppressed with increasing temperature, vanishing entirely at approximately 54 K.

To further elucidate the nature of the observed NMR, we performed a series of field rotation experiments on *n*-type Te devices. In Fig. 3a, the magnetic field is rotated within the *yz* plane, where the *z*-axis is aligned with the chiral direction of the Te atomic helices and the current flow, and the *y*-axis is perpendicular to the 2D Te flake. The data reveal two critical insights: while the SdH oscillations persist only when the field has a component perpendicular to the film, the NMR remains present for all field orientations, albeit with varying strength. Specifically, the NMR is strongest when the magnetic field is perpendicular to the film, characterized by a tighter parabolic drop in resistance. As the field is rotated and becomes aligned with the *z*-axis (parallel to the current), the NMR weakens, following a looser parabolic trend. As we demonstrate in Sec. 3, this behavior originates from the anisotropic spin-orbit interaction in Te. We note that the small antisymmetric component observed in Fig. 3a can be attributed to extrinsic effects, such as Hall leakage into the longitudinal channel caused by slight sample misalignment (see Supplementary Information).

Figure 3b presents data from a rotation of the magnetic field within the *xy*-plane. Here, the *x*-axis corresponds to the in-plane direction perpendicular to the current and, crucially, it is aligned to the large macroscopic electric polarization, ${{\boldsymbol{{\mathcal{E}}}}}$, arising from the lone pairs^24^. Notably, the NMR vanishes entirely when ${{{\bf{B}}}}\parallel {{\boldsymbol{{\mathcal{E}}}}}$. The residual small PMR observed in this configuration is due to an extrinsic orbital mechanism of the Parish-Littlewood type, stemming from charge inhomogeneities^32^ as explained in the Supplementary Information. Taken together, the results from these two rotation planes provide compelling evidence that the low-field, parabolic magnetoresistance term is proportional to the square of the cross product between ${{\boldsymbol{{\mathcal{E}}}}}$ and **B**, or $\Delta {R}_{zz}/{R}_{0}=-{\beta }_{g}{({{\boldsymbol{{\mathcal{E}}}}}\times {{{\bf{B}}}})}^{2}$. We provide a formal derivation of this geometric dependence in Sec. 3.

Figure 3c shows the evolution of the magnetoresistance as a function of the out-of-plane magnetic field for tilted-field configurations in an *n*-type Te device, measured at two representative carrier densities (*V*_bg_ = 10 V and 30 V). The data confirm the robustness of the NMR under tilted magnetic fields and highlight its clear dependence on carrier density, with a stronger effect observed at lower carrier densities, corresponding to Fermi levels closer to the Weyl node. No significant change is found in the angle-dependent SdH oscillation sequences at low (*V*_bg_ = 10 V) and high (*V*_bg_ = 30 V) carrier densities, suggesting an ultra-small *g*-factor in the Te conduction band^30^ and confirming the absence of ordinary Zeeman splitting under tilted magnetic fields. Furthermore, the observed SdH frequencies indicate a single occupied confinement subband and a 2D Fermi surface. We incorporate this quasi-2D character explicitly into our theoretical analysis. Finally, Fig. 3d further illustrates the temperature dependence of the NMR. The effect is strongly suppressed with increasing temperature, completely vanishing around 54 K. This behavior is consistently observed in different carrier densities (and devices), and confirms the quantum geometric origin of the GNMR, as the geometric interband contribution (dominant at low T) is overshadowed by the thermal intraband one.

## Discussion

### Geometric diffusion

The observation of a gate-tunable, parabolic GNMR in Te exhibiting a distinct $\Delta {R}_{zz}/{R}_{0}=-{\beta }_{g}{({{\boldsymbol{{\mathcal{E}}}}}\times {{{\bf{B}}}})}^{2}$ dependence defies explanation by the mechanisms discussed in Sec. 1. We propose that this phenomenon arises from a quantum geometric enhancement of carrier diffusion, combined with a previously unreported magnetoelectric spin interaction. Our starting point is the Kubo-Greenwood formula^33^ for the conductivity tensor, *σ*_*ij*_, which for an *n* band system in *d* dimensions is given by

1 $${\sigma }_{ij}={e}^{2}{\sum}_{n}\int \frac{{d}^{d}{{{\bf{k}}}}}{{(2\pi )}^{d}}{D}_{ij}^{(n)}({{{\bf{k}}}})\left(-\frac{\partial f({\epsilon }_{n}({{{\bf{k}}}}))}{\partial {\epsilon }_{n}({{{\bf{k}}}})}\right),$$

where *e* is the electric charge, *ϵ*_*n*_(**k**) is the *n*−th band dispersion relation, *f*(*ϵ*_*n*_(**k**)) is the equilibrium Fermi-Dirac distribution, and ${D}_{ij}^{(n)}({{{\bf{k}}}})$ is the diffusion tensor, defined as the time integral of the velocity auto-correlation^34^ between Bloch states $\left|{u}_{n}({{{\bf{k}}}})\right\rangle$

2 $${D}_{ij}^{(n)}({{{\bf{k}}}})={\int }_{0}^{\infty }dt\,\left\langle {u}_{n}({{{\bf{k}}}})\right|{v}_{i}(t){v}_{j}(0)\left|{u}_{n}({{{\bf{k}}}})\right\rangle .$$

In a multi-band system, like the conduction band of Te, the velocity operator comprises a conventional intraband group velocity and an interband component responsible for quantum geometric fluctuations, ${v}_{i}(t)={\partial }_{{k}_{i}}\epsilon ({{{\bf{k}}}})/\hslash+\delta {v}_{i}(t)$. Evaluating the correlation function $\langle \delta {v}_{i}(t)\delta {v}_{j}(0)\rangle \sim \langle \delta {v}_{i}\delta {v}_{j}\rangle {e}^{-{\Gamma }_{g}t}$, where *τ*_*g*_ = 1/*Γ*_*g*_ is a temperature independent quantum geometric relaxation time, yields the central result for the diffusion tensor ${D}_{ij}(\mu )={\sum }_{n=\pm }{D}_{ij}^{(n)}(\mu )$, where *n* = ± denotes the conduction bands, at the chemical potential *μ*

3 $${D}_{ij}(\mu )=\underbrace{{D}_{ij}^{D}(\mu )}_{{{\rm{intraband}}}}+\underbrace{\frac{2{\tau }_{g}}{{\hslash }^{2}}\langle {[\Delta \epsilon ({{{\bf{k}}}})]}^{2}{g}_{ij}({{{\bf{k}}}}){\rangle }_{{{{\rm{F}}}}{{{\rm{S}}}}}}_{{{{\rm{interband}}}} ({{{\rm{geometric}}}})}.$$

Here, ${D}_{ij}^{D}(\mu )$ is the Drude (intraband) Markovian contribution to the diffusion tensor^33^ and ${\langle {[\Delta \epsilon ({{{\bf{k}}}})]}^{2}{g}_{ij}({{{\bf{k}}}})\rangle }_{{{{\rm{FS}}}}}$ is the Fermi-surface average of the band-splitting squared times the quantum metric ${g}_{ij}({{{\bf{k}}}})=\,{{{\rm{Re}}}}\,[\langle {\partial }_{{k}_{i}}u| {\partial }_{{k}_{j}}u\rangle -\langle {\partial }_{{k}_{i}}u| u\rangle \langle u| {\partial }_{{k}_{j}}u\rangle ]$, that quantifies the overlap between Bloch states in **k** − space^35,36^. This geometric contribution that arises from the time-integrated velocity auto-correlation in Eq. (2) is a **k**-space effect that is fundamentally non-Markovian, a memory of the carrier’s past history encoded in its quantum geometric structure. It influences the carrier’s trajectory by enhancing diffusion and reducing the resistance, while the field dependence of the band splitting will ultimately describe the experimental GNMR. The novel mechanism reported here is the transport analog of the quantum geometric enhancement of the superfluid weight in conventional^37^ and flat-band superconductors^38,39^.

### The drift–Zeeman interaction

**Fig. 4:**
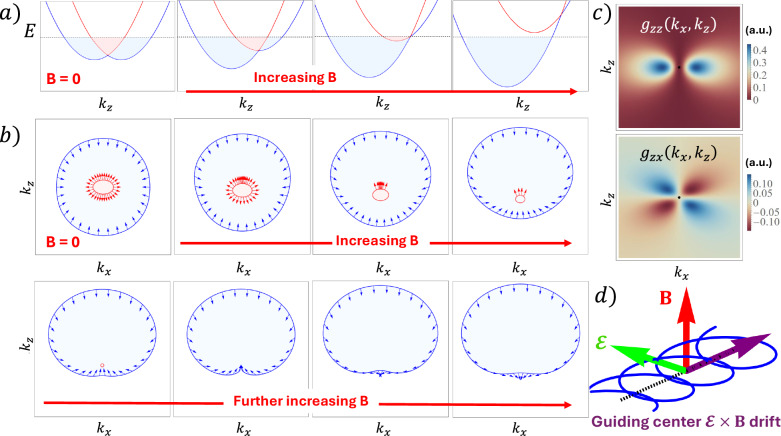
Quantum geometry and spin-drift locking in Te. **a** Conduction bands *ϵ*_±_(*k*_*z*_) of Te at a given chemical potential, featuring a Weyl node located at *k*_*z*_ = 0, and their evolution with magnetic field, ${{{\boldsymbol{B}}}}\parallel \widehat{{{{\boldsymbol{y}}}}}$. **b** Evolution of the radial spin texture, with blue and red arrows representing the outer and inner Fermi surfaces, respectively, as the magnetic field is increased. **c** Quantum metric components *g*_*zz*_(***k***) and *g*_*zx*_(***k***). **d** Locking of the spin to the guiding-center drift upon crossed electric ${{\boldsymbol{{\mathcal{E}}}}}$ and magnetic ***B*** fields.

The geometrically enhanced diffusion given by Eq. (3) is activated by a magnetic-field-dependent energy splitting, *Δϵ*(**k**), between the two non-degenerate bands (Fig. 4a). The ultra-small effective *g*-factor in the conduction band of Te^30^ indicates that an ordinary Zeeman effect is negligible, implying that a different mechanism must be responsible for the observed band splitting.

To establish the microscopic origin of this splitting, we derive a low-energy theory for tellurium. We start from a multiband **k**⋅**p** Hamiltonian with an inversion-odd crystal potential and perform a systematic Löwdin (Schrieffer–Wolff) downfolding onto the conduction doublet (see Supplementary Information). In this procedure, the inversion-odd part of the periodic crystal potential generates, at zero magnetic field, the anisotropic Weyl-like spin texture of tellurium, while minimal coupling **p** → **p** + *e***A** yields the leading field-induced magnetoelectric corrections.

To first order in both the polar strength and the magnetic field, the projected bulk Hamiltonian takes the form

4 $${H}_{{{{\rm{eff}}}}}^{{{{\rm{(3D)}}}}}({{{\bf{k}}}})=	{\varepsilon }_{0}({{{\bf{k}}}})\,{{\mathbb{I}}}_{2}+{\lambda }_{\perp }({k}_{x}{\sigma }_{x}+{k}_{y}{\sigma }_{y})+{\lambda }_{z}{k}_{z}{\sigma }_{z}-{{{\boldsymbol{\gamma }}}}\cdot ({{\boldsymbol{{\mathcal{E}}}}}\times {{{\bf{B}}}})\cdot {{{\boldsymbol{\sigma }}}} \\ 	+\kappa ({{\boldsymbol{{\mathcal{E}}}}}\times {{{\bf{B}}}})\cdot {{{\bf{k}}}}\,{{\mathbb{I}}}_{2}+\cdots \,,$$

where *ε*_0_(**k**) is the spin-independent dispersion, *λ*_*z*_ > *λ*_⊥_ are the anisotropic Weyl couplings, the fourth term is the spin-dependent Drift–Zeeman interaction with strength *γ*, and the fifth term is the scalar Drift–Orbit interaction with strength *κ*. Here, ${{\boldsymbol{{\mathcal{E}}}}}\approx ({{{{\mathcal{E}}}}}_{x},0,0)$ serves as a compact low-energy parameterization of the inversion-breaking polar strength associated with the lone-pair polarization^24^. Additional higher-order or subleading projected terms are not shown and are discussed in the Supplementary Information.

The Drift–Zeeman interaction is identified as

5 $${H}_{DZ}=-\,{{{\boldsymbol{\gamma }}}}\cdot ({{\boldsymbol{{\mathcal{E}}}}}\times {{{\bf{B}}}})\cdot {{{\boldsymbol{\sigma }}}},$$

where ***γ*** = (*γ*_⊥_, *γ*_⊥_, *γ*_*z*_) accounts for the anisotropy of the projected magnetoelectric spin coupling. This term is governed by the same spin-orbit-coupled conduction doublet that generates the anisotropic Weyl terms, resulting in *γ*_*z*_ > *γ*_⊥_ (see Supplementary Information). Higher-order gauge-invariant magnetoelectric spin interactions, such as ${B}^{2}({{\boldsymbol{{\mathcal{E}}}}}\times {{{\bf{B}}}})\cdot {{{\boldsymbol{\sigma }}}}$ or $({{\boldsymbol{{\mathcal{E}}}}}\cdot {{\boldsymbol{{\mathcal{E}}}}})({{\boldsymbol{{\mathcal{E}}}}}\times {{{\bf{B}}}})\cdot {{{\boldsymbol{\sigma }}}}$, are allowed by symmetry but are subleading in our low-energy expansion.

### The negative magnetoresistance

The Drift-Zeeman interaction (5) profoundly alters the electronic structure of Te. For ${{{\bf{B}}}}\parallel \widehat{y}$ and ${{\boldsymbol{{\mathcal{E}}}}}\parallel \widehat{x}$^24^, it shifts the Weyl node along the *k*_*z*_-axis (Fig. 4a), thereby modifying the Fermi surface topology and spin texture (Fig. 4b)^40^. Consequently, states from the inner Fermi surface are transferred to the outer Fermi surface, while the overall spin texture reorients itself to align with the effective ${{\boldsymbol{{\mathcal{E}}}}}\times {{{\bf{B}}}}$ field. Because the transport regime is quasi-2D, we introduce a confining potential *V*_conf_(*y*) that restricts motion to a slab of thickness *L* along $\widehat{y}$, the direction. We factorize the 3D wavefunction as *Ψ*(**r**) = *ψ*(*x*, *z*) *ϕ*_1_(*y*), where *ϕ*_1_(*y*) is the lowest confinement subband envelope and *ψ*(*x*, *z*) is the effective in-plane spinor wavefunction. The reduced, quasi-2D Hamiltonian is obtained by projecting the bulk parent Hamiltonian onto the lowest subband, ${H}_{{{{\rm{eff}}}}}^{{{{\rm{(2D)}}}}}=\left\langle {\phi }_{1}\right|{H}_{{{{\rm{eff}}}}}^{{{{\rm{(3D)}}}}}{\left|{\phi }_{1}\right\rangle }_{y}$. Since linear terms in *k*_*y*_ vanish upon subband projection and quadratic ${k}_{y}^{2}$ terms produce only a constant confinement shift, the effective low-energy, quasi-2D Hamiltonian is obtained by setting *k*_*y*_ → 0 in (4). Consequently, the quantum geometric contribution to the diffusive transport (3) only on (*k*_*x*_, *k*_*z*_) is

6 $$\begin{array}{l}{\langle {[\Delta \epsilon ({{{\bf{k}}}})]}^{2}{g}_{zz}({{{\bf{k}}}},{{{\bf{B}}}})\rangle }_{{{{\rm{FS}}}}}=4{\langle ({\lambda }_{\perp }^{2}{k}_{x}^{2}+{\lambda }_{z}^{2}{k}_{z}^{2}){g}_{zz}({{{\bf{k}}}},{{{\bf{B}}}})\rangle }_{{{{\rm{FS}}}}}\\ -8{\gamma }_{z}{\langle {k}_{z}{g}_{zz}({{{\bf{k}}}},{{{\bf{B}}}})\rangle }_{{{{\rm{FS}}}}}\,\widehat{z}\cdot ({{\boldsymbol{{\mathcal{E}}}}}\times {{{\bf{B}}}})+4{\langle {g}_{zz}({{{\bf{k}}}},{{{\bf{B}}}}){[\gamma \cdot ({{\boldsymbol{{\mathcal{E}}}}}\times {{{\bf{B}}}})]}^{2}\rangle }_{{{{\rm{FS}}}}},\end{array}$$

where the field dependent quantum metric is given by

7 $${g}_{zz}({{{\bf{k}}}},{{{\bf{B}}}})=\frac{{\lambda }_{z}^{2}{\lambda }_{\perp }^{2}{k}_{x}^{2}}{4{\left[{k}_{x}^{2}{\lambda }_{\perp }^{2}+{({k}_{z}{\lambda }_{z}+{\gamma }_{z}{{{{\mathcal{E}}}}}_{x}{B}_{y})}^{2}+{\gamma }_{\perp }^{2}{{{{\mathcal{E}}}}}_{x}^{2}{B}_{z}^{2}\right]}^{2}}.$$

Here, 〈⋯ 〉_FS_ denotes a 2D Fermi-surface average over the confined *k*_*x*_–*k*_*z*_ subspace, consistent with the single-subband quasi-2D transport regime revealed by the SdH oscillations. Expansion of ${\langle {k}_{z}{g}_{zz}({{{\bf{k}}}},{{{\bf{B}}}})\rangle }_{{{{\rm{FS}}}}}\widehat{z}\cdot ({{\boldsymbol{{\mathcal{E}}}}}\times {{{\bf{B}}}})$, in powers of the magnetic field, yields only nonzero even powers of *B*. Similarly, expansion of ${\langle {g}_{zz}({{{\bf{k}}}},{{{\bf{B}}}})\rangle }_{{{\mathrm{FS}}}}{({{\boldsymbol{{\mathcal{E}}}}}\times {{{\bf{B}}}})}^{2}$ also generates only nonzero even powers of *B*. Furthermore, for fields smaller than ${B}^{*} \sim {\lambda }_{z}{k}_{F}/{\gamma }_{z}{{{{\mathcal{E}}}}}_{x} \sim 40$T, the *B*^4^ correction to the geometric diffusion is negligible, and the symmetric contribution becomes $\Delta {R}_{zz}(B)/{R}_{0}=-{\beta }_{g}{({{\boldsymbol{{\mathcal{E}}}}}\times {{{\bf{B}}}})}^{2}$, as observed experimentally, with ${\beta }_{g}\propto {\langle {g}_{zz}({{{\bf{k}}}})\rangle }_{{{{\rm{FS}}}}}$ given in terms of the zero field quantum metric. Since the typical energy scales provided by an external magnetic field are usually quite weak, the Weyl node remains very close to the high-symmetry *H* point, corresponding to the first panel in Fig. 4a, and the GNMR is therefore attributed almost entirely to the strongly diverging, zero-field quantum metric near the Weyl node. In addition, no Hall contribution from ${\langle {g}_{zx}({{{\bf{k}}}})\rangle }_{{{{\rm{FS}}}}}\to 0$ is expected, due to the symmetry of *g*_*zx*_(**k**), see Fig. 4c (bottom). Further discussions about possible, extrinsic origins for anti-symmetric contributions are given in the Supplementary Information.

Finally, in the quantum limit, the GNMR is expected to be suppressed as the interband contributions saturate. In this regime, the crystal momentum **k** is no longer a good quantum number, being substituted by the quantized Landau levels *n*. The quantum metric, which previously scaled as *g*_*μν*_(**k**) ~ 1/∣**k**∣^2^, now becomes governed by the magnetic length, ${\ell }_{B}=\sqrt{\hslash /eB}$, and scales as ${g}_{\mu \nu }(B)\propto {\ell }_{B}^{2}\propto 1/B$. At the *n*−th Landau level, the band-splitting scales as $\Delta {E}_{n}(B) \sim \lambda \sqrt{n}/{\ell }_{B} \sim \sqrt{B}$, and therefore the quantum geometric contribution diffusion, ${D}_{ij}\propto {g}_{\mu \nu }{(\Delta {E}_{n})}^{2} \sim n$, saturates, suppressing the GNMR. In the extreme quantum limit, when only the non-degenerate *n* = 0 Landau level is filled, *ΔE*_0_ = 0, and there is no geometric contribution to diffusion.

### The spin-drift locking

The interaction term we put forward in Eq. (5) also admits a simple semiclassical interpretation. For a 2DEG in the *xz* plane and perpendicular magnetic field, ${{{\bf{B}}}}\parallel \widehat{y}$, the energy of an electron is independent of the guiding-center position of the cyclotron orbit, (*X*_0_, *Z*_0_), resulting in a massive degeneracy of *eB*/*h* states per unit area^14^. In the presence of the inversion-breaking polar field, parameterized at low energies by ${{\boldsymbol{{\mathcal{E}}}}}={{{\mathcal{E}}}}\widehat{x}$, this degeneracy is lifted, and the guiding-center orbits acquire a drift along the *Z*_0_ direction, with drift momentum ${{{{\bf{k}}}}}_{d}\propto {{\boldsymbol{{\mathcal{E}}}}}\times {{{\bf{B}}}}$, see Fig. 4d. For a material with strong spin-orbit interaction, *H*_*s*.*o*._ = *λ* **k** ⋅ ***σ***, such as in Te, the drifting Weyl fermions feature a well-defined spin orientation $\langle {{{\boldsymbol{\sigma }}}}\rangle \parallel \langle {{{{\bf{k}}}}}_{d}\rangle \propto {{\boldsymbol{{\mathcal{E}}}}}\times {{{\bf{B}}}}$. This is the spin-drift-momentum locking described by *H*_*DZ*_ (5).

### Comparison with experiments

**Fig. 5:**
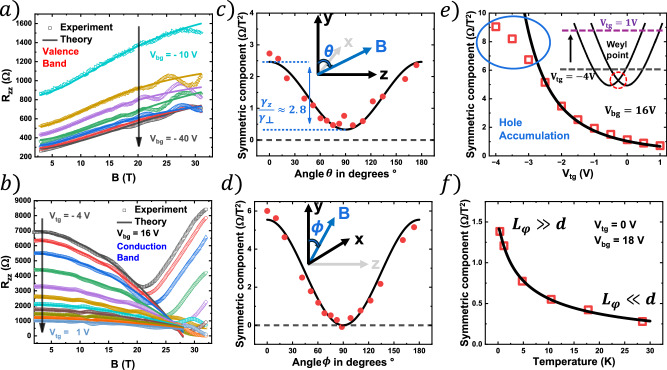
Quantitative analysis of the GNMR in Te. **a** Fit of the PMR for *p*-type carriers in the valence band, from *V*_*bg*_ = −10 V to *V*_*bg*_ = −40 V and *V*_*tg*_ = 0 V, following the expected behavior for two hole accumulation layers. **b** Fit of the NMR for *n*-type carriers in the conduction band, from *V*_*tg*_ = −4 V to *V*_*tg*_ = +4 V and *V*_*bg*_ = +16 V, following the parabolic enhanced diffusion from quantum geometry. **c** Angular evolution of the symmetric component *C*(*θ*) of the GNMR for rotations of the magnetic field, **B**, confined to the *yz* plane. The ratio *C*(0)/*C*(*π*/2) ≈ 7.5 is a direct measure of the anisotropy in the magnetoelectric-spin interaction, *γ*_*z*_/*γ*_⊥_ ≈ 2.8. **d** Angular evolution of *C*(*ϕ*) of the GNMR for rotations of the magnetic field, **B**, confined to the *xy* plane. **e** Evolution of *C*(*θ* = 0) of the GNMR as a function of top-gate voltage, from *V*_*tg*_ = −4 V to *V*_*tg*_ = +1 V, with *V*_*bg*_ = +16 V. The decrease as the Fermi level is tuned away from the Weyl node, shown schematically in the inset, is consistent with a quantum geometric origin. For −4 V < *V*_*tg*_ < −3 V, at the bottom of the conduction band and very close to the Weyl node, hole accumulation occurs, spoiling the agreement between theory and experiment. **f** Evolution of *C*(*θ* = 0) of the GNMR as a function of temperature, from *T* = 0.35 K to *T* = 54 K, at *V*_*bg*_ = +18 V and *V*_*tg*_ = 0 V. The data are well described by $C(T)={C}_{0}/{(1-{\beta }_{wl}ln(T/{T}_{0})+{\alpha }_{ee}\sqrt{T})}^{2}$, describing quantum corrections to conductivity through the dimensional crossover from 2D to 3D, for *L*_*φ*_ ≫ *d* and *L*_*φ*_ ≪ *d*.

Positive magnetoresistance − We used negative back-gate voltages, *V*_*bg*_ < 0, to tune the Fermi level in the valence band of Te. The data for the observed longitudinal PMR shown in Fig. 5a for −40 V < *V*_*bg*_ <−10 V, are well described by the formula ${R}_{zz}(B)={R}_{0}\left(1+\frac{{\sigma }_{1}{\sigma }_{2}{({\mu }_{1}-{\mu }_{2})}^{2}{B}^{2}}{{({\sigma }_{1}+{\sigma }_{2})}^{2}+{({\sigma }_{1}{\mu }_{2}+{\sigma }_{2}{\mu }_{1})}^{2}{B}^{2}}\right)$^29^, which arises from the coexistence of two distinct *p*-type channels of coefficients *μ*_1_, *σ*_1_ and *μ*_2_, *σ*_2_, with *μ*_1_ ≠ *μ*_2_, associated with the two hole accumulation layers at the surfaces of the device^29^.

Negative magnetoresistance  − For the conduction band, we used a fixed positive back-gate voltage, *V*_*bg*_ = +16 V, and variable top-gate voltage, −4V < *V*_*tg*_ <+4 V, to tune the concentration of *n*-type carriers. The longitudinal resistance *R*_*zz*_(*B*) was measured as a function of *B*, *θ*, *V*, and *T*. The data shown in Fig. 5b exhibit a clear NMR whose non-oscillatory part can be fitted using the quantum geometric expression given in Eq. (6), recast as *R*_*zz*_(*B*) = *R*_0_ + *F* ⋅ *B* − *C* ⋅ *B*^2^, with *F* and *C* representing, respectively, the anti-symmetric (in units of Ω/*T*) and symmetric (in units of Ω/*T*^2^) components of the magnetoresistance. Equation (6) predicts that *F* = 0, so any observed anti-symmetric component is entirely due to experimental procedures, such as, for example, sample alignment. As such, in what follows we describe the evolution of the symmetric component (see Supplementary Information for details)

8 $$C=\frac{(L/W)\,{e}^{2}N({E}_{F})\,(8{\tau }_{g}/{\hslash }^{2})\,{\langle {g}_{zz}({{{\bf{k}}}})\rangle }_{{{{\rm{FS}}}}}\,{{{{\mathcal{E}}}}}_{x}^{2}}{{\left({\sigma }_{zz}^{D}+{\sigma }_{zz}^{g}\right)}^{2}}({\gamma }_{z}^{2}{\cos }^{2}{\alpha }_{({{{\bf{B}}}},\widehat{y})}+{\gamma }_{\perp }^{2}{\cos }^{2}{\alpha }_{({{{\bf{B}}}},\widehat{z})}),$$

as a function of ${\alpha }_{({{{\bf{B}}}},\widehat{y})}$ and ${\alpha }_{({{{\bf{B}}}},\widehat{z})}$, *V*, and *T*, where *L* and *W* are the dimensions of the Te film, *N*(*E*_*F*_) is the density of states at the Fermi level, and ${\sigma }_{zz}^{g}$ is the **B** = 0 quantum geometric contribution to conductivity, ${\sigma }_{zz}^{g}=4{e}^{2}N({E}_{F}){\langle ({\lambda }_{\perp }^{2}{k}_{x}^{2}+{\lambda }_{z}^{2}{k}_{z}^{2}){g}_{zz}({{{\bf{k}}}})\rangle }_{{{{\rm{FS}}}}}$.

Angular dependence—For the *y*-*z* rotations, ${\alpha }_{({{{\bf{B}}}},\widehat{y})}=\theta$ and ${\alpha }_{({{{\bf{B}}}},\widehat{z})}=\pi /2-\theta$, the symmetric component *C*(*θ*) in Fig. 5c follows the quantum geometric prediction (8), with $C(\theta )={C}_{z}{\cos }^{2}\theta+{C}_{\perp }{\sin }^{2}\theta$. The ratio ${C}_{z}/{C}_{\perp }\equiv {\gamma }_{z}^{2}/{\gamma }_{\perp }^{2}\approx 7.5$ is a direct measure of the anisotropy in the magnetoelectric-spin interaction, *γ*_*z*_/*γ*_⊥_ ≈ 2.8. For the *y*-*x* rotations, instead, ${\alpha }_{({{{\bf{B}}}},\widehat{y})}=\phi$ and ${\alpha }_{({{{\bf{B}}}},\widehat{z})}=\pi /2$, so *C*(*ϕ*) in Fig. 5d now follows $C(\phi )={C}_{z}{\cos }^{2}\phi$, vanishing at *ϕ* = *π*/2. The angular dependencies described above establish unambiguously that the parabolic GNMR is maximized for ${{{\bf{B}}}}\perp {{\boldsymbol{{\mathcal{E}}}}}$ and vanishes for ${{{\bf{B}}}}\parallel {{\boldsymbol{{\mathcal{E}}}}}$, confirming the structure, $\Delta {R}_{zz}/{R}_{0}\propto -{[\gamma \cdot ({{\boldsymbol{{\mathcal{E}}}}}\times {{{\bf{B}}}})]}^{2}$, predicted by quantum geometry.

Linear contributions—In addition to the symmetric component *C*(*θ*), we also observe a significantly smaller anti-symmetric contribution *F*(*θ*) to the MR under *y*–*z* rotations, which follows a $\cos \theta$ dependence. As discussed in the Supplementary Information, this behavior results from a weak admixture of longitudinal and Hall responses arising from a minute sample misalignment (recall that intrinsic quantum-geometric contributions to the longitudinal MR have been shown to be strictly even in the magnetic field). Conversely, for the *y*–*x* rotations, we detect a small, symmetric and positive MR component ∝ ∣**B**∣ and where *F*(*θ*) follows a $\sin \theta$ dependence. Such behavior is characteristic of an orbital Parish–Littlewood mechanism^32^ driven by charge inhomogeneity, which is expected in tellurene films of finite thickness subject to combined top and back gating^30^. Importantly, this extrinsic positive MR coexists with, but is clearly distinguishable from and much smaller than, the intrinsic quantum-geometric negative MR, which is symmetry-forbidden only for ${{{\bf{B}}}}\parallel {{\boldsymbol{{\mathcal{E}}}}}$.

Voltage dependence—In the limit where the Drude contribution ${\sigma }_{zz}^{D}\gg {\sigma }_{zz}^{g}$, and using a parabolic band approximation in Eq. (8) for the 2D Fermi surface average, ${\langle {g}_{zz}({{{\bf{k}}}})\rangle }_{{{{\rm{FS}}}}}$, we obtain the scaling *C*(*θ* = 0) ∝ *V*^−5/2^ for the voltage dependence of the symmetric component *C*(*θ* = 0, *V*_*bg*_) shown in Fig. 5e (see Supplementary Information for details). This unique dependence with *V* arises from the scaling ${\langle {g}_{zz}({{{\bf{k}}}})\rangle }_{{{{\rm{FS}}}}} \sim 1/\sqrt{n}$, and the fact that $C\propto 1/{({\sigma }_{zz}^{D})}^{2}$, with the Drude conductivity scaling as ${\sigma }_{zz}^{D} \sim n$, and recalling that in 2D (where *N*(*E*_*F*_) is constant) we have *n* ∝ *V*. The rapid decay of *C*(*θ* = 0) with increasing voltage (density) shown in Fig. 5e demonstrates that quantum geometric effects are most pronounced in low-carrier-density regimes, due to the proximity to a Weyl node. However, for −4V < *V*_*tg*_ <−3 V, at the bottom of the conduction band and close to the Weyl node, hole accumulation at the top surface occurs compromising the direct comparison between theory and experiment.

Temperature dependence—The temperature dependence of *C* in Eq. (8) is characteristic of a disordered metal undergoing dimensional crossover, 2D → 3D, when the phase coherence length, *L*_*φ*_ ~ *T*^−1/2^, becomes smaller than the thickness of the film, *d*. The model $C(T)={C}_{0}/{(1-{\beta }_{WL}ln(T/{T}_{0})+{\alpha }_{ee}\sqrt{T})}^{2}$ results from the symmetric component, *C*, being inversely proportional to the square of the Drude conductivity, $C\propto 1/{({\sigma }_{zz}^{D}(T))}^{2}$, with $\delta {\sigma }_{2D}(T)=-{\sigma }_{WL}ln(T/{T}_{0})$ describing the quantum corrections to conductivity due to WL in 2D, valid when *L*_*φ*_ ≫ *d*, and $\delta {\sigma }_{3D}(T)=+ {\sigma }_{ee}\sqrt{T}$ describing the quantum corrections to conductivity due to electron-electron interactions in 3D (when exchange contributions dominate over the Hartree contributions^41^), valid when *L*_*φ*_ ≪ *d*^20^. Although at *T* < 1 K and *V*_*bg*_ = +30 V we find *L*_*φ*_ ~ 500nm^31^, at lower *V*_*bg*_ = +18 V and higher *T* > 20 K the quantum interference phenomena in our *d* = 10−20 nm Te films crosses over from the 2D ($-lnT$) behavior to the 3D ($+\sqrt{T}$) behavior, as can be seen in Fig. 5f.

The remarkable phenomenon of the quantum geometric GNMR reported here signals a new paradigm for quantum transport in 2D materials. Our field rotation and gate voltage dependence studies provide definitive evidence that this effect is not reducible to conventional WL or semiclassical dynamics. Instead, our work establishes a departure from the standard Markovian transport paradigms, revealing a non-trivial memory effect rooted in the wavefunctions’ quantum geometric structure across the Brillouin zone. Our findings suggest that the interplay between the inversion-breaking polar field and the magnetic-field-induced band splitting provides a unique landscape for exploring velocity auto-correlations mediated by the quantum metric. This Drift-Zeeman interaction where the spin degree of freedom is locked to the guiding-center drift represents a fundamental magnetoelectric coupling that transcends conventional Zeeman or orbital effects.

Our work opens new avenues for investigating quantum geometric transport in other non-centrosymmetric Weyl systems and potentially in flat-band materials where the quantum metric plays a primary role in determining the electronic response. Such perspectives position tellurene not merely as a candidate for high-efficiency electronics, but as a prototypical system for studying the deep connections between topology, geometry, and non-equilibrium transport in condensed matter physics. Furthermore, the excellent agreement between our experimental data and our theoretical model of a novel, quantum-geometric-induced Drift-Zeeman interaction opens a new chapter in the study of electron-field interactions in topological quantum matter.

## Methods

### Sample preparation and electrical transport measurements

Two-dimensional Te were synthesized via a hydrothermal growth method. A precursor solution of Na_2_TeO_3_ and polyvinylpyrrolidone (PVP) in deionized water was prepared, followed by the addition of aqueous ammonia and hydrazine hydrate. The mixture was sealed in a Teflon-lined autoclave and reacted at 180 ^∘^C for 12–30 h before being cooled to room temperature. The resulting 2D Te flakes were transferred onto SiO_2_/Si substrates (90 nm thermal oxide) using the Langmuir-Blodgett technique to ensure uniform, clean films. Flake thicknesses ranged from a few atomic layers to 20 nm.

Hall bar devices were fabricated using standard electron beam lithography. Metal contacts (Ni/Au) were deposited via electron beam evaporation. To enable top-gating, a 20 nm Al_2_O_3_ dielectric layer was deposited by atomic layer deposition (ALD) at 200 ^∘^C. The devices exhibited high electronic quality, with tunable carrier densities (*n* ~ 1 × 10^12^ to 1.2 × 10^13^ cm^−2^) and high mobilities (*μ* ~ 6000 cm^2^/V ⋅ s for electrons). Well-defined Shubnikov-de Haas oscillations confirmed low defect concentrations.

Transport measurements were performed in high-field magnet systems (cell 9 and cell 12) at the National High Magnetic Field Laboratory (NHMFL), Florida, USA, with temperatures ranging from 350 mK to 54 K and magnetic fields up to 42 T. Longitudinal (*R*_*zz*_) and Hall (*R*_*zx*_) resistances were measured using phase-sensitive lock-in techniques (SR830/SR860 amplifiers) with low-frequency AC excitation (7−87 Hz) to minimize capacitive coupling.

### Electronic structure and spin texture

**Fig. 6:**
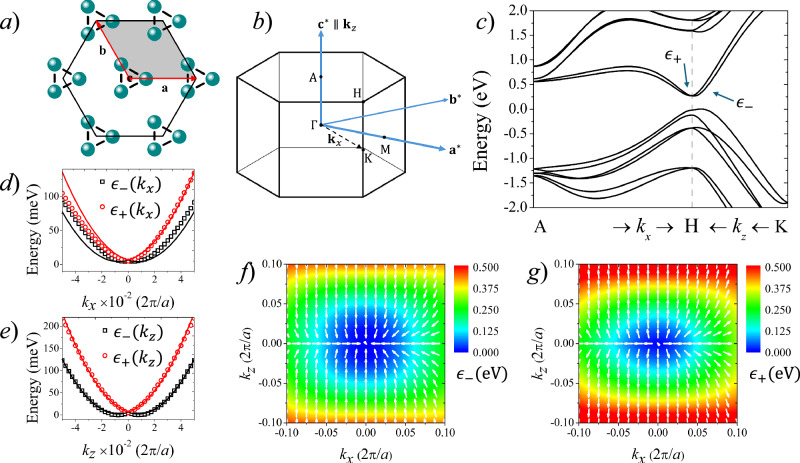
Crystal structure, band structure, and spin texture of Te. **a** Crystal structure of Te, showing the helical atomic chains running along the *z*-axis, parallel to the **c** vector. **b** Hexagonal Brillouin zone of Te with labeled high-symmetry points. **c** Electronic band structure of bulk Te obtained from DFT calculations, highlighting the Weyl node at the H point arising from the crossing of the first two conduction bands, *ϵ*_±_, near their minimum. **d**,** e** Fits of the DFT dispersion relations *ϵ*_±_(*k*_*x*_, *k*_*z*_) to the eigenvalues of the unperturbed model Hamiltonian. The excellent agreement along *k*_*z*_ in (**e**) enables the extraction of the ratio *λ*_*z*_/*λ*_⊥_ ≈ 1.8. In contrast, the poor agreement along *k*_*x*_ in (**d**) reflects the influence of trigonal warping, which is not included in the present model. **f**,** g** Two-dimensional colormap dispersions for the lower band *ϵ*_−_(*k*_*x*_, *k*_*z*_) and the upper band *ϵ*_+_(*k*_*x*_, *k*_*z*_), together with their corresponding radial spin textures, pointing inward for *ϵ*_−_ and outward for *ϵ*_+_.

The crystal structure of Te consists of helical chains running along the *z*-axis (Fig. 6a), with each atom featuring covalent bonds to two neighbors and a lone pair of electrons extending perpendicular to the chain (*x*-axis)^24^. This structure breaks inversion symmetry and generates a significant intrinsic electric polarization field, ${{\boldsymbol{{\mathcal{E}}}}}$, oriented along the *x*-axis. The Brillouin zone is a hexagonal prism (Fig. 6b), and the band structure features a Weyl node near the conduction band minimum at the H point (Fig. 6c). For *n*-type doping, the Fermi level resides in the conduction band, which exhibits linear dispersion and strong spin-orbit coupling, characteristic of a Weyl semiconductor. This leads to substantial Berry curvature and a non-trivial quantum metric. The conduction band is composed by two branches *ε*_±_(*k*_*x*_, *k*_*z*_) shown in Fig. 6d, e for two different directions in the Brillouin zone, and these branches are, in turn, associated with opposite total spin textures, shown in Fig. 6f, g.

Density-functional theory (DFT) calculations were performed within the generalized gradient approximation for the exchange correlation functional using the QUANTUM ESPRESSO package^42^, with fully relativistic Perdew-Burke-Ernzerhof pseudopotentials to account for spin-orbit coupling. Optimal structural and convergence parameters were taken from Ref. ^41^, adopting lattice constants *a* = 4.767 Å and *c*/*a* = 1.2447 for the hexagonal cell. The atoms form helical chains along *z*, with three atoms per unit cell separated by *u* = 0.245*a*. For the self-consistent calculations, a dense 24 × 24 × 20 Monkhorst Pack *k*-point mesh and a plane-wave cutoff energy of 50Ry were employed. For the band-structure and spin-texture calculations, a fine mesh of 1 × 90 × 80 *k*-points around the H point was used to generate the two-dimensional electronic and spin maps, as well as the dispersions along *k*_*x*_ and *k*_*z*_.

To extract the ratio *λ*_*z*_/*λ*_⊥_, we fit the DFT conduction bands to the analytical eigenvalues of Eq. (4), given by

9 $${\epsilon }_{\pm }({{{\bf{k}}}})=\frac{{\hslash }^{2}{k}_{x}^{2}}{2{m}_{\perp }^{*}}+\frac{{\hslash }^{2}k{z}^{2}}{2{m}_{z}^{*}}\pm \sqrt{{\lambda }_{\perp }^{2}{k}_{x}^{2}+{\lambda }_{z}^{2}{k}_{z}^{2}},$$

where the wave-vector components are defined with respect to the H point.

## Supplementary information

Supplementary Information

Transparent Peer Review file

## Data Availability

The source data supporting the figures and conclusions of this study have been deposited in Zenodo at 10.5281/zenodo.20300792. The deposited archive contains Excel spreadsheets with the numerical data used to generate the main text and Supplementary Figs., together with fitting parameters used in the analysis.

## Code availability

No custom software was developed for this study. The analyses and figure preparation were carried out using standard Mathematica routines. The numerical source data required to reproduce the figures are available in the Zenodo repository listed in the Data Availability statement.
