# Supplementary material for: Gate-tunable giant negative magnetoresistance in tellurene driven by quantum geometry
Source: Nat Commun. 2026 Jun 5;17:5783. doi: 10.1038/s41467-026-74066-0 (PMC13328452; doi:10.1038/s41467-026-74066-0)
Supplement: Supplementary file 1 — Supplementary Information [file 41467_2026_74066_MOESM1_ESM.pdf]

Supplementary Information to Gate-Tunable Giant Negative Magnetoresistance in Tellurene Driven by Quantum Geometry

Marcello B. Silva Neto[1*†], Chang Niu[2,3†],
Marcus V. O. Moutinho[4], Pierpaolo Fontana[5], Claudio Iacovelli[6],
Victor Velasco[7], Caio Lewenkopf[1], Peide D. Ye[2,3*]

[1]Instituto de Física, Universidade Federal do Rio de Janeiro, Rio de janeiro, 21941-972, RJ, Brazil.
[2]Elmore Family School of Electrical and Computer Engineering, Purdue University, West Lafayette, 47907, Indiana, United States.
[3]Birck Nanotechnology Center, Purdue University, West Lafayette, 47907, Indiana, United States.
[4]Universidade Federal do Rio de Janeiro - Campus Duque de Caxias, Duque de Caxias, 25240-005, RJ, Brazil.
[5]Departament de Física, Universitat Autònoma de Barcelona, 08193, Bellaterra, Spain.
[6]Independent Researcher, Barcelona, 08193, Spain.
[7]International School for Advanced Studies (SISSA), Trieste, I-34136, Italy.

*Corresponding author(s). E-mail(s): mbsn@if.ufrj.br; yep@purdue.edu;
Contributing authors: niu43@purdue.edu; moutinho@xerem.ufrj.br; pierpaolo.fontana@uab.cat; claudio.iacovelli@hotmail.it; vvelasco@sissa.it; lewenkopf@if.ufrj.br;
†These authors contributed equally.

1 Geometric Diffusion

In semiclassical transport, the diffusion tensor D_{ij} is related to the integrated auto-velocity correlation

$$D_{ij} = \int_0^\infty \langle v_i(t) v_j(0) \rangle \, dt, \tag{1}$$

where $v_i = \partial_{k_i} \epsilon(\mathbf{k})/\hbar$ is the band velocity for simple bands, but can be generalized to include geometric effects. The quantum metric enhances velocity fluctuations δv because it encodes the spread of the wavefunction in momentum space, and will contribute an additional variance

$$\langle \delta v_i \delta v_j \rangle \sim g_{ij}(\mathbf{k}). \tag{2}$$

This arises because the quantum metric governs the overlap fluctuations between states at nearby **k**-points. In systems with strong geometric effects (e.g., twisted bilayer graphene, topological insulators, or Dirac/Weyl materials), this leads to diffusion beyond the Drude model.

1.1 Velocity auto-correlation and quantum metric

To derive the velocity variance $\langle \delta v_i \delta v_j \rangle$ from the quantum metric $g_{ij}(\mathbf{k})$, we start from the geometric structure of Bloch states in momentum space and connect it to velocity fluctuations. The velocity operator for a Bloch electron is given by the derivative of $\mathcal{H}(\mathbf{k})$

$$v_i = \frac{1}{\hbar} \partial_{k_i} \mathcal{H}(\mathbf{k}). \tag{3}$$

Its matrix elements between $|u_n(\mathbf{k})\rangle$ and $|u_m(\mathbf{k})\rangle$ are

$$v_i^{nm}(\mathbf{k}) = \frac{1}{\hbar} \langle u_n(\mathbf{k}) | \partial_{k_i} \mathcal{H}(\mathbf{k}) | u_m(\mathbf{k}) \rangle. \tag{4}$$

For an eigenstate $|u_n(\mathbf{k})\rangle$ of $\mathcal{H}(\mathbf{k})$ with eigenvalue $\epsilon_n(\mathbf{k})$

$$\partial_{k_i} \epsilon_n(\mathbf{k}) = \langle u_n(\mathbf{k}) | \partial_{k_i} \mathcal{H}(\mathbf{k}) | u_n(\mathbf{k}) \rangle. \tag{5}$$

These correspond to the intraband matrix elements.

However, for interband elements ($n \neq m$), we need to extend our calculation. We start with the equation

$$\mathcal{H}(\mathbf{k}) | u_m(\mathbf{k}) \rangle = \epsilon_m(\mathbf{k}) | u_m(\mathbf{k}) \rangle. \tag{6}$$

Then we take the derivative with respect to k_i

$$(\partial_{k_i} \mathcal{H}) | u_m \rangle + \mathcal{H} | \partial_{k_i} u_m \rangle = (\partial_{k_i} \epsilon_m) | u_m \rangle + \epsilon_m | \partial_{k_i} u_m \rangle. \tag{7}$$

Next, we multiply by $\langle u_n|$ (for $n \neq m$)

$$\langle u_n|\partial_{k_i}\mathcal{H}|u_m\rangle + \langle u_n|\mathcal{H}|\partial_{k_i}u_m\rangle = \epsilon_m\langle u_n|\partial_{k_i}u_m\rangle. \tag{8}$$

Since $\mathcal{H}\langle u_n| = \epsilon_n\langle u_n|$, the second term becomes

$$\langle u_n|\mathcal{H}|\partial_{k_i}u_m\rangle = \epsilon_n\langle u_n|\partial_{k_i}u_m\rangle. \tag{9}$$

We can now substitute back to arrive at

$$\langle u_n|\partial_{k_i}\mathcal{H}|u_m\rangle = (\epsilon_m - \epsilon_n)\langle u_n|\partial_{k_i}u_m\rangle. \tag{10}$$

Thus, the velocity matrix element is

$$v_i^{nm}(\mathbf{k}) = \frac{1}{\hbar}(\epsilon_m - \epsilon_n)\langle u_n|\partial_{k_i}u_m\rangle. \tag{11}$$

The factor $(\epsilon_m - \epsilon_n)$ indicates that transitions between energetically separated bands contribute more strongly to velocity fluctuations, while the overlap $\langle u_n|\partial_{k_i}u_m\rangle$ is tied to the quantum metric $g_{ij}(\mathbf{k})$ when summed over virtual transitions.

The velocity variance $\langle\delta v_i\delta v_j\rangle$ arises from transitions to other bands and is tied to the quantum metric $g_{ij}(\mathbf{k})$. The quantum metric for a single band n is defined as

$$g_{ij}(\mathbf{k}) = \mathrm{Re}\left[\langle\partial_{k_i}u_n|\partial_{k_j}u_n\rangle - \langle\partial_{k_i}u_n|u_n\rangle\langle u_n|\partial_{k_j}u_n\rangle\right]. \tag{12}$$

This quantity measures the distance between nearby Bloch states in Hilbert space. The term $\langle\partial_{k_i}u_n|\partial_{k_j}u_n\rangle$ can be expanded using the completeness relation

$$\sum_m |u_m\rangle\langle u_m| = 1, \tag{13}$$

where m runs over all bands. Now

$$\langle\partial_{k_i}u_n|\partial_{k_j}u_n\rangle = \sum_m\langle\partial_{k_i}u_n|u_m\rangle\langle u_m|\partial_{k_j}u_n\rangle. \tag{14}$$

This includes both the term where $m = n$ and terms where $m \neq n$. The sum is now split in two parts

$$\begin{aligned}\langle\partial_{k_i}u_n|\partial_{k_j}u_n\rangle &= \langle\partial_{k_i}u_n|u_n\rangle\langle u_n|\partial_{k_j}u_n\rangle \\ &+ \sum_{m\neq n}\langle\partial_{k_i}u_n|u_m\rangle\langle u_m|\partial_{k_j}u_n\rangle.\end{aligned} \tag{15}$$

The first term $\langle\partial_{k_i}u_n|u_n\rangle\langle u_n|\partial_{k_j}u_n\rangle$ is the product of Berry connections (phase-related terms). The second term $\sum_{m\neq n}$ captures interband transitions. The Berry connection

terms cancel out, leaving us with

$$g_{ij}(\mathbf{k}) = \text{Re}\left[\sum_{m\neq n} \langle \partial_{k_i} u_n | u_m \rangle \langle u_m | \partial_{k_j} u_n \rangle \right]. \tag{16}$$

This shows that the quantum metric is entirely determined by interband transitions ($m \neq n$), reflecting the geometry of the Bloch states.

From the velocity operator expression, the off-diagonal elements ($m \neq n$) are

$$v_i^{nm}(\mathbf{k}) = \frac{\epsilon_m - \epsilon_n}{\hbar} \langle u_n | \partial_{k_i} u_m \rangle. \tag{17}$$

Thus, the product of velocity fluctuations is

$$v_i^{nm} v_j^{mn} = \frac{\epsilon_m - \epsilon_n}{\hbar} \frac{\epsilon_n - \epsilon_m}{\hbar} \langle u_n | \partial_{k_i} u_m \rangle \langle u_m | \partial_{k_j} u_n \rangle, \tag{18}$$

and $(\epsilon_m - \epsilon_n)(\epsilon_n - \epsilon_m) = -(\epsilon_m - \epsilon_n)^2$. We can further simplify this expression by using that $\langle u_n | u_m \rangle = 0$ for $n \neq m$ to write

$$\partial_{k_i} \langle u_n | u_m \rangle = 0 \implies \langle \partial_{k_i} u_n | u_m \rangle = -\langle u_n | \partial_{k_i} u_m \rangle, \tag{19}$$

and the product of velocity fluctuations becomes

$$v_i^{nm} v_j^{mn} = \left(\frac{\epsilon_m - \epsilon_n}{\hbar} \right)^2 \langle \partial_{k_i} u_n | u_m \rangle \langle u_m | \partial_{k_j} u_n \rangle. \tag{20}$$

1.2 The case of a two-band Hamiltonian

We now specialize to a general two-band Hamiltonian with eigenstates $|+\rangle$ and $|-\rangle$. The product of interband velocity matrix elements is

$$v_i^{-+} v_j^{+-} = \left(\frac{\epsilon_+ - \epsilon_-}{\hbar} \right)^2 \langle \partial_{k_i} u_- | u_+ \rangle \langle u_+ | \partial_{k_j} u_- \rangle. \tag{21}$$

Here v_i^{-+} is the velocity matrix element from the lower band $(-)$ to the upper band $(+)$, $\epsilon_\pm$ are the eigenstates for the upper/lower bands, and $\langle \partial_{k_i} u_- | u_+ \rangle$ is the overlap between the k_i-derivative of $|u_-\rangle$ and $|u_+\rangle$.

In a two-band system, the only virtual transition contributing to the velocity variance is $(-) \leftrightarrow (+)$. Thus, the total variance is

$$\langle \delta v_i \delta v_j \rangle = v_i^{-+} v_j^{+-} + v_i^{+-} v_j^{-+}. \tag{22}$$

However, since $v_i^{+-} = (v_i^{-+})^*$ (Hermiticity of the velocity operator), we can write

$$\langle \delta v_i \delta v_j \rangle = v_i^{-+} v_j^{+-} + \text{c.c.} = 2\,\mathrm{Re}\left[v_i^{-+} v_j^{+-}\right]. \tag{23}$$

Substituting now

$$\langle \delta v_i \delta v_j \rangle = 2\,\mathrm{Re}\left[\left(\frac{\Delta\epsilon}{\hbar}\right)^2 \langle \partial_{k_i} u_- | u_+ \rangle \langle u_+ | \partial_{k_j} u_- \rangle\right], \tag{24}$$

where $\Delta\epsilon = \epsilon_+ - \epsilon_-$. The quantum metric $g_{ij}(\mathbf{k})$ for the lower band ($|u_-\rangle$) is

$$g_{ij}(\mathbf{k}) = \mathrm{Re}\left[\langle \partial_{k_i} u_- | \partial_{k_j} u_- \rangle - \langle \partial_{k_i} u_- | u_- \rangle \langle u_- | \partial_{k_j} u_- \rangle\right]. \tag{25}$$

For a two-band system, the completeness relation is

$$|u_+\rangle\langle u_+| + |u_-\rangle\langle u_-| = 1. \tag{26}$$

Thus, the first term expands as

$$\begin{aligned} \langle \partial_{k_i} u_- | \partial_{k_j} u_- \rangle &= \langle \partial_{k_i} u_- | u_+ \rangle \langle u_+ | \partial_{k_j} u_- \rangle \\ &+ \langle \partial_{k_i} u_- | u_- \rangle \langle u_- | \partial_{k_j} u_- \rangle. \end{aligned} \tag{27}$$

Substituting into $g_{ij}(\mathbf{k})$

$$g_{ij}(\mathbf{k}) = \mathrm{Re}\left[\langle \partial_{k_i} u_- | u_+ \rangle \langle u_+ | \partial_{k_j} u_- \rangle\right]. \tag{28}$$

We can now combine the results

$$\begin{aligned} \langle \delta v_i \delta v_j \rangle &= 2\left(\frac{\Delta\epsilon}{\hbar}\right)^2 \mathrm{Re}\left[\langle \partial_{k_i} u_- | u_+ \rangle \langle u_+ | \partial_{k_j} u_- \rangle\right] \\ &= 2\left(\frac{\Delta\epsilon}{\hbar}\right)^2 g_{ij}(\mathbf{k}). \end{aligned} \tag{29}$$

The total velocity variance (averaged over all virtual transitions) for a two-band model becomes simply

$$\langle \delta v_i \delta v_j \rangle = 2\frac{(\Delta\epsilon)^2}{\hbar^2} g_{ij}(\mathbf{k}). \tag{30}$$

The quantum metric $g_{ij}(\mathbf{k})$ thus quantifies the geometric spread of Bloch states in momentum space, leading to intrinsic velocity fluctuations that contribute to diffusion beyond the Drude picture.

2 Multiband origin of the inversion-odd interactions

The giant negative magnetoresistance (GNMR) is controlled by a magnetic-field-induced splitting of the conduction bands. To place the microscopic origin of this splitting on a controlled footing, we start from the multiband crystal Hamiltonian

$$H = \frac{(\mathbf{p} + e\mathbf{A})^2}{2m} + V(\mathbf{r}) + \frac{\hbar}{4m^2c^2}\left[\nabla V(\mathbf{r}) \times (\mathbf{p} + e\mathbf{A})\right] \cdot \boldsymbol{\sigma}^{(s)}, \tag{31}$$

where e is the electron charge, m is the bare electron mass, c is the speed of light, and $\boldsymbol{\sigma}^{(s)}$ denotes the physical spin-$\frac{1}{2}$ operator in the full multiband Hilbert space. The periodic crystal potential is decomposed into inversion-even and -odd parts,

$$V(\mathbf{r}) = V_{\text{even}}(\mathbf{r}) + V_{\text{odd}}(\mathbf{r}). \tag{32}$$

The inversion-odd part is responsible for the polar character of the crystal and ultimately for the inversion-odd terms in the projected low-energy theory. The observation of nearly tilt-independent Shubnikov-de-Haas oscillation frequencies is consistent with a g-factor that is negligibly small for the conduction bands, and therefore we omit a separate bare Zeeman term proportional to $-g\mu_B \mathbf{B} \cdot \boldsymbol{\sigma}^{(s)}$, where μ_B is the Bohr magneton.

2.1 Microscopic decomposition and matrix elements

We introduce the Bloch functions centered at the H-point in the Brillouin zone, and write the crystal Hamiltonian accordingly, introducing the continuous crystal momentum $\mathbf{k}$ vector [1]. The resulting Hamiltonian is partitioned into an unperturbed term

$$H_0 = \frac{\mathbf{p}^2}{2m} + V_{\text{even}}(\mathbf{r}), \tag{33}$$

and a perturbation $H_{\text{pert}} = H_{kp} + H_{\Pi} + H_{\text{inv}} + H_B^{\text{so}} + H_B^{\text{orb}}$, where

$$H_{kp} = \frac{\hbar}{m}\mathbf{k} \cdot \mathbf{p}, \tag{34}$$

$$H_{\Pi} = V_{\text{odd}}(\mathbf{r}), \tag{35}$$

$$H_{\text{inv}} = \frac{\hbar}{4m^2c^2}\left(\nabla V_{\text{odd}} \times \mathbf{p}\right) \cdot \boldsymbol{\sigma}^{(s)}, \tag{36}$$

$$H_B^{\text{so}} = \frac{e\hbar}{4m^2c^2}\left[\nabla V(\mathbf{r}) \times \mathbf{A}\right] \cdot \boldsymbol{\sigma}^{(s)}, \tag{37}$$

$$H_B^{\text{orb}} = \frac{e}{2m}\{\mathbf{A}, \mathbf{p} + \hbar\mathbf{k}\}, \tag{38}$$

In Eq. (38), the curly brackets denote the anticommutator $\{\mathbf{A}, \mathbf{p}+\hbar\mathbf{k}\} = \mathbf{A}\cdot(\mathbf{p}+\hbar\mathbf{k})+(\mathbf{p}+\hbar\mathbf{k})\cdot\mathbf{A}$, and we adopt the symmetric gauge $\mathbf{A} = \frac{1}{2}\mathbf{B}\times\mathbf{r}$. Among the perturbations, we neglect the term proportional to $(\nabla V(\mathbf{r})\times\mathbf{k})\cdot\boldsymbol{\sigma}^{(s)}$, as it yields higher-order $\mathbf{k}$-terms in the effective Hamiltonian even in the absence of electromagnetic fields [1, 2].

We now introduce the matrix elements that enter the Löwdin downfolding introduced in the next subsection (see 2.2). Let $\{|u_a\rangle\}$, with $a = \pm$, denote the conduction-band Kramers doublet. The projector onto this 2×2 low-energy subspace is

$$P = \sum_{a=\pm} |u_a\rangle\langle u_a|. \tag{39}$$

Upon Löwdin projection, the physical spin operator $\boldsymbol{\sigma}^{(s)}$ is represented within the two-dimensional doublet subspace as

$$P\,\sigma_i^{(s)}\,P = s_{i0}\,\mathbb{I}_2 + \sum_j s_{ij}\sigma_j, \tag{40}$$

and the projection coefficients s_{ij} are subsequently absorbed into the effective low-energy couplings. To maintain clarity, we denote the pseudospin Pauli matrices acting in the projected Kramers doublet as $\boldsymbol{\sigma}$, thereby distinguishing them from the physical electron spin operator $\boldsymbol{\sigma}^{(s)}$ introduced in Eq. (31). Finally, we define the projector onto the complementary subspace of remote-bands as

$$Q = 1 - P = \sum_{n\in Q} |n\rangle\langle n|, \tag{41}$$

where $|n\rangle$ represents any state outside the conduction-band doublet.

$\boldsymbol{H_{kp}}$ – matrix elements

The $\mathbf{k}\cdot\mathbf{p}$ matrix elements are simply

$$\langle u_a|H_{kp}|n\rangle = \frac{\hbar}{m}\sum_i k_i\,\langle u_a|p_i|n\rangle. \tag{42}$$

$\boldsymbol{H}_{\mathbf{inv}}$ – matrix elements

To treat the inversion-breaking spin-orbit operator, it is convenient to choose a phase convention where the orbital wavefunctions are real. Since $p_j = -i\hbar\partial_j$, the inter-band matrix elements of p_j between states of opposite-parity are commonly purely imaginary. Consequently, we define the real vector $\boldsymbol{\mathcal{P}}^{(n,a)}$ as

$$\mathcal{P}_k^{(n,a)} \equiv -\frac{i\hbar}{4m^2c^2}\sum_{ij}\epsilon_{ijk}\langle u_a|(\partial_i V_{\mathrm{odd}})p_j|n\rangle, \tag{43}$$

so that the purely imaginary matrix elements can be written as

$$\langle u_a|H_{\mathrm{inv}}|n\rangle = i\,\boldsymbol{\mathcal{P}}^{(n,a)}\cdot\boldsymbol{\sigma}. \tag{44}$$

Matrix elements of the form in Eq. (44) are reorganized as operator-valued matrix elements within the retained two-dimensional low-energy subspace. After projection,

the action of the microscopic spin operator $\boldsymbol{\sigma}^{(s)}$ is already encoded in the multiband matrix element. The resulting operator acting within the conduction-band Kramers doublet is then expanded in the Pauli basis $\boldsymbol{\sigma}$ of the effective pseudospin, following Eq. (40), with the orbital and interband contributions absorbed into the coefficients $\mathcal{P}^{(n,a)}$.

$\boldsymbol{H_B^{\text{so}}}$ – matrix elements

Similarly, the spin–orbit operator can be decomposed as

$$H_B^{\text{so}} = \sum_b U_b(\mathbf{B})\,\sigma_b^{(s)}, \qquad U_b(\mathbf{B}) = \frac{e\hbar}{4m^2c^2}\sum_{ij}\epsilon_{ijb}\,(\partial_i V)\,A_j. \tag{45}$$

Since $\mathbf{A} = \frac{1}{2}\mathbf{B}\times\mathbf{r}$, this operator is linear in the magnetic field. Adopting the same real-orbital basis used above, we define the real, B-linear spin–orbit matrix element as

$$\mathcal{M}_b^{(n,a)}(\mathbf{B}) \equiv \langle n|U_b(\mathbf{B})|u_a\rangle = \frac{e\hbar}{8m^2c^2}\sum_{ij\ell m}\epsilon_{ijb}\epsilon_{j\ell m}\,\langle n|(\partial_i V)\,r_m|u_a\rangle\,B_\ell. \tag{46}$$

Using this definition, the matrix elements of H_B^{so} within the low-energy subspace are succinctly expressed as

$$\langle n|H_B^{\text{so}}|u_a\rangle = \boldsymbol{\mathcal{M}}^{(n,a)}(\mathbf{B})\cdot\boldsymbol{\sigma}. \tag{47}$$

where the transformation of the physical spin $\boldsymbol{\sigma}^{(s)}$ into the pseudospin basis $\boldsymbol{\sigma}$ is implicitly handled by the projection coefficients, as established in Eq. (40).

$\boldsymbol{H_\Pi}$ – matrix elements

The scalar inversion-breaking matrix elements are written as

$$\langle u_a|H_\Pi|n\rangle = \boldsymbol{\Pi}\cdot\mathbf{D}^{(n,a)}, \qquad \mathbf{D}^{(n,a)} \equiv \langle u_a|\mathbf{r}|n\rangle, \tag{48}$$

where $\boldsymbol{\Pi}$ is the projected polar strength due to the inversion-odd crystal potential.

$\boldsymbol{H_{B,k}^{\text{orb}}}$ – matrix elements

Finally, the $\mathbf{k}$-dependent part of the orbital magnetic perturbation has matrix elements

$$\mathcal{N}^{(n)}(\mathbf{B},\mathbf{k}) \equiv \langle n|H_{B,k}^{\text{orb}}|u_a\rangle = \frac{e\hbar}{2m}\,\mathbf{k}\cdot(\mathbf{B}\times\mathbf{D}^{(n,a)}) = -\frac{e\hbar}{2m}\,\mathbf{D}^{(n,a)}\cdot(\mathbf{B}\times\mathbf{k}). \tag{49}$$

2.2 General Löwdin downfolding

The effective low-energy Hamiltonian for the conduction-band subspace is derived using a Löwdin (Schrieffer–Wolff) transformation. The general form of the projected Hamiltonian reads

$$H_{\text{eff}} = PHP + \frac{1}{2}P[S, H_{\text{pert}}]P, \qquad [H_0, S] = H_{\text{pert,off}}, \tag{50}$$

where $H_{\text{pert,off}}$ denotes the components of the perturbation that couple the P and Q subspaces. For a given perturbation X, the corresponding generator S_X is defined as

$$S_X = \frac{QH_XP}{\Delta} - \frac{PH_XQ}{\Delta}, \tag{51}$$

where $\Delta = E_P - E_Q$ denotes the relevant interband energy denominator associated with virtual transitions between the retained conduction doublet P and the remote-band subspace Q.

We now apply this framework to derive the low-energy Hamiltonian presented in the main text. We proceed in four steps and calculate: *(i)* the zero-field $\mathbf{k}\cdot\boldsymbol{\sigma}$ sector (anisotropic Weyl Hamiltonian), *(ii)* the spin-dependent B–linear sector (Drift–Zeeman term), *(iii)* the spin-scalar, k–dependent and linear in B sector (Drift–Orbit term) and *(iv)* all remaining subleading terms.

2.2.1 Zero-field projected Hamiltonian: anisotropic Weyl term

At zero magnetic field, the leading odd-in-momentum spin splitting in the projected conduction subspace arises from the mixed second-order process

$$H_{\text{eff}}^{(0)} = PH_{kp}Q\frac{1}{E_c - QH_0Q}QH_{\text{inv}}P + PH_{\text{inv}}Q\frac{1}{E_c - QH_0Q}QH_{kp}P. \tag{52}$$

where E_c denotes the energy of the conduction-band doublet at the expansion point. Using Eqs. (42) and (44), one obtains a projected operator linear in both $\mathbf{k}$ and $\boldsymbol{\sigma}$,

$$H_{\text{eff}}^{(0)}(\mathbf{k}) = \varepsilon_0(\mathbf{k})\,\mathbb{I}_2 + \sum_{ij}\beta_{ij}k_i\sigma_j, \tag{53}$$

where $\varepsilon_0(\mathbf{k})$ denotes the spin-independent part of the effective conduction-band dispersion, and

$$\beta_{ij} \sim \frac{\hbar^2}{4m^3c^2}\sum_{a=\pm}\sum_{n\in Q}\frac{\langle u_a|p_i|n\rangle\langle n|(\nabla V_{\text{odd}}\times\mathbf{p})_j|u_a\rangle + \text{h.c.}}{\Delta_n}. \tag{54}$$

where $\Delta_n \equiv E_c - E_n$ is the interband energy denominator associated with virtual transitions from the conduction-band doublet to the remote band $|n\rangle$. This result shows that the zero-field Weyl-like spin texture is generated by the interference between one $\mathbf{k}\cdot\mathbf{p}$ transition and one inversion-breaking spin-orbit transition mediated by remote bands. As discussed in Sec. 2.3, for bulk tellurium, point-group symmetry constrains β_{ij} to the anisotropic form [1]

$$\beta_{ij} = \text{diag}(\lambda_\perp, \lambda_\perp, \lambda_z), \tag{55}$$

yielding the effective Hamiltonian

$$H_{\rm eff}^{(0)}(\mathbf{k}) = \varepsilon_0(\mathbf{k})\,\mathbb{I}_2 + \lambda_\perp(k_x\sigma_x + k_y\sigma_y) + \lambda_z k_z\sigma_z. \tag{56}$$

Parametrically, the coupling constants scale as

$$\lambda \sim \frac{P_k\,\Lambda_{\rm SO}}{\Delta}, \tag{57}$$

where $P_k \sim (\hbar/m)\langle u_a|p|n\rangle$ denotes a characteristic $\mathbf{k}\cdot\mathbf{p}$ matrix-element, while $\Lambda_{\rm SO} \sim \frac{\hbar}{4m^2c^2}\langle n|(\nabla V_{\rm odd}\times\mathbf{p})|u_a\rangle$ is the inversion-breaking spin-orbit scale.

2.2.2 Generalized Drift–Zeeman sector

We now introduce the magnetic field via minimal coupling and collect all mixed second-order channels that generate spin-dependent terms linear in both the polar strength and the magnetic field. The resulting projected operator has the general form

$$\delta H_{\rm eff}^{\rm DZ} = \sum_{kij}\gamma_{kij}\,\Pi_i B_j\,\sigma_k. \tag{58}$$

where Π_i denote the Cartesian coodinates of the projected polar-strength vector $\boldsymbol{\Pi}$, and γ_{kij} is the generalized Drift–Zeeman tensor, to be properly defined below. Three distinct microscopic channels contribute to this symmetry class

$$\delta H_{\rm eff}^{({\rm inv},so)} = \frac{1}{2}P\Big([S_{\rm inv}, H_B^{\rm so}] + [S_B^{\rm so}, H_{\rm inv}]\Big)P, \tag{59}$$

$$\delta H_{\rm eff}^{(\Pi,so)} = \frac{1}{2}P\Big([S_\Pi, H_B^{\rm so}] + [S_B^{\rm so}, H_\Pi]\Big)P, \tag{60}$$

$$\delta H_{\rm eff}^{({\rm inv},orb-p)} = \frac{1}{2}P\Big([S_{\rm inv}, H_{B,p}^{\rm orb}] + [S_{B,p}^{\rm orb}, H_{\rm inv}]\Big)P, \tag{61}$$

where $H_{B,p}^{\rm orb}$ denotes the part of $H_B^{\rm orb}$ proportional to $\{\mathbf{A},\mathbf{p}\}$.

To illustrate the underlying physics, we first focus on the $H_{\rm inv}\times H_B^{\rm so}$ channel, where the Pauli-matrix algebra is more transparent. Using the matrix elements defined in Eqs. (44) and (46), this contribution is expressed as

$$\delta H_{\rm eff}^{({\rm inv},so)} = -\sum_{n\in Q}\frac{1}{\Delta_n}{\rm Re}\Big[i\big(\boldsymbol{\mathcal{P}}^{(n)}\cdot\boldsymbol{\sigma}\big)\big(\boldsymbol{\mathcal{M}}^{(n)}(\mathbf{B})\cdot\boldsymbol{\sigma}\big)\Big]. \tag{62}$$

Using the Pauli identity $(\mathbf{A}\cdot\boldsymbol{\sigma})(\mathbf{B}\cdot\boldsymbol{\sigma}) = \mathbf{A}\cdot\mathbf{B}\,\mathbb{I}_2 + i(\mathbf{A}\times\mathbf{B})\cdot\boldsymbol{\sigma}$, we obtain

$$i(\boldsymbol{\mathcal{P}}\cdot\boldsymbol{\sigma})(\boldsymbol{\mathcal{M}}\cdot\boldsymbol{\sigma}) = i\,\boldsymbol{\mathcal{P}}\cdot\boldsymbol{\mathcal{M}}\,\mathbb{I}_2 - (\boldsymbol{\mathcal{P}}\times\boldsymbol{\mathcal{M}})\cdot\boldsymbol{\sigma}. \tag{63}$$

In the real-orbital phase convention adopted here, the scalar product $\boldsymbol{\mathcal{P}}\cdot\boldsymbol{\mathcal{M}}$ is real and therefore the first term in Eq. (63) is purely imaginary. The real part filters out

this spin-scalar term, leaving

$$\delta H_{\text{eff}}^{(\text{inv},so)} = \sum_{n\in Q} \frac{1}{\Delta_n} \big(\boldsymbol{\mathcal{P}}^{(n)} \times \boldsymbol{\mathcal{M}}^{(n)}(\mathbf{B})\big) \cdot \boldsymbol{\sigma}. \tag{64}$$

This identifies the origin of the Drift–Zeeman interaction discussed in the main text.

To express this term and the remaining channels in a unified tensor notation, we group together

$$\begin{aligned} \mathcal{P}_b^{(n)} &= \sum_i C_{bi}^{(n)} \Pi_i, & \Pi^{(n)} &= \sum_i \mathcal{D}_i^{(n)} \Pi_i, \\ \mathcal{M}_c^{(n)}(\mathbf{B}) &= \sum_j \mathbb{M}_{cj}^{(n)} B_j, & \mathcal{O}^{(n)}(\mathbf{B}) &= \sum_j \mathcal{O}_j^{(n)} B_j. \end{aligned} \tag{65}$$

Here $C_{bi}^{(n)}$ controls the linear dependence of the inversion-breaking spin-orbit matrix element on the polar strength, $\mathcal{D}_i^{(n)}$ is the scalar polar matrix element associated with H_Π, $\mathbb{M}_{cj}^{(n)}$ is the B-linear spin-orbit tensor associated with H_B^{so}, and $\mathcal{O}_j^{(n)}$ is the scalar orbital magnetic matrix element derived from $H_{B,p}^{\text{orb}}$.

The generalized Drift–Zeeman tensor γ_{kij} is the sum of three distinct contributions

$$\gamma_{kij} = \gamma_{kij}^{(\text{inv},so)} + \gamma_{kij}^{(\Pi,so)} + \gamma_{kij}^{(\text{inv},orb-p)}, \tag{66}$$

with explicit tensorial forms given by

$$\gamma_{kij}^{(\text{inv},so)} = \sum_{n\in Q} \frac{1}{\Delta_n} \sum_{bc} \epsilon_{kbc}\, C_{bi}^{(n)}\, \mathbb{M}_{cj}^{(n)}, \tag{67}$$

$$\gamma_{kij}^{(\Pi,so)} = \sum_{n\in Q} \frac{1}{\Delta_n} \mathcal{D}_i^{(n)}\, \mathbb{M}_{kj}^{(n)}, \tag{68}$$

$$\gamma_{kij}^{(\text{inv},orb-p)} = \sum_{n\in Q} \frac{1}{\Delta_n} C_{ki}^{(n)}\, \mathcal{O}_j^{(n)}. \tag{69}$$

Equation (67) provides the tensor representation of the cross-product structure, $H_{\text{inv}} \times H_B^{\text{so}}$ channel, derived above. Meanwhile, Eqs. (68) and (69) describe the two additional channels that renormalize the same spin-dependent tensor structure.

2.2.3 Drift–Orbit sector

The generalized Drift–Orbit term is the spin-scalar contribution that is linear in the inversion-breaking polar strength, the magnetic field, and the crystal momentum. It arises from the mixed scalar channel

$$\delta H_{\text{eff}}^{\text{DO}} = \frac{1}{2} P\Big([S_\Pi, H_{B,k}^{\text{orb}}] + [S_{B,k}^{\text{orb}}, H_\Pi]\Big) P. \tag{70}$$

To maintain consistency with the Drift–Zeeman notation, we write the orbital magnetic matrix element $\mathcal{N}^{(n)}(\mathbf{B},\mathbf{k})$ as

$$\mathcal{N}^{(n)}(\mathbf{B},\mathbf{k}) \equiv \langle n|H^{\mathrm{orb}}_{B,k}|u\rangle = \sum_{jm} \mathcal{N}^{(n)}_{jm}\, B_j k_m. \tag{71}$$

From the microscopic form of $H^{\mathrm{orb}}_{B,k}$, it follows that

$$\mathcal{N}^{(n)}_{jm} = -\frac{e\hbar}{2m}\sum_{\ell}\epsilon_{j\ell m} D^{(n)}_{\ell}, \tag{72}$$

where $D^{(n)}_{\ell}$ is the interband dipole matrix element associated with the orbital channel. The projected Drift–Orbit term is then given by

$$\delta H^{\mathrm{DO}}_{\mathrm{eff}} = \sum_{n\in Q}\frac{1}{\Delta_n}\Pi^{(n)}\mathcal{N}^{(n)}(\mathbf{B},\mathbf{k})\,\mathbb{I}_2, \tag{73}$$

which can be rewritten in terms of a third-order tensor κ_{ijm} as

$$\delta H^{\mathrm{DO}}_{\mathrm{eff}} = \sum_{ijm}\kappa_{ijm}\,\Pi_i B_j k_m\,\mathbb{I}_2, \tag{74}$$

with the tensor components defined as

$$\kappa_{ijm} = \sum_{n\in Q}\frac{1}{\Delta_n}\,\mathcal{D}^{(n)}_i\,\mathcal{N}^{(n)}_{jm}. \tag{75}$$

Substituting Eq. (72) into Eq. (75) yields the more explicit form

$$\kappa_{ijm} = -\frac{e\hbar}{2m}\sum_{n\in Q}\frac{1}{\Delta_n}\mathcal{D}^{(n)}_i\sum_{\ell}\epsilon_{j\ell m}\,D^{(n)}_{\ell}. \tag{76}$$

As this channel involves no Pauli matrices, the Drift–Orbit sector is purely scalar in spin space. In the low-energy theory, the tensor κ_{ijm} maps onto the scalar invariant

$$\delta H^{\mathrm{DO}}_{\mathrm{eff}} = \kappa\,(\mathbf{\Pi}\times\mathbf{B})\cdot\mathbf{k}\,\mathbb{I}_2, \tag{77}$$

whose explicit form for the symmetry group of tellurium is derived in the following subsection.

2.2.4 Field-induced spin-scalar and *k*-dependent sectors

We next evaluate all remaining mixed channels that generate spin-scalar terms or **k**-dependent magnetoelectric corrections.

Spin-dependent $\boldsymbol{k}$-linear correction to the Weyl spin texture

A distinct symmetry class is generated by the channel

$$\delta H_{\text{eff}}^{(\zeta)} = \frac{1}{2} P \Big([S_{\text{inv}}, H_{B,k}^{\text{orb}}] + [S_{B,k}^{\text{orb}}, H_{\text{inv}}] \Big) P. \tag{78}$$

This channel is spin dependent due to H_{inv} and linear in $\mathbf{k}$ due to $H_{B,k}^{\text{orb}}$. Using the same inversion-breaking spin-orbit matrix element as in the Drift-Zeeman sector, together with the orbital magnetic matrix element $\mathcal{N}^{(n)}(\mathbf{B}, \mathbf{k})$, we obtain

$$\delta H_{\text{eff}}^{(\zeta)} = \sum_{n \in Q} \frac{1}{\Delta_n} \, i \left(\boldsymbol{\mathcal{P}}^{(n)} \cdot \boldsymbol{\sigma} \right) \mathcal{N}^{(n)}(\mathbf{B}, \mathbf{k}). \tag{79}$$

Since $\mathcal{N}^{(n)}(\mathbf{B}, \mathbf{k})$ is scalar in spin space, the projected operator remains linear in the Pauli matrices and therefore has the general tensor form

$$\delta H_{\text{eff}}^{(\zeta)} = \sum_{kijm} \zeta_{kijm} \, \Pi_i B_j k_m \, \sigma_k, \tag{80}$$

the fourth-rank tensor ζ_{kijm} is defined as

$$\zeta_{kijm} = \sum_{n \in Q} \frac{1}{\Delta_n} \, C_{ki}^{(n)} \, \mathcal{N}_{jm}^{(n)}. \tag{81}$$

Substituting Eq. (72) into Eq. (81) yields

$$\zeta_{kijm} = -\frac{e\hbar}{2m} \sum_{n \in Q} \frac{1}{\Delta_n} \, C_{ki}^{(n)} \sum_{\ell} \epsilon_{j\ell m} \, D_{\ell}^{(n)}. \tag{82}$$

Thus, ζ_{kijm} is the tensor controlling the field-induced, spin-dependent renormalization of the Weyl spin texture. In terms of the effective couplings, this corresponds to a shift in the Weyl tensor β_{mk}, namely

$$\beta_{mk} \;\rightarrow\; \beta_{mk} + \delta\beta_{mk}(\boldsymbol{\Pi}, \mathbf{B}), \qquad \delta\beta_{mk} = \sum_{ij} \zeta_{kijm} \, \Pi_i B_j. \tag{83}$$

Scalar $\boldsymbol{k}$-independent shift

Finally, we consider the channel

$$\delta H_{\text{eff}}^{(\chi)} = \frac{1}{2} P \Big([S_{\Pi}, H_{B,p}^{\text{orb}}] + [S_{B,p}^{\text{orb}}, H_{\Pi}] \Big) P \tag{84}$$

which generates a scalar, $\mathbf{k}$-independent term. Adopting the established notation, we use the scalar polar matrix element $\Pi^{(n)}$ together with the scalar orbital magnetic

matrix element $\mathcal{O}^{(n)}(\mathbf{B})$ associated with the $\{\mathbf{A}, \mathbf{p}\}$ component of H_B^{orb}. The projected contribution reads

$$\delta H_{\mathrm{eff}}^{(\chi)} = \sum_{n \in Q} \frac{1}{\Delta_n} \Pi^{(n)} \mathcal{O}^{(n)}(\mathbf{B}) \, \mathbb{I}_2, \tag{85}$$

or, equivalently,

$$\delta H_{\mathrm{eff}}^{(\chi)} = \sum_{ij} \chi_{ij} \, \Pi_i B_j \, \mathbb{I}_2, \tag{86}$$

where the tensor χ_{ij} is defined by

$$\chi_{ij} = \sum_{n \in Q} \frac{1}{\Delta_n} \mathcal{D}_i^{(n)} \, \mathcal{O}_j^{(n)}. \tag{87}$$

This term represents a field-dependent shift in the overall energy of the conduction bands. Since it is both spin scalar and momentum independent, it does not contribute to band splitting or the effective mass. Consequently, it can be absorbed into the definition of the band origin and is omitted from the minimal effective Hamiltonian.

2.3 Symmetry reduction near the $\boldsymbol{H}$-point

The multiband downfolding procedure above yields general tensors β_{ij}, γ_{kij}, κ_{ijm}, ζ_{kijm}, and χ_{ij} appearing in the projected, low-energy Hamiltonian

$$\begin{aligned} H_{\mathrm{eff}}^{(3D)}(\mathbf{k}) = \varepsilon_0(\mathbf{k}) \, \mathbb{I}_2 + \beta_{ij} k_i \sigma_j + \gamma_{kij} \Pi_i B_j \sigma_k + \kappa_{ijm} \Pi_i B_j k_m \, \mathbb{I}_2 \\ + \zeta_{kijm} \Pi_i B_j k_m \sigma_k + \chi_{ij} \Pi_i B_j \, \mathbb{I}_2 + \ldots \end{aligned} \tag{88}$$

and repeated indices are summed over. Their symmetry-reduced form is determined by the little group (i.e., the subgroup of crystal symmetries that leaves the valley momentum invariant) near the H-point in bulk tellurium [1–4]. In the leading continuum description, the helical z-axis is distinct, while the transverse x and y directions are equivalent. In addition, time-reversal symmetry relates the two partner valleys.

As a consequence, and anticipated in Sec. 2.2.1, the zero-field Weyl tensor reduces to

$$\beta_{ij} = \mathrm{diag}(\lambda_\perp, \lambda_\perp, \lambda_z). \tag{89}$$

For the generalized Drift–Zeeman sector, the leading reduced form is

$$\delta H_{\mathrm{eff}}^{\mathrm{DZ}} \equiv \gamma_{kij} \Pi_i B_j \sigma_k = \gamma_\perp \Pi_x B_z \, \sigma_y - \gamma_z \Pi_x B_y \, \sigma_z, \tag{90}$$

or, equivalently,

$$\delta H_{\mathrm{eff}}^{\mathrm{DZ}} = -\boldsymbol{\gamma} \cdot (\boldsymbol{\Pi} \times \mathbf{B}) \cdot \boldsymbol{\sigma}, \tag{91}$$

where $\boldsymbol{\gamma} = (\gamma_\perp, \gamma_\perp, \gamma_z)$ denotes the anisotropic coupling vector acting on the components of $\boldsymbol{\Pi} \times \mathbf{B}$. For the generalized Drift–Orbit sector, the leading scalar invariant is

$$\delta H_{\mathrm{eff}}^{\mathrm{DO}} \equiv \kappa_{ijm} \Pi_i B_j k_m \, \mathbb{I}_2 = \kappa \, (\boldsymbol{\Pi} \times \mathbf{B}) \cdot \mathbf{k} \, \mathbb{I}_2. \tag{92}$$

For the k-linear spin-dependent correction, the reduced structure is written schematically as

$$\delta H_{\text{eff}}^{(\zeta)} \equiv \zeta_{kijm}\Pi_i B_j k_m \sigma_k = \zeta_\perp \Big[(\boldsymbol{\Pi}\times\mathbf{B})_x k_x\sigma_x + (\boldsymbol{\Pi}\times\mathbf{B})_y k_y\sigma_y\Big] + \zeta_z(\boldsymbol{\Pi}\times\mathbf{B})_z k_z\sigma_z + \cdots , \tag{93}$$

where the dots denotes subleading lower-symmetry anisotropies. The scalar shift $\delta H_{\text{eff}}^{(\chi)}$ remains proportional to the identity and is omitted as well in the effective theory. The general picture is summarized in the Table below.

Tensor	General structure	Reduced form in bulk Te	Meaning
β_{ij}	$\beta_{ij}k_i\sigma_j$	$\lambda_\perp(k_x\sigma_x + k_y\sigma_y) + \lambda_z k_z\sigma_z$	zero-field Weyl SOC
γ_{kij}	$\gamma_{kij}\Pi_i B_j\sigma_k$	$\gamma_\perp\Pi_x B_z\sigma_y - \gamma_z\Pi_x B_y\sigma_z$	Drift–Zeeman splitting
κ_{ijm}	$\kappa_{ijm}\Pi_i B_j k_m\,\mathbb{I}_2$	$\kappa(\boldsymbol{\Pi}\times\mathbf{B})\cdot\mathbf{k}\,\mathbb{I}_2$	Drift–Orbit shift
ζ_{kijm}	$\zeta_{kijm}\Pi_i B_j k_m\sigma_k$	correction to Weyl SOC	renormalization of $\lambda_\perp, \lambda_z$
χ_{ij}	$\chi_{ij}\Pi_i B_j\,\mathbb{I}_2$	scalar shift	band-origin shift (ignored)

In the effective Hamiltonian used in the main text, we retain the dominant zero-field Weyl term together with the leading Drift–Zeeman and Drift–Orbit interactions,

$$H_{\text{eff}}^{\text{Te}} \simeq \varepsilon_0(\mathbf{k})\,\mathbb{I}_2 + \lambda_\perp(k_x\sigma_x + k_y\sigma_y) + \lambda_z k_z\sigma_z - \boldsymbol{\gamma}\cdot(\boldsymbol{\mathcal{E}}\times\mathbf{B})\cdot\boldsymbol{\sigma} + \kappa(\boldsymbol{\mathcal{E}}\times\mathbf{B})\cdot\mathbf{k}\,\mathbb{I}_2. \tag{94}$$

Here $\boldsymbol{\mathcal{E}}$ is introduced only as a compact low-energy parameterization of the inversion-breaking polar strength associated with $\boldsymbol{\Pi}$, with $\boldsymbol{\Pi} \propto \boldsymbol{\mathcal{E}}$. The field-induced correction $\delta H_{\text{eff}}^{(\zeta)}$ is first order in $\boldsymbol{\Pi}\times\mathbf{B}$ and therefore renormalizes the Weyl spin-orbit coefficients only weakly compared with the dominant bare values $\lambda_\perp, \lambda_z$.

2.4 Pure polar and pure magnetic linear terms

For completeness, we briefly examine the terms that are linear only in the inversion-breaking polar strength $\boldsymbol{\Pi}$ or only in the magnetic field $\mathbf{B}$, comparing them with the mixed magnetoelectric terms derived above.

Order $\boldsymbol{\Pi}$

A term linear only in $\boldsymbol{\Pi}$ arise from the direct projection of the scalar inversion-breaking operator,

$$\delta H_{\text{eff}}^{(\Pi)} = P H_\Pi P = \sum_i \alpha_i \Pi_i\,\mathbb{I}_2, \tag{95}$$

with coefficients α_i determined by the projected matrix elements of $V_{\text{odd}}(\mathbf{r})$. Since this term is scalar in spin space and independent of momentum, it does not split the Kramers doublet and can be absorbed into a redefinition of the band origin $\varepsilon_0(\mathbf{k})$. In the bulk high-symmetry description of tellurium, no additional invariant can be formed at linear order in $\boldsymbol{\Pi}$ beyond such a trivial scalar contribution. Accordingly, we omit Eq. (95) from the effective Hamiltonian.

Order $\boldsymbol{B}$

The situation differs for terms linear only in the magnetic field. In the pure magnetic sector, the leading spin-dependent contribution is the projected Zeeman term

$$\delta H_{\mathrm{eff}}^{(B)} = \mu_B \sum_{ij} g_{ij} B_i \sigma_j, \tag{96}$$

where μ_B is the Bohr magneton and g_{ij} is the effective multiband g-tensor of the conduction doublet. This tensor deviates from the bare free-electron value ($g_e \approx 2$), as it incorporates the results of the Löwdin downfolding procedure applied to the bare spin coupling together with remote-band orbital and spin-orbit admixture. In semiconductors, multiband $k\cdot p$ mixing with remote orbital and spin–orbit-coupled bands often renormalize the bare Zeeman coupling, $H_Z = -g_e \mu_B \mathbf{B}\cdot\sigma/2$, and, in some cases, nearly cancel it [5, 6]. Within the present framework, this provides a natural interpretation of the experimentally observed negligibly small effective g-factor in tellurene: the ordinary Zeeman sector is not absent microscopically, but its renormalization in the projected conduction doublet renders it subdominant. This leaves the polar-mediated Drift–Zeeman interaction as the dominant experimentally relevant field-induced spin splitting. Since our goal is to identify the mechanism that drives the transport anomalies, and considering that the ordinary Zeeman sector is experimentally negligible for the relevant conduction band, we omit Eq. (96) from our minimal effective Hamiltonian and retain instead the Drift–Zeeman and Drift–Orbit terms derived above.

2.5 Absence of spin splitting from the Drift–Orbit term

The Drift–Orbit term in Eq. (77) is proportional to the identity matrix in spin space. Consequently, it shifts the dispersion of the two spin branches equally and does not contribute to their splitting,

$$\varepsilon(\mathbf{k}) \to \varepsilon(\mathbf{k}) + \kappa(\boldsymbol{\mathcal{E}} \times \mathbf{B})\cdot\mathbf{k}, \tag{97}$$

which corresponds to a uniform shift of the group velocity,

$$\mathbf{v} = \frac{1}{\hbar}\nabla_{\mathbf{k}}\varepsilon(\mathbf{k}) \to \mathbf{v} + \frac{\kappa}{\hbar}(\boldsymbol{\mathcal{E}} \times \mathbf{B}). \tag{98}$$

By contrast, the Drift–Zeeman term is spin dependent and acts with opposite signs on the two branches. It is therefore the term responsible for the field-induced band splitting entering the quantum-geometric diffusion mechanism discussed in the main text.

2.6 Band splitting induced by the magnetoelectric coupling

Having established the multiband origin of the projected magnetoelectric terms, we now specialize the theory to the effective transport Hamiltonian relevant to the experimental devices. Although the microscopic parent theory is three-dimensional, the

transport regime probed in the present tellurene films is quasi-2D, as the motion along the $\hat{y}$ direction, perpendicular to the x–z plane of the film, is confined by the finite sample thickness.

We therefore begin with the effective 3D Weyl-like Hamiltonian for the conduction-band Kramers doublet,

$$H_0^{(3D)} = \frac{\hbar^2(k_x^2 + k_y^2)}{2m_\perp^*} + \frac{\hbar^2 k_z^2}{2m_z^*} + \lambda_\perp(k_x\sigma_x + k_y\sigma_y) + \lambda_z k_z \sigma_z, \tag{99}$$

where $m_\perp^*$ and m_z^* are the effective masses perpendicular and parallel to the helical axis, respectively, and $\lambda_\perp, \lambda_z$ are the anisotropic spin-orbit couplings inherited from the bulk crystal.

To incorporate the spatial confinement, we introduce a potential $V_{\text{conf}}(y)$ that restricts the electronic motion to a slab of thickness L along $\hat{y}$. Within the envelope-function approximation, the wavefunction is factorized as $\Psi(x, y, z) = \psi(x, z)\phi_n(y)$, where $\psi(x, z)$ is a two-component spinor describing the in-plane motion and $\phi_n(y)$ is the envelope wavefunction of the n-th confinement subband. Given that Shubnikov–de Haas oscillations indicate the occupation of only the lowest subband ($n = 1$) in the relevant gate-voltage regime, the reduced quasi-2D Hamiltonian is obtained from

$$H_0^{(2D)} = \langle \phi_1 | H_0^{(3D)} | \phi_1 \rangle_y, \tag{100}$$

where the subscript y indicates integration over the confined dimension, with the operator $k_y \to -i\partial_y$. Let us now evaluate the various terms in Eq. (99) under this projection.

First, the terms linear in k_y vanish identically in the lowest bound state

$$\langle \phi_1 | k_y | \phi_1 \rangle = \int dy\, \phi_1^*(y)\, (-i\partial_y)\phi_1(y) = 0. \tag{101}$$

This follows from the fact that $\phi_1(y)$ can be chosen real for a bound state of the confinement potential, in which case the integrand reduces to a total derivative $\partial_y(\phi_1^2)$ whose boundary contribution vanishes. Equivalently, for a stationary bound state one has no net current flow along the confined direction. As a consequence, the projected spin-orbit term $\lambda_\perp k_y \sigma_y$ drops out of the effective low-energy Hamiltonian.

Second, the quadratic term in k_y contributes only a constant confinement energy,

$$\frac{\hbar^2}{2m_\perp^*} \langle \phi_1 | k_y^2 | \phi_1 \rangle \equiv E_{\text{conf}}, \tag{102}$$

which depends on the details of the confinement potential and on the slab thickness L, but is independent of the in-plane momenta (k_x, k_z). This term therefore produces only a rigid shift of the subband bottom and does not affect the in-plane dispersion, the spin splitting, or the quantum metric. Since only energy differences within the projected conduction doublet are relevant in what follows, we absorb this constant into the chemical potential and drop it from the effective Hamiltonian.

The resulting projected quasi-2D Hamiltonian becomes

$$H_0^{(2\mathrm{D})} = \frac{\hbar^2 k_x^2}{2m_\perp^*} + \frac{\hbar^2 k_z^2}{2m_z^*} + \lambda_\perp k_x \sigma_x + \lambda_z k_z \sigma_z. \tag{103}$$

This shows that the confined transport in tellurene films is governed by a 2D Weyl-like structure, restricted to the in-plane momenta (k_x, k_z), while retaining the anisotropic spin-orbit properties of the bulk crystal.

We now add the spin-dependent magnetoelectric term derived in the previous subsection. The scalar Drift–Orbit contribution is omitted here because it does not produce spin splitting. The effective transport Hamiltonian therefore takes the form

$$H^{(2D)} = \frac{\hbar^2 k_x^2}{2m_\perp^*} + \frac{\hbar^2 k_z^2}{2m_z^*} + \lambda_\perp k_x \sigma_x + \lambda_z k_z \sigma_z - \boldsymbol{\gamma} \cdot (\boldsymbol{\mathcal{E}} \times \mathbf{B}) \cdot \boldsymbol{\sigma}, \tag{104}$$

where the vector $\boldsymbol{\mathcal{E}}$ is used only as a compact low-energy parameterization of the inversion-breaking polar strength. The Hamiltonian can be written as

$$H^{(2D)} = H_0 \mathbb{I}_2 + \mathbf{d} \cdot \boldsymbol{\sigma}, \tag{105}$$

with

$$H_0 = \frac{\hbar^2 k_x^2}{2m_\perp^*} + \frac{\hbar^2 k_z^2}{2m_z^*}, \qquad \mathbf{d} = (\lambda_\perp k_x, 0, \lambda_z k_z) - \boldsymbol{\gamma} \cdot (\boldsymbol{\mathcal{E}} \times \mathbf{B}). \tag{106}$$

Its eigenvalues are

$$E_\pm = H_0 \pm |\mathbf{d}|, \tag{107}$$

so that the band splitting is

$$\Delta E = E_+ - E_- = 2|\mathbf{d}|. \tag{108}$$

Therefore

$$(\Delta E)^2 = 4(d_x^2 + d_y^2 + d_z^2). \tag{109}$$

Explicitly,

$$\begin{aligned}(\Delta E)^2 = 4\Big[&(\lambda_\perp k_x - \gamma_\perp(\mathcal{E}_y B_z - \mathcal{E}_z B_y))^2 + (\gamma_\perp(\mathcal{E}_z B_x - \mathcal{E}_x B_z))^2 \\ &+ (\lambda_z k_z - \gamma_z(\mathcal{E}_x B_y - \mathcal{E}_y B_x))^2\Big].\end{aligned} \tag{110}$$

For the confined quasi-2D transport regime relevant here, and for an inversion-breaking polar texture parameterized by

$$\boldsymbol{\mathcal{E}} \approx (\mathcal{E}_x, 0, 0), \tag{111}$$

one obtains

$$(\Delta E)^2 = 4\left[\lambda_\perp^2 k_x^2 + \lambda_z^2 k_z^2 - 2\gamma_z \mathcal{E}_x \lambda_z k_z B_y + \mathcal{E}_x^2(\gamma_z^2 B_y^2 + \gamma_\perp^2 B_z^2)\right]. \tag{112}$$

This is the field-dependent splitting that enters the quantum-geometric diffusion mechanism discussed in the main text.

For later reference, the zero-field quantum-metric components associated with the quasi-2D Hamiltonian (103) are

$$\begin{aligned} g_{zz}(\mathbf{k}) &= \frac{\lambda_z^2 \lambda_\perp^2 k_x^2}{4\left[k_x^2\lambda_\perp^2 + k_z^2\lambda_z^2\right]^2}, \\ g_{zx}(\mathbf{k}) &= -\frac{\lambda_z^2 \lambda_\perp^2 k_x k_z}{4\left[k_x^2\lambda_\perp^2 + k_z^2\lambda_z^2\right]^2}. \end{aligned} \tag{113}$$

These expressions are the ones used in the low-field expansion of the geometric diffusion and in the analysis of the symmetric magnetoresistance coefficient.

3 Low-field expansion and quantum metric averages

In this Section we provide a formal justification for evaluating the quantum-geometric diffusion using the zero-field quantum metric. The argument relies on the symmetry properties of the Fermi-surface (FS) averages combined with a controlled Taylor expansion of the field-dependent metric. We demonstrate explicitly that, in the semiclassical regime $B \ll B^*$, where B^* is a characteristic field to be determined, (i) the symmetric contribution to the magnetoresistance (MR) is entirely determined by the $B = 0$ metric up to order B^2, and (ii) no intrinsic antisymmetric (odd-in-B) contribution arises from quantum geometry at leading order. Throughout this section, the FS average is understood as a quasi-2D average over the confined k_x–k_z Fermi contour relevant to the lowest occupied subband.

3.1 Field dependence of the quantum metric

In the presence of an electric field $\boldsymbol{\mathcal{E}} \parallel \hat{x}$ and a magnetic field $\mathbf{B} \parallel \hat{y}$, the quantum metric entering the geometric diffusion takes the form

$$g_{zz}(\mathbf{k}, \mathbf{B}) = \frac{\lambda_z^2 \lambda_\perp^2 k_x^2}{4\left[k_x^2\lambda_\perp^2 + (k_z\lambda_z + \gamma_z \mathcal{E}_x B_y)^2\right]^2}. \tag{114}$$

This expression can be rewritten more compactly as a shift in the momentum argument of the zero-field metric

$$g_{zz}(\mathbf{k}, B) = g_{zz}^{(0)}(k_x, k_z + \delta)\,, \qquad \delta \equiv \frac{\gamma_z}{\lambda_z}\,\mathcal{E}_x B, \tag{115}$$

where $g_{zz}^{(0)}$ is the zero-field metric, an even function of k_z, and δ represents the drift-induced momentum shift. For sufficiently small fields, $B \ll B^*$, the metric can be expanded as

$$g_{zz}(\mathbf{k}, B) = g_{zz}^{(0)} + \delta\,\partial_{k_z} g_{zz}^{(0)} + \frac{\delta^2}{2}\,\partial_{k_z}^2 g_{zz}^{(0)} + \mathcal{O}(\delta^3). \tag{116}$$

Since $g_{zz}^{(0)}$ is even in k_z, its first derivative is odd, whereas the second derivative is even.

3.2 Symmetric contribution

The symmetric geometric correction to the longitudinal diffusion involves the FS average

$$\langle g_{zz}(\mathbf{k}, B)\rangle_{\mathrm{FS}}\,(\boldsymbol{\mathcal{E}}\times\mathbf{B})^2 \propto \langle g_{zz}(\mathbf{k}, B)\rangle_{\mathrm{FS}}\,B^2. \tag{117}$$

Substituting Eq. (116) and invoking the symmetry of the FS under $k_z \to -k_z$, we obtain

$$\begin{aligned}\langle g_{zz}(\mathbf{k}, B)\rangle_{\mathrm{FS}} &= \langle g_{zz}^{(0)}\rangle_{\mathrm{FS}} + \delta\,\langle \partial_{k_z} g_{zz}^{(0)}\rangle_{\mathrm{FS}} + \frac{\delta^2}{2}\,\langle \partial_{k_z}^2 g_{zz}^{(0)}\rangle_{\mathrm{FS}} + \cdots \\ &= \langle g_{zz}^{(0)}\rangle_{\mathrm{FS}} + \mathcal{O}(\delta^2),\end{aligned} \tag{118}$$

where the linear term vanishes because $\partial_{k_z} g_{zz}^{(0)}$ is odd in k_z, resulting in a vanishing FS average. Given that $\delta \propto B$, the leading correction to $\langle g_{zz}\rangle_{\mathrm{FS}}$ appears at order B^2. Therefore, the contribution becomes

$$\langle g_{zz}(\mathbf{k}, B)\rangle_{\mathrm{FS}}\,B^2 = \langle g_{zz}^{(0)}\rangle_{\mathrm{FS}}\,B^2 + \mathcal{O}(B^4). \tag{119}$$

This result demonstrates that the coefficient C of the quadratic NMR is entirely controlled by the zero-field quantum metric. The explicit field dependence of g_{zz} produces only higher-order (B^4) corrections and is therefore negligible in the low-field regime.

3.3 Would-be antisymmetric contribution

The geometric diffusion formally allows, in principle, for a term of the form

$$\langle k_z g_{zz}(\mathbf{k}, B)\rangle_{\mathrm{FS}}\,\hat{\mathbf{z}}\cdot(\boldsymbol{\mathcal{E}}\times\mathbf{B}) \;\propto\; \langle k_z g_{zz}(\mathbf{k}, B)\rangle_{\mathrm{FS}}\,B. \tag{120}$$

We now show that this structure does not generate an intrinsic odd-in-B contribution at leading order. Inserting the expansion (116), we obtain

$$\langle k_z g_{zz}(\mathbf{k}, B)\rangle_{\mathrm{FS}} = \langle k_z g_{zz}^{(0)}\rangle_{\mathrm{FS}} + \delta\,\langle k_z\partial_{k_z} g_{zz}^{(0)}\rangle_{\mathrm{FS}} + \frac{\delta^2}{2}\,\langle k_z\partial_{k_z}^2 g_{zz}^{(0)}\rangle_{\mathrm{FS}} + \cdots . \tag{121}$$

Both the first and third terms vanish by symmetry, as $k_z g_{zz}^{(0)}$ and $k_z\partial_{k_z}^2 g_{zz}^{(0)}$ are odd functions of k_z, resulting in vanishing FS averages. The leading nonzero contribution arises from the second term. In this case, both k_z and $\partial_{k_z} g_{zz}^{(0)}$ are odd, so their product is even. Hence,

$$\langle k_z g_{zz}(\mathbf{k}, B)\rangle_{\mathrm{FS}} = \delta\,\langle k_z\partial_{k_z} g_{zz}^{(0)}\rangle_{\mathrm{FS}} + \mathcal{O}(\delta^3) \propto B. \tag{122}$$

Multiplying by the explicit factor of B in Eq. (120), the resulting contribution to the diffusion, and thus to the resistance, scales as B^2 and is therefore even in the magnetic field. Consequently, within the semiclassical expansion controlled by $B \ll$

B^*, the quantum geometry does not produce an intrinsic antisymmetric (odd-in-B) longitudinal MR.

The Taylor expansion above establishes two key results. First, the quadratic NMR coefficient C is determined entirely by the zero-field quantum metric, as corrections arising from the field dependence of g_{zz} appear only at order B^4. Second, although the geometric diffusion formally contains a term involving $\langle k_z g_{zz}\rangle_{\mathrm{FS}}$, its leading contribution is even in B and therefore does not generate an intrinsic antisymmetric MR. These results justify utilizing the $B = 0$ quantum metric in the analysis of the low-field data. Furthermore, they support the interpretation of any residual odd-in-B component as extrinsic in origin.

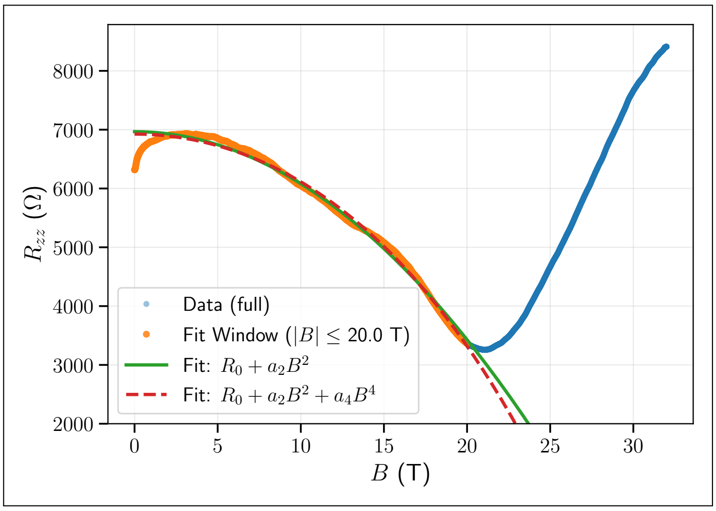

Fig. S1: **Field dependence in the quantum metric.** Fit of the giant negative magnetoresistance (GNMR) for $V_{bg} = 16$V and $V_{tg} = -4$V as a function of the magnetic field $\mathbf{B}$ using both quadratic, B^2, and quartic, B^4, powers in the expansion of $g_{zz}(\mathbf{B})$. As we can see, below the crossover field $B^* \sim \lambda_z k_F/\gamma_z\mathcal{E}_x$ the behavior corresponds to an exact parabolic drop of resistance, as obtained from the $g_{zz}(0)$ limit.

3.4 Regime of validity for the expansion – small field $B \ll B^*$

The magnetic-field dependence of the quantum metric enters through the drift-induced momentum shift $\lambda_z k_z \to \lambda_z k_z + \gamma_z\mathcal{E}_x B$ in the denominator of $g_{zz}(\mathbf{k}, B)$. Consequently, a natural crossover (or saturation) field scale B^* is established by the condition that the drift shift becomes comparable to the typical k_z scale on the Fermi surface. This condition can be written as

$$\gamma_z\mathcal{E}_x B^* \sim \lambda_z k_F \qquad \Rightarrow \qquad B^* \sim \frac{\lambda_z k_F}{\gamma_z \mathcal{E}_x}. \tag{123}$$

Using the microscopic estimate for the Drift–Zeeman coefficient, $\gamma_z \simeq 2e^2\lambda_z^3/(\hbar\Delta^3)$, we obtain

$$B^* \sim \frac{\hbar\Delta^3}{2e^2\lambda_z^2}\,\frac{k_F}{\mathcal{E}_x}. \tag{124}$$

To provide a quantitative estimate its magnitude, we use representative parameters for tellurene. Assuming $\lambda_z \simeq 10^{-9}\,\mathrm{eV\,cm}$, $k_F \simeq 10^6\,\mathrm{cm}^{-1}$, and a conservative bandwidth scale $\Delta = 50$ meV, Eq. (124) gives the estimate

$$B^* \approx \frac{4.1\times 10^{10}}{\mathcal{E}_x\,[\mathrm{V/m}]}\ \mathrm{T}. \tag{125}$$

For an internal effective electric field of order $\mathcal{E}_x \sim 10^9$ V/m (a GV/m scale is natural for microscopic polar or lone-pair fields even after screening), this yields $B^* \sim 40-50$ T. This value is already at least twice larger than the magnetic field scale (~ 20 T) where the experimental curves exhibit a minimum. Moreover, any smaller effective field (e.g. due to stronger screening of the internal polarization field) increases B^* further.

Therefore, throughout the fitting window used in the main analysis (up to ~ 20 T), the condition $B \ll B^*$ is well satisfied. In this regime it is justified to evaluate the FS averages of the quantum metric using its $B = 0$ form. The leading corrections from the field dependence of g_{zz} enter only at higher order in B.

3.5 Fermi surface averages

It is important to note that in the presence of an external magnetic field, the FS is formally defined by the field-dependent dispersion $\varepsilon(\mathbf{k},\mathbf{B}) = \mu$. In what follows, we demonstrate that even when accounting for this field-dependent manifold, the symmetry properties used to obtain the FS averages in the low-field expansion remain invariant. To this end, the FS averages must be defined over the modified manifold FS_B as

$$\langle \mathcal{O}\rangle_{FS_B}(B) \equiv \frac{\int \frac{d^3k}{(2\pi)^3}\,\mathcal{O}(\mathbf{k};B)\,\delta[\varepsilon(\mathbf{k};B)-\mu]}{\int \frac{d^3k}{(2\pi)^3}\,\delta[\varepsilon(\mathbf{k};B)-\mu]}\,. \tag{126}$$

However, in the geometry relevant here ($\boldsymbol{\mathcal{E}} \parallel \hat{x}$ and $\mathbf{B} \parallel \hat{y}$), the magnetic field enters the semiclassical dispersion through a time-reversal-even combination, i.e. $(\boldsymbol{\mathcal{E}}\times\mathbf{B})^2 \propto B^2$, inside the square-root structure of the two-band energies. Consequently, the field-induced energy shift of the band dispersion, $\delta\varepsilon(\mathbf{k};B) \equiv \varepsilon(\mathbf{k};B) - \varepsilon(\mathbf{k};0)$, is an even function of B to all orders in a Taylor expansion around $B = 0$

$$\delta\varepsilon(\mathbf{k};B) = c_2(\mathbf{k})\,B^2 + c_4(\mathbf{k})\,B^4 + \cdots\,. \tag{127}$$

This immediately implies that the FS shift does not generate any odd-in-B correction to longitudinal (time-reversal-even) transport coefficients in the perturbative regime.

To see this explicitly, consider the expansion of the δ-function in (126),

$$\delta[\varepsilon(\mathbf{k};B)-\mu] = \delta[\varepsilon_0(\mathbf{k}) - \mu + \delta\varepsilon(\mathbf{k};B)]$$

$$= \delta(\varepsilon_0 - \mu) - \delta\varepsilon(\mathbf{k}; B)\, \delta'(\varepsilon_0 - \mu) + \frac{1}{2}\big[\delta\varepsilon(\mathbf{k}; B)\big]^2\, \delta''(\varepsilon_0 - \mu) + \cdots . \tag{128}$$

Because $\delta\varepsilon(\mathbf{k}; B)$ is even in B [Eq. (127)], every correction generated by this expansion respects this property. The only effect of using $\varepsilon(\mathbf{k}; B)$ in the FS average is to renormalize the coefficients of the even-power MR series (e.g., the prefactor of the leading B^2 term and the subleading B^4 correction), but it cannot generate new odd-in-B structures.

A completely analogous statement applies to the quantity $\langle k_z\, g_{zz}^{(0)}(\mathbf{k})\rangle_{FS} = 0$ at $B = 0$. At zero magnetic field, the FS measure $\delta(\varepsilon_0 - \mu)$ is even under $k_z \to -k_z$, while k_z itself is odd. Hence, any integrand of the form $k_z \times$ (even function of k_z) averages to zero over the FS. When the field dependence of the quantum metric is included, $g_{zz}(\mathbf{k}; B)$ can be expanded as

$$g_{zz}(\mathbf{k}; B) = g_{zz}^{(0)}(\mathbf{k}) + g_{zz}^{(2)}(\mathbf{k})\, B^2 + g_{zz}^{(4)}(\mathbf{k})\, B^4 + \cdots , \tag{129}$$

because the only physically relevant field dependence, in the present geometry, enters through $(\boldsymbol{\mathcal{E}} \times \mathbf{B})^2 \propto B^2$ at the level of the effective two-band problem. Replacing (129) into the FS average defined as (126) namely, $\langle k_z g_{zz}\rangle_{FS_B}$ yields terms of the form $\langle k_z\, g_{zz}^{(2m)}(\mathbf{k})\rangle_{FS}\, B^{2m}$. Each term vanishes whenever $g_{zz}^{(2m)}(\mathbf{k})$ remains even under $k_z \to -k_z$ with respect to the same zero-field FS measure, exactly the symmetry structure previously used. Corrections arising from FS shifting, generated by (128), are also even in B and therefore cannot produce a genuine odd-in-B contribution to the longitudinal MR. Their only effect is to renormalize the even-power coefficients already present.

4 Fitting Formulae

4.1 Angular dependence

We begin from the Kubo-Greenwood formula for the conductivity tensor in an n-band system

$$\sigma_{ij} = e^2 \sum_n \int \frac{d^d\mathbf{k}}{(2\pi)^d} D_{ij}^{(n)}(\mathbf{k}) \left(-\frac{\partial f(\epsilon_n(\mathbf{k}))}{\partial \epsilon_n(\mathbf{k})} \right), \tag{130}$$

where $D_{ij}^{(n)}(\mathbf{k})$ is the diffusion tensor. At low temperatures, $-\partial f/\partial\epsilon \approx \delta(\epsilon - \mu)$, which simplifies Eq. (130) to

$$\sigma_{ij} \approx e^2 N(E_F) D_{ij}(\mu), \tag{131}$$

where $N(E_F)$ is the density of states at the Fermi level. The total diffusion tensor $D_{ij}(\mu)$ consists of a conventional Drude contribution and a quantum geometric contribution,

$$D_{ij}(\mu) = \underbrace{D_{ij}^D(\mu)}_{\text{intraband}} + \underbrace{\frac{2\tau_g}{\hbar^2}\langle[\Delta\epsilon(\mathbf{k})]^2 g_{ij}(\mathbf{k})\rangle_{\text{FS}}}_{\text{interband (geometric)}}, \tag{132}$$

where τ_g is the quantum geometric relaxation time and $g_{ij}(\mathbf{k})$ is the quantum metric. For the zz-component relevant to our longitudinal resistance measurements

$$D_{zz}(\mu) = D_{zz}^{D}(\mu) + \frac{2\tau_g}{\hbar^2}\langle[\Delta\epsilon(\mathbf{k})]^2 g_{zz}(\mathbf{k})\rangle_{\mathrm{FS}}. \tag{133}$$

The FS average expands as

$$\begin{aligned}\langle[\Delta\epsilon(\mathbf{k})]^2 g_{zz}(\mathbf{k})\rangle_{\mathrm{FS}} = &+4\langle(\lambda_\perp^2 k_x^2 + \lambda_z^2 k_z^2) g_{zz}(\mathbf{k})\rangle_{\mathrm{FS}} \\ &- 8\gamma_z\langle k_z g_{zz}(\mathbf{k})\rangle_{\mathrm{FS}}\,\hat{z}\cdot(\boldsymbol{\mathcal{E}}\times\mathbf{B}) \\ &+ 4\langle g_{zz}(\mathbf{k})\rangle_{\mathrm{FS}}\,[\gamma\cdot(\boldsymbol{\mathcal{E}}\times\mathbf{B})]^2,\end{aligned} \tag{134}$$

given our experimental geometry ($\boldsymbol{\mathcal{E}} \parallel \hat{x}$, $\mathbf{B} \parallel \hat{y}$, transport measurements along $\hat{z}$)

$$\hat{z}\cdot(\boldsymbol{\mathcal{E}}\times\mathbf{B}) = \mathcal{E}_x B_y, \quad \gamma\cdot(\boldsymbol{\mathcal{E}}\times\mathbf{B}) = \gamma_z \mathcal{E}_x B_y. \tag{135}$$

4.2 Symmetric component – Intrinsic

The symmetric-in-B part of D_{zz} becomes

$$D_{zz}^{\mathrm{sym}}(B) = D_{zz}^{D} + \frac{2\tau_g}{\hbar^2}S_0 + \frac{2\tau_g}{\hbar^2}4\langle g_{zz}(\mathbf{k})\rangle_{\mathrm{FS}}\gamma_z^2\mathcal{E}_x^2 B_y^2, \tag{136}$$

where $S_0 = 4\langle(\lambda_\perp^2 k_x^2 + \lambda_z^2 k_z^2) g_{zz}(\mathbf{k})\rangle_{\mathrm{FS}}$. The conductivity is then

$$\sigma_{zz}(B) = e^2 N(E_F) D_{zz}^{\mathrm{sym}}(B) = \sigma_0 + \alpha B_y^2, \tag{137}$$

with

$$\sigma_0 = e^2 N(E_F)\left(D_{zz}^{D} + \frac{2\tau_g}{\hbar^2}S_0\right), \tag{138}$$

$$\alpha = e^2 N(E_F)\frac{8\tau_g}{\hbar^2}\langle g_{zz}(\mathbf{k})\rangle_{\mathrm{FS}}\gamma_z^2\mathcal{E}_x^2. \tag{139}$$

The resistance $R_{zz} = \frac{1}{\sigma_{zz}}\frac{L}{A}$ expands for weak magnetic fields as

$$R(B) \approx \frac{L}{A}\left[\frac{1}{\sigma_0} - \frac{\alpha}{\sigma_0^2}B_y^2 + O(B^4)\right], \tag{140}$$

yielding the fitting form

$$R(B) = R_0 - C B_y^2, \tag{141}$$

where

$$C = \frac{L}{A}\frac{\alpha}{\sigma_0^2}. \tag{142}$$

Substituting for α and σ_0

$$C = \frac{L}{A}\frac{e^2 N(E_F)\frac{8\tau_g}{\hbar^2}\langle g_{zz}\rangle_{\rm FS}\gamma_z^2\mathcal{E}_x^2}{\left[e^2 N(E_F)\left(D_{zz}^D + \frac{2\tau_g}{\hbar^2}S_0\right)\right]^2}. \tag{143}$$

Using the relationship between diffusion and conductivity, $\sigma = e^2 N(E_F)D$, we find

$$C = \frac{(L/A)e^2 N(E_F)(8\tau_g/\hbar^2)\langle g_{zz}\rangle_{\rm FS}\gamma_z^2\mathcal{E}_x^2}{\left(\sigma_{zz}^D + \sigma_{zz}^g\right)^2}, \tag{144}$$

where the $\mathbf{B}-$independent, quantum geometric conductivity $\sigma_{zz}^g = N(E_F)(2\tau_g/\hbar^2)S_0$. For the general case in which the magnetic field is rotated away from the $y-$axis,

$$C(\theta) = \frac{(L/A)\ e^2 N(E_F)\ (8\tau_g/\hbar^2)\ \langle g_{zz}(\mathbf{k})\rangle_{\rm FS}\ \mathcal{E}_x^2}{\left(\sigma_{zz}^D + \sigma_{zz}^g\right)^2}(\gamma_z^2\cos^2\theta_{(\mathbf{B},\hat{y})} + \gamma_\perp^2\cos^2\theta_{(\mathbf{B},\hat{z})}),$$

where $\theta_{(\mathbf{B},\hat{y})}$ and $\theta_{(\mathbf{B},\hat{z})}$ are, respectively, the angles between the magnetic field $\mathbf{B}$ and the $y-$ and $z-$axis. Finally, assuming $\sigma_{zz}^D \gg \sigma_{zz}^g$

$$C(\theta) \approx \frac{(L/A)\ e^2 N(E_F)\ (8\tau_g/\hbar^2)\ \langle g_{zz}\rangle_{\rm FS}\ \mathcal{E}_x^2}{(\sigma_{zz}^D)^2}(\gamma_z^2\cos^2\theta_{(\mathbf{B},\hat{y})} + \gamma_\perp^2\cos^2\theta_{(\mathbf{B},\hat{z})}). \tag{145}$$

4.3 Antisymmetric component – Extrinsic

In this Section we show that the small antisymmetric (odd-in-B) contribution observed in the measured longitudinal resistance $R_{zz}(B)$ has a purely extrinsic origin. It can be quantitatively explained by a small admixture of the Hall voltage into the longitudinal channel. Such mixing can arise from imperfect contact geometry and/or a slight misalignment of the device with respect to the magnetic field. Using the experimentally relevant values of the carrier density, mobility, and device dimensions, we demonstrate that this mechanism naturally produces an odd-in-B signal of order 1–5 Ω/T, fully consistent with the measurements.

It is convenient to decompose the measured longitudinal resistance $R_{zz}^{\rm meas}(B)$ into even and odd components under magnetic-field reversal,

$$R_{zz}^{\rm even}(B) = \frac{R_{zz}^{\rm meas}(B) + R_{zz}^{\rm meas}(-B)}{2}, \quad R_{zz}^{\rm odd}(B) = \frac{R_{zz}^{\rm meas}(B) - R_{zz}^{\rm meas}(-B)}{2}. \tag{146}$$

In a time-reversal-invariant system the intrinsic longitudinal response is even in B, whereas the Hall response is odd in B. A common source of an odd-in-B contamination in longitudinal measurements is therefore an imperfect subtraction of the Hall voltage. To leading order one may write

$$R_{zz}^{\rm meas}(B) \simeq R_{zz}^{\rm int}(B) + \eta\, R_{zy}(B), \tag{147}$$

where $\eta \ll 1$ is a dimensionless mixing factor encoding geometric imperfections such as slight voltage-probe misplacement, asymmetric current paths, finite contact size, or a small tilt of the voltage leads. Eq. (147) immediately implies $R_{zz}^{\text{odd}}(B) \simeq \eta\, R_{zy}(B)$.

For a single electron-like conduction band in a quasi-2D thin film, the Hall resistivity is

$$\rho_{zy}(B) = \frac{B}{n_{2D}e}, \tag{148}$$

where n_{2D} is the sheet carrier density. Using the experimentally relevant value $n_{2D} \sim 10^{12}$ cm^{-2} ($= 10^{16}$ m^{-2}), one obtains

$$\frac{d\rho_{zy}}{dB} = \frac{1}{n_{2D}e} \approx 6 \times 10^2\ \Omega/\text{T}. \tag{149}$$

Including the usual geometric factor $G_H \sim \mathcal{O}(1)$ that relates resistivity to the measured resistance, the Hall slope in resistance units is

$$\frac{dR_{zy}}{dB} \sim G_H \times 6 \times 10^2\ \Omega/\text{T}. \tag{150}$$

The odd longitudinal slope is therefore expected to be

$$\frac{dR_{zz}^{\text{odd}}}{dB} \simeq \eta\, \frac{dR_{zy}}{dB} \sim \eta\, G_H\, (6 \times 10^2)\ \Omega/\text{T}. \tag{151}$$

The experimentally extracted antisymmetric component corresponds to approximately 1–5 Ω/T, which requires only a sub-percent mixing factor,

$$\eta \sim (2 \times 10^{-3})\text{–}(8 \times 10^{-3}), \tag{152}$$

i.e. roughly $\eta \simeq 0.2\%$–0.8%. Such values are entirely realistic for micron-scale devices.

A simple geometric estimate can be obtained by considering a Hall bar of width W with a small transverse misplacement Δy of the voltage probes, yielding $\eta \sim \Delta y/W$. For $W \sim 5$ μm, a mixing factor $\eta \sim 0.5\%$ corresponds to a misalignment $\Delta y \sim 25$ nm, which is well within typical lithographic tolerances and finite contact dimensions. The effect becomes further enhanced at high magnetic fields, where the Hall angle increases. Using the measured mobility $\mu \sim 2000$ cm^2/Vs, one finds $\mu B \gtrsim 1$ already for $B \gtrsim 5$ T and $\mu B \sim 4$ at $B = 20$ T, implying a strong transverse response and a natural sensitivity to small Hall leakage.

Finally, for field rotations of the form $\mathbf{B} = B(\cos\theta\,\hat{y} + \sin\theta\,\hat{z})$, the antisymmetric component of the measured longitudinal resistance follows approximately a $\cos\theta$ dependence. This behavior is naturally explained by Hall admixture: since the Hall response is controlled by the perpendicular field component $B_\perp = B\cos\theta$, one finds

$$R_{zz}^{\text{AS}}(\theta, B) \approx \eta\, R_H\, B\cos\theta. \tag{153}$$

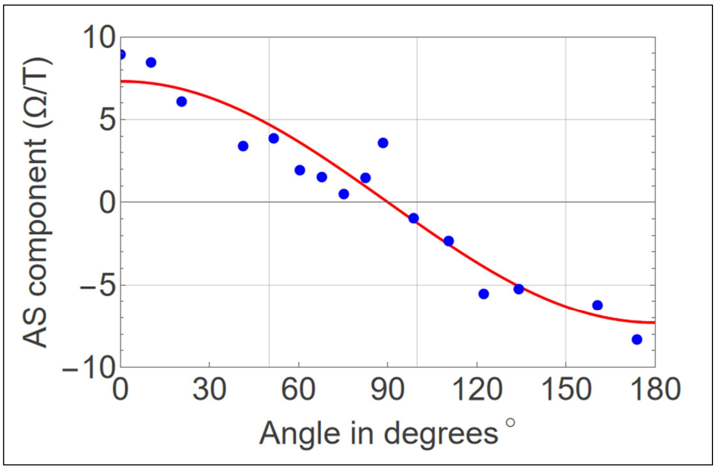

Fig. S2: **Extrinsic origing of the antisymmetric MR.** Angular dependence of the coefficient associated with the antisymmetric (odd-in-B) component of the longitudinal MR for field rotations in the y–z plane. The data follow a clear $\cos\theta$ dependence (solid line), as expected for a small admixture of the Hall voltage into the longitudinal channel due to slight sample or contact misalignment. This behavior identifies the antisymmetric contribution as extrinsic, in contrast to the intrinsic quantum-geometric MR, which is strictly even in B.

Because the intrinsic longitudinal MR in a time-reversal-invariant system is even in B, and, within the quantum-geometric expansion discussed in Sec. 3, does not generate odd-in-B terms, the observed antisymmetric $\cos\theta$ component is most consistently interpreted as a purely extrinsic effect. It originates from a tiny Hall admixture, rather than from an intrinsic magnetotransport mechanism.

4.4 Gate voltage dependence

For a 2D parabolic band, the relevant physical quantities scale with the carrier density n as follows: the Fermi wavevector scales as $k_F = (2\pi n)^{1/2}$, the density of states at the Fermi level is constant, $N(E_F) = m^*/(\pi\hbar^2)$, the FS averaged quantum metric scales as $\langle g_{zz}\rangle_{\mathrm{FS}} \sim 1/k_F \sim 1/\sqrt{n}$ after integration over the 2D FS, and the Drude diffusion coefficient scales linearly with density, $D^D_{zz} \propto n$. Substituting these scaling relations into Eq. (89) yields the gate-dependence of the curvature parameter

$$C(n) \propto \frac{1}{N(E_F)} \cdot \frac{1/\sqrt{n}}{n^2} \propto n^{-5/2}. \tag{154}$$

This result captures the strong decay of the NMR with increasing carrier density, in agreement with experimental observations.

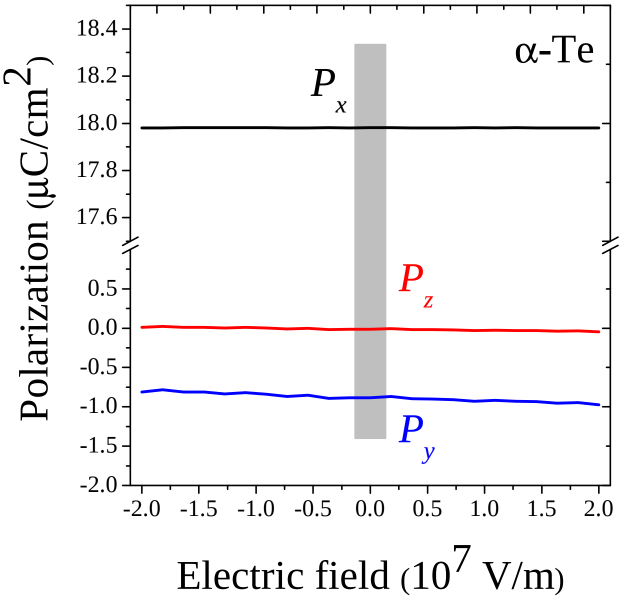

Fig. S3: Polarization components as a function of electric field in the new DFT analysis.

4.5 Gate voltage effects in the polarization

The applied gate voltage does not influence the intrinsic crystallographic polarization field (associated with the lone-pair polar axis) by the out-of-plane displacement field generated by when top- and back-gate voltages are not equal. In our coordinate system, the out-of-plane direction is $\hat{y}$ (perpendicular to the film), while the current flows along $\hat{z}$ and the dominant polarization lies along $\hat{x}$. Therefore, unequal top/back gating primarily produces a component $\mathcal{E}_y$ across the thickness.

In our dual gated device, if we assume the neutral channel approximation the displacement field can be expressed as

$$D = \frac{C_t C_b}{C_t + C_b}(V_{TG} - V_{BG}), \tag{155}$$

where C_t and C_b are the gate capacitances from the top and back gate respectively. We estimate the capacitance value using 20 nm Al_2O_3 as top gate and 90 nm SiO_2 as back gate. ($C_t = 3.98 \times 10^{-3}\,\mathrm{F/m^2}$ and $C_b = 3.84 \times 10^{-4}\,\mathrm{F/m^2}$). The largest displacement field applied to our sample with a back gate voltage 16 V and top gate voltage -4 V, corresponds to

$$D = -7.0 \times 10^{-3}\,\mathrm{C/m^2}. \tag{156}$$

If we assume the relative dielectric constant of Tellurium is 38.3 in the y direction, we obtain

$$E = 2.06 \times 10^{7}\,\mathrm{V/m}. \tag{157}$$

Our DFT calculations confirm that the spontaneous polarization is strongly dominated by the in-plane x component, with a much smaller out-of-plane y component (see Fig. S3). We tested the robustness of the polarization by applying in DFT an external, perpendicular electric field along the $y-$axis and by computing the corresponding dielectric response. The results are shown in Fig. S3: the polarization components are found to be stiff (small susceptibility). Consequently, gate-induced displacement fields perpendicular to the film mainly control electrostatics (carrier distribution) rather than renormalizing the intrinsic crystallographic polarization components.

5 Fitting procedure

The MR data for different magnetic field orientations were fitted using a modified least-squares procedure implemented in Mathematica. For each angle θ, the corresponding dataset was extracted from the experimental measurements and downsampled to approximately 50 points to improve numerical stability. A custom magnetic field range of $B = 1$–8 T was applied to all datasets to ensure consistent comparison.

The fitting model employed was $R(B) = A_0 + F_0|B| - C_0B^2$, where the absolute value term $F_0|B|$ accounts for possible antisymmetric contributions to the MR. The parameters A_0, C_0, and F_0 were obtained via pseudoinverse linear regression applied to the design matrix $X = [1, |B|, -B^2]$.

To ensure the robustness of the fit, quality filters were applied, excluding datasets for which $A_0 \notin [1500, 1850]$ Ω, $C_0 \notin (-1, 5)$ Ω/T^2, or $|F_0| > 20$ Ω/T. The curvature parameter $C_0(\theta)$ was then analyzed as a function of the magnetic field orientation. Angular averages were computed for parallel ($\theta \leq 30°$ or $\geq 150°$) and perpendicular ($60° \leq \theta \leq 120°$) orientations to extract C_z and $C_\perp$, respectively. Finally, these parameters were fitted to a $\cos(2\theta)$ dependence, $C(\theta) = C_{A0} + C_{B0}\cos(2\theta)$, providing a phenomenological description of the angular dependence of the MR curvature.

6 Parish–Littlewood magnetoresistance for $\mathbf{B} \parallel \boldsymbol{\mathcal{E}}$

When $\mathbf{B} \parallel \boldsymbol{\mathcal{E}}$, the intrinsic quantum-geometric channel responsible for the giant negative magnetoresistance (GNMR), which scales as $(\boldsymbol{\mathcal{E}} \times \mathbf{B})^2$, is symmetry-forbidden and therefore completely suppressed. Experimentally, however, we observe in this configuration a small, positive, and symmetric MR in the conduction band.

Since this signal persists even in the absence of intrinsic spin- and geometry-driven mechanisms, it must originate from an extrinsic, orbital source. A natural and well-established candidate is the Parish–Littlewood MR [7], in which spatial inhomogeneities of carrier density or mobility generate a positive, non-saturating MR through effective-medium physics [7, 8]. In tellurene devices, such inhomogeneity is expected on general grounds: the flakes are sufficiently thick that combined top and back gating, together with imperfect screening and finite thickness, produce regions of higher and lower carrier density across the film, as schematically illustrated in Fig. S5. In the presence of a magnetic field, the resulting orbital deflection of carriers leads to current redistribution between these regions, enhancing dissipation.

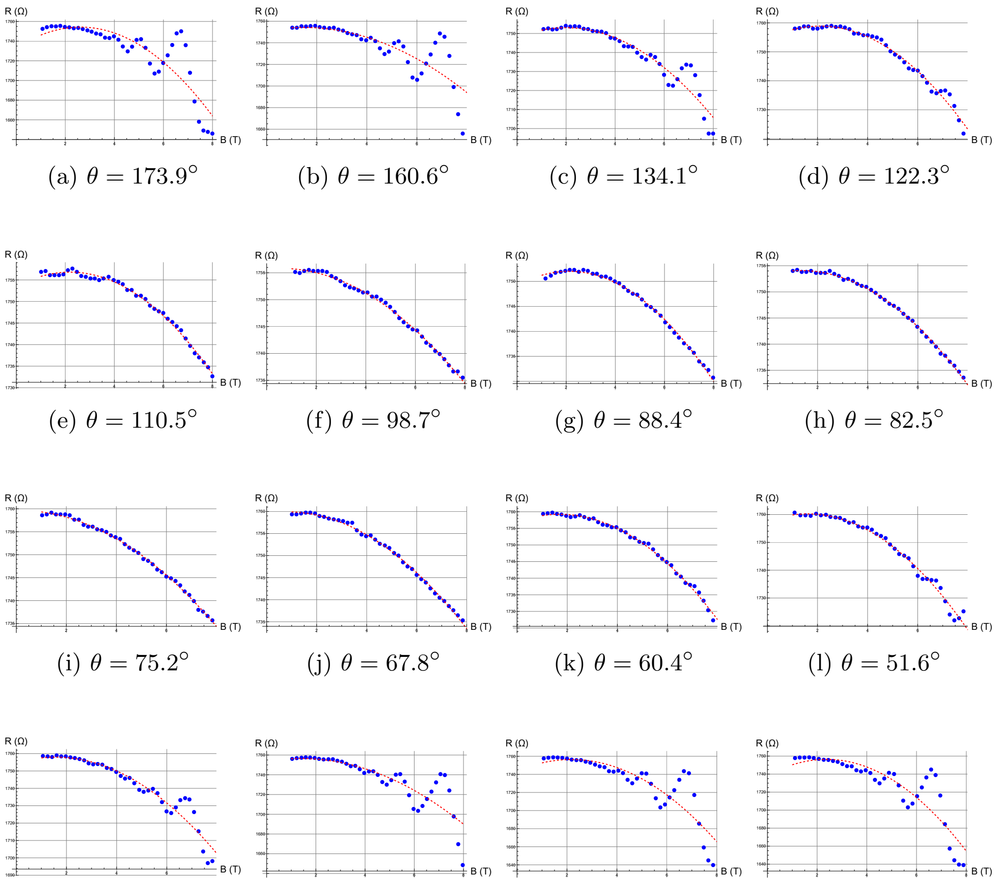

(a) $\theta = 173.9^\circ$ (b) $\theta = 160.6^\circ$ (c) $\theta = 134.1^\circ$ (d) $\theta = 122.3^\circ$

(e) $\theta = 110.5^\circ$ (f) $\theta = 98.7^\circ$ (g) $\theta = 88.4^\circ$ (h) $\theta = 82.5^\circ$

(i) $\theta = 75.2^\circ$ (j) $\theta = 67.8^\circ$ (k) $\theta = 60.4^\circ$ (l) $\theta = 51.6^\circ$

(m) $\theta = 41.3^\circ$ (n) $\theta = 20.6^\circ$ (o) $\theta = 10.3^\circ$ (p) $\theta = 0^\circ$

Fig. S4: Comparison of experimental data and fitted curves for different magnetic field orientations. The blue points represent the measured resistance R as a function of magnetic field B, while the red dashed lines show the corresponding fits. Angles θ indicate the orientation of the magnetic field relative to the sample plane.

6.1 Local Drude transport for $\mathbf{B} = B\hat{\boldsymbol{x}}$

We consider transport along the helical-chain direction (z-axis) in the presence of a uniform in-plane magnetic field $\mathbf{B} = B\hat{\mathbf{x}}$, perpendicular to the current. On mesoscopic length scales, where the system can be regarded as locally homogeneous, each region is described by a standard Drude conductivity tensor in the $y - z$ plane,

$$\hat{\sigma}(\mathbf{r}) = \frac{n(\mathbf{r})e\mu(\mathbf{r})}{1 + [\mu(\mathbf{r})B]^2} \begin{pmatrix} 1 & -\mu(\mathbf{r})B \\ \mu(\mathbf{r})B & 1 \end{pmatrix}_{(y,z)}. \tag{158}$$

Equivalently, the local resistivity tensor reads

$$\hat{\rho}(\mathbf{r}) = \hat{\sigma}^{-1}(\mathbf{r}) = \begin{pmatrix} \rho_{\|}(\mathbf{r}) & \rho_{\perp}(\mathbf{r})\, B \\ -\rho_{\perp}(\mathbf{r})\, B & \rho_{\|}(\mathbf{r}) \end{pmatrix}_{(y,z)}, \tag{159}$$

with

$$\rho_{\|}(\mathbf{r}) = \frac{1}{n(\mathbf{r}) e \mu(\mathbf{r})}, \qquad \rho_{\perp}(\mathbf{r}) = \frac{1}{n(\mathbf{r}) e}. \tag{160}$$

A crucial point is that in a homogeneous Drude metal the longitudinal resistivity $\rho_{zz}(B) = \rho_{\|}$ is strictly field independent for $\mathbf{B} \perp \mathbf{J}$. Thus, any positive MR observed in this configuration must originate from physics beyond a uniform Drude description.

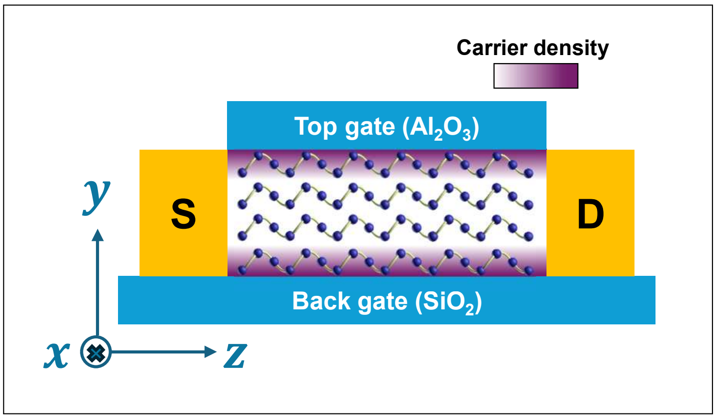

Fig. S5: **Inhomogeneous charge distribution.** Due to the presence of both top and back gates the charge distribution accross the thickness of the tellurene flakes is not homogeneous [9]. For $\mathbf{B} \parallel \hat{\mathbf{x}}$ and $\mathbf{I}$ or $\mathbf{v} \parallel \hat{\mathbf{z}}$ the magnetic component of Lorentz force $q\,\mathbf{v} \times \mathbf{B} \parallel \hat{\mathbf{y}}$ will cause the charge carriers moving horizontally along the helices in a region of a certain density to bounce off regions of different densities, therefore enabling the Parish-Littlewood mechanism.

6.2 Effective-medium description and physical mechanism

We assume that $n(\mathbf{r})$ and $\mu(\mathbf{r})$ vary smoothly on length scales large compared to the mean free path. Each region remains locally Drude-like, but the macroscopic transport must satisfy current continuity in an inhomogeneous medium,

$$\nabla \cdot \mathbf{j}(\mathbf{r}) = 0, \qquad \mathbf{j}(\mathbf{r}) = \hat{\sigma}(\mathbf{r})\, \mathbf{E}(\mathbf{r}). \tag{161}$$

In effective-medium theory, the macroscopic response is captured by an effective tensor $\hat{\rho}_{\text{eff}}$ (or $\hat{\sigma}_{\text{eff}}$), defined such that embedding a typical inhomogeneous region into the effective medium produces no net dipolar field. For a binary mixture of local conductivities $\hat{\sigma}_{1,2}$ occupying area fractions p and $1-p$, this condition leads to the tensor

equation

$$p\left(\hat{\sigma}_1-\hat{\sigma}_{\rm eff}\right)\left(\hat{\sigma}_1+\hat{\sigma}_{\rm eff}\right)^{-1}+(1-p)\left(\hat{\sigma}_2-\hat{\sigma}_{\rm eff}\right)\left(\hat{\sigma}_2+\hat{\sigma}_{\rm eff}\right)^{-1}=0. \tag{162}$$

Physically, in the presence of $\mathbf{B}=B\hat{x}$, carriers experience a Lorentz force along $\pm\hat{y}$. In an inhomogeneous sample, this force pushes carriers from regions of higher density toward regions of lower density, and vice versa, distorting current streamlines. Because different regions possess different local Hall responses $\rho_\perp(\mathbf{r})B$, this mismatch generates internal circulating currents and additional dissipation. Even though each region individually would exhibit no MR, the net result is a positive orbital MR that is entirely classical and spin independent.

6.3 Analytic form of the positive magnetoresistance

We decompose the local resistivities as

$$\rho_{\|}(\mathbf{r})=\rho_{\|}+\delta\rho_{\|}(\mathbf{r}), \qquad \rho_\perp(\mathbf{r})=\rho_\perp+\delta\rho_\perp(\mathbf{r}), \tag{163}$$

where $\rho_{\|}$ and $\rho_\perp$ denote spatial averages. In gated thin films, the dominant inhomogeneity is typically density-driven, so that $\delta\rho_\perp/\rho_\perp \simeq \delta n/n$, while fluctuations of $\rho_{\|}$ may be subleading.

In the Hall-dominated regime,

$$|\rho_\perp B| \gg \rho_{\|}, \tag{164}$$

effective-medium solutions take a robust "quadrature" form,

$$\rho_{\mathrm{eff},zz}(B) \;\simeq\; \sqrt{\rho_{\|}^2+C\left(\delta\rho_\perp\right)_{\rm rms}^2 B^2}, \tag{165}$$

where $(\delta\rho_\perp)^2_{\rm rms}\equiv\langle(\delta\rho_\perp)^2\rangle$ and $C=\mathcal{O}(1)$ is a weakly geometry-dependent constant. At sufficiently large fields this reduces to

$$\rho_{\mathrm{eff},zz}(B) \;\approx\; \sqrt{C}\,(\delta\rho_\perp)_{\rm rms}\,|B| \;=\; \frac{\sqrt{C}}{e}\,\frac{\delta n_{\rm rms}}{n^2}\,|B|, \tag{166}$$

i.e. a symmetric, positive, nonsaturating linear MR.

For the rotations $\mathbf{B}=B(\cos\theta\,\hat{y}+\sin\theta\,\hat{x})$ (from $\hat{y}$ to $\hat{x}$ and back to $-\hat{y}$), the observed symmetric linear-in-$|B|$ component follows a $\sin\theta$ envelope. In this geometry, the effective perpendicular field is $B_\perp=B|\sin\theta|$, and in the Hall-dominated effective-medium regime the longitudinal response takes the form $\Delta R_{zz}^{\rm PL}\propto|B_\perp|$, hence

$$\Delta R_{zz}^{\rm PL}(\theta,B)\propto|B|\,|\sin\theta|. \tag{167}$$

Importantly, this contribution is symmetric in B and manifests itself precisely when the intrinsic quantum-geometric negative MR channel, proportional to $(\boldsymbol{\mathcal{E}}\times\mathbf{B})^2$, is

symmetry-suppressed in the relevant geometry. It is therefore fully consistent with an extrinsic, orbital Parish–Littlewood mechanism, see Fig. S6.

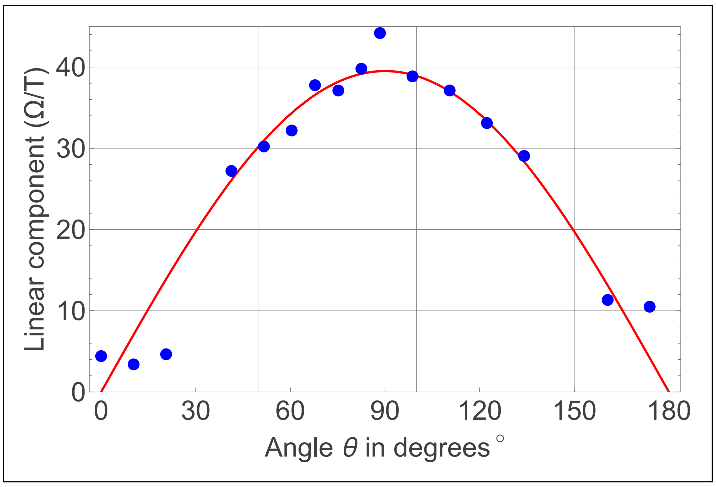

Fig. S6: **Parish–Littlewood MR.** Angular dependence of the coefficient $F(\theta)$ associated with the linear, symmetric component of the longitudinal MR for field rotations in the y–x plane. The solid line shows a $\sin\theta$ dependence, characteristic of an extrinsic Parish–Littlewood MR arising from charge inhomogeneity when the magnetic field acquires a component parallel to the polar axis ($\mathbf{B} \parallel \boldsymbol{\mathcal{E}}$). This angular signature distinguishes the linear positive contribution from the intrinsic quantum-geometric response, which vanishes in this configuration.

7 Other experimental results

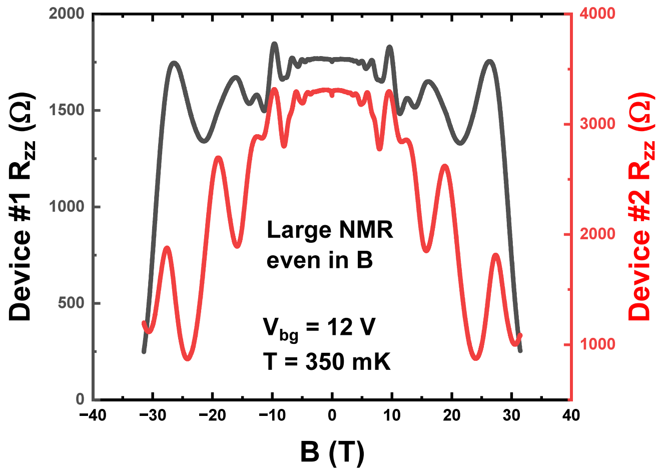

Fig. S7: **GNMR in the conduction band of 2D Te.** GNMR measured in two different devices with similar carrier densities, exhibiting symmetric negative magne-

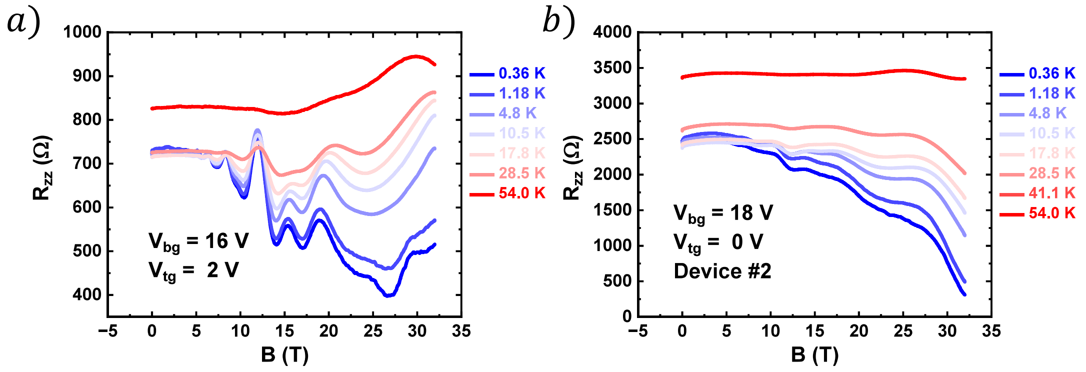

Fig. S8: **Temperature-dependent GNMR in 2D Te.** (a) Temperature dependence of the NMR at higher carrier densities measured in the same device as in Fig. 3d. (b) Temperature dependence of the NMR measured in a different device, showing similar behavior with suppression of NMR at 54 K.

References

[1] Blinowski, J., Rebmann, G., Rigaux, C., Mycielski, J.: Magnetooptical investigation of the conduction band in tellurium. J. Phys. France **38**(9), 1139–1151 (1977) https://doi.org/10.1051/jphys:019770038090113900

[2] Doi, T., Nakao, K., Kamimura, H.: The valence band structure of tellurium. I. The k·p perturbation method. J. Phys. Soc. Jpn. **28**(1), 36–43 (1970) https://doi.org/10.1143/JPSJ.28.36

[3] Doi, T., Nakao, K., Kamimura, H.: The valence band structure of tellurium. II. The infrared absorption. J. Phys. Soc. Jpn. **28**(4), 822–826 (1970) https://doi.org/10.1143/JPSJ.28.822

[4] Nakao, K., Doi, T., Kamimura, H.: The valence band structure of tellurium. III. The Landau levels. J. Phys. Soc. Jpn. **30**(5), 1400–1413 (1971) https://doi.org/10.1143/JPSJ.30.1400

[5] Roth, L.M., Lax, B., Zwerdling, S.: Theory of optical magneto-absorption effects in semiconductors. Physical Review **114**(1), 90–104 (1959) https://doi.org/10.1103/PhysRev.114.90

[6] Yafet, Y.: g factors and spin-lattice relaxation of conduction electrons. Solid State Physics, vol. 14, pp. 1–98. Academic Press (1963). https://doi.org/10.1016/S0081-1947(08)60259-3 . https://www.sciencedirect.com/science/article/pii/S0081194708602593

[7] Parish, M., Littlewood, P.: Non-saturating magnetoresistance in heavily disordered semiconductors. Nature **426**(6963), 162–165 (2003) https://doi.org/10.1038/nature02073

[8] Herring, C.: Effect of random inhomogeneities on electrical and galvanomagnetic measurements. J. Appl. Phys. **31**(11), 1939–1953 (1960) https://doi.org/10.1063/1.1735477

[9] Qiu, G., Niu, C., Wang, Y., Si, M., Zhang, Z., Wu, W., Ye, P.D.: Quantum Hall effect of Weyl fermions in n-type semiconducting tellurene. Nat. Nanotechnol. **15**(7), 585–591 (2020) https://doi.org/10.1038/s41565-020-0715-4
