# Supplementary material for: Gate-tunable giant negative magnetoresistance in tellurene driven by quantum geometry
Source: Nat Commun. 2026 Jun 5;17:5783. doi: 10.1038/s41467-026-74066-0 (PMC13328452; doi:10.1038/s41467-026-74066-0)
Supplement: Supplementary file 2 — Transparent Peer Review file [file 41467_2026_74066_MOESM2_ESM.pdf]

nature portfolio

Peer Review File

Gate-Tunable Giant Negative Magnetoresistance in Tellurene Driven by Quantum Geometry

Corresponding Author: Professor Marcello Silva Neto

This file contains all reviewer reports in order by version, followed by all author rebuttals in order by version.

Version 0:

Reviewer comments:

Reviewer #1

(Remarks to the Author)
The authors reported a gate-tunable giant negative magnetoresistance (NMR) of -90% at 35T in 2D tellurium devices. The observed NMR was attributed to the quantum geometry induced by the cross product of a uniform, lone-pair-induced polarization electric field, E and the applied external field, B, of the form (E x B)^2. Compared to the Berry curvature, the quantum metric tensor has long been overlooked until very recently, for which the transport signatures mainly involve nonlinear transport. Here, through systematic field rotation and variable temperature studies of the high-field magneto transport data on a gate-controlled device, the authors clearly underpinned the empirical vectorial relationship between the electric polarization vector and the external field, establishing a link between NMR and quantum metric. Overall, the experimental data are convincing, and are supported by ample theoretical discussions. I therefore tend to recommend publication of the manuscript in Nature Communciations, provided the authors can address the following comments:
1) Figure 4 makes a comprehensive qualitative illustration to the “spin-drift locking” mechanism proposed by the authors. However, the energy scale provided by an external magnetic field is usually quite weak. It is unclear to me whether an external field of ~20T is sufficient to completely deplete the inner Fermi surface and drastically transform the spin texture. I would suggest the authors to include additional quantitative analysis based on the experimental data (carrier density, mobility, gate voltage, etc), showing that indeed, external magnetic field can produce significant effect on the specific electronic structure of the conduction band Weyl node.
2) Unlike the definition of positive MR that allows infinitely large values, negative MR, by definition, will approach and never reach -1 (or -100%). This is clearly incompatible with the diverging trend of -B^2 dependence. The applicable range of such dependence (deriving from the perturbation theory) deserves further discussion.
3) Previously, appreciable NMR along z-axis in hole-doped Te nanowire/nanofilm devices was also reported by several groups, e.g. PNAS 117 (21) 11337 (2020), Nature Materials 21, 526–532 (2022), which was attributed to the Weyl physics and chiral anomaly. Can the authors fully exclude the contribution of chiral anomaly on the transport of n-doped 2D Te here? If not, the estimation of the anisotropic spin-orbit interaction will be largely affected.
4) The authors should compare the size of the crystallographic polarization electric field along x with the electric field along z applied to the device when top gate and bottom gate voltages are not equal. Will this external electric field add to the inherent polarization field and affect the results?
5) The magnitude and uniformity of this internal polarization electric field have not been directly measured. Due to the symmetric square dependence, left-handed and right-handed Te devices are expected to exhibit similar NMR. Can one design an experiment to directly apply an electric field along x which not only show a direct control of the internal electric field and also demonstrate its dependence on the chirality?

Reviewer #2

(Remarks to the Author)
The manuscript reports gate-tunable giant negative magnetoresistance (MR) in tellurene and proposes a geometric mechanism to explain it. The central theoretical claim is that the internal polarization field of the material, when combined with an external magnetic field, generates an effective “drift–Zeeman” interaction which modifies the band geometry (in particular the quantum metric) and enhances diffusion through an interband geometric contribution to conductivity. The observed MR is then attributed to the field dependence of this geometric term.

The experimental results are interesting. However, the theoretical foundation of the proposed mechanism has several serious issues. In its present form, I do not find the central theoretical claim to be established rigorously enough to warrant publication. My comments are given below.

1) Drift–Zeeman derivation

The derivation of the drift–Zeeman interaction term is not gauge-invariant. Gauge-dependent terms appear in the intermediate steps, and one of them is explicitly discarded. This is not acceptable. The appearance of gauge-dependent contributions indicates an inconsistency in the truncated perturbation theory. Gauge invariance must emerge from a manifestly covariant formulation or from explicit cancellation of all terms at the given order. Simply discarding one term does not restore gauge invariance and leaves the coefficient of the retained term uncontrolled.

Further, the internal polarization field is modeled as a uniform scalar potential -e E.r. Formally, this corresponds to a continuum Stark problem rather than to a periodic inversion-breaking crystal potential. A systematic treatment would require a gauge-covariant Bloch or multiband k·p framework. Since the entire magnetotransport mechanism hinges on this drift–Zeeman term, a fully gauge-consistent derivation is essential. As presented, this is not rigorously established, making its emergence doubtful.

2) Uncontrolled approximations

Several approximations are introduced without identifying a clear small parameter. The quadratic part of the spectrum is discarded while deriving the new term. However, that part also generates magnetic-field-dependent contributions under minimal coupling, which may compete with the proposed mechanism. What justifies neglecting it? Is there a controlled expansion parameter?

Similarly, in deriving the quantum metric contribution, field dependence of the band structure is neglected and zero-field odd-in-momentum symmetry is invoked to simplify expressions. Why is this justified once finite fields are present? Ultimately, when calculating the conductivity, all such terms contribute. Many of them introduce their own field dependence — which is precisely the quantity being measured and analyzed. It must be demonstrated that the neglected field-dependent terms are parametrically smaller than the contribution attributed to the E\cross B term.

3) Regime of validity

The experiment reaches very high magnetic fields and appears to approach the quantum limit. The theory, however, is perturbative in field by construction. Even if one discards this quantum limit, the experimental data span the entire range of semiclassical low to high field values. Why one should expect the low-field theory to remain applicable across this full range is not clear.

4) Geometric contribution

Interband/geometric corrections to transport are standard in multiband systems. While the separation of these contributions is clearly presented and useful, it should be clarified what is genuinely new here, otherwise, it reads as somewhat oversold.

5) Dimensional consistency

The theoretical derivation employs a 3D Weyl-type model, whereas the experimental system appears effectively two-dimensional. If the sample is treated as a quasi-2D thin film (2–20 nm), this must be compared with the magnetic length to assess whether 3D orbital physics is relevant. On the other hand, experimentally, the SdH frequencies do not change upon tilting the magnetic field direction, suggesting strong 2D character (perhaps checking 1/cosθ scaling of the SdH frequency would bolster this). How does one reconcile this?

6) In-plane magnetic field response

The magnetoresistance is not zero when B is parallel to the 2D plane. In the strict 2D limit, orbital Lorentz effects should be suppressed. What remains is spin or spin–orbit physics. In particular, when B is parallel to the internal polarization direction, a small positive MR (~3%) is observed. The origin of this contribution should be discussed, especially since the proposed mechanism appears inactive in this configuration.

Reviewer #3

(Remarks to the Author)
In this manuscript, Marcello B. Silva Neto et al. reported the giant negative magnetoresistance (GNMR) in the n-type Te flakes, which reaches up to 90%, far exceeding the field range (32 T) and magnitude of the 2D electron system reported previously. Especially, they tuned the carrier type (n- and p-) and concentration by using the a highly doped silicon back gate and an Al_2O_3 top gate. Due to the special band structure of Te, the conduction band splits and crosses at the H point to form a Weyl node, thus the charge carriers having non-trivial quantum geometry, while the valence band shows no such crossing. It was observed the n-type regime exhibits negative magnetoresistance (NMR), while p-type regime shows positive

magnetoresistance (PMR). These experimental results are reliable and helpful for understanding the mechanisms of NMR or PMR emerging in 2D electron systems. Another, the authors proposed that the observed gate-tunable, parabolic GNMR in Te exhibiting Rzz/R0 = -βg(ε× B)2 dependence arises from a quantum geometric enhancement of carrier diffusion, combined with a unique magneto-electric spin interaction. The theoretical explanation is new and reasonable. I agree this manuscript to be published in Nature Commun after addressing my following concern.

1.Why the Rzz/R0 is only dependent with the macroscopic polarization electric field,ε? Which may include the applied electric field along the current?

Version 1:

Reviewer comments:

Reviewer #1

(Remarks to the Author)
The authors have addressed all my comments in the response letter. Before fully supporting the publication of the manuscript in Nature Communications, I would like the authors to include their response to my comment #4 (regarding the role of applied gate voltage Ey and the built-in electric polarization Ex) into supplementary.

Besides, I also notice a minor caveat in the introduction part,
"Most remarkably, in Weyl semimetals (e.g., WTe2 films), NMR reaches –60% at 60 T under pulsed fields, driven by the chiral anomaly, in the ultraquantum limit [13]."
I thought Ref. 13 mainly reported colossal positive MR in ultraclean WTe2 single crystal. Can the authors check again this statement and citation?

Reviewer #2

(Remarks to the Author)
The revisions have improved several aspects of the manuscript. Many of the points have been addressed; however, two key issues (points 1 and 5) still remain unresolved.

1. Derivation of the drift–Zeeman term.

While the revised derivation removes the explicit gauge inconsistency, it still relies on treating the internal polarization as a uniform scalar potential -e E·r. In my opinion, this is not the correct starting point—the identification of E with the internal polarization is heuristic. In a crystal, inversion breaking enters through the periodic potential and generates spin–orbit coupling via (\nabla V x p).\sigma. A systematic multiband Bloch or k.p treatment, followed by projection, would in general produce all symmetry-allowed B-dependent terms at the same order. The present approach isolates a specific term without deriving the full effective Hamiltonian or establishing that other competing terms are parametrically smaller. As a result, the microscopic origin and relative importance of the proposed term are not established in a controlled way.

The foolproof procedure is not difficult to implement. It involves the same SW transformation as the authors have now used, but with a different starting point. I am now confident that the interesting term—the drift–Zeeman term—is real and would emerge even within such a derivation, but other new terms may also appear (similar to the new drift–orbit term that the authors have found upon revision).

5. Dimensionality and effective Hamiltonian.

The authors have commented, "the system exhibits quasi-2D transport while inheriting its quantum-metric properties from an underlying 3D Weyl band structure." This does not make sense to me. The geometry is clearly defined (chains along z, polarization along x, confinement along y). However, the model retains a fully three-dimensional dispersion, treating kx and ky on equal footing and keeping ky continuous. This is inconsistent with the experimental evidence indicating a two-dimensional Fermi surface. In this regime, motion along the confinement direction should be quantized, and the appropriate low-energy description should be a reduced Hamiltonian H_eff(kx, kz). It is therefore unclear whether the results obtained from the 3D model remain valid after proper dimensional reduction. Once again, using the 3D model to derive the new drift–Zeeman term is acceptable, but applying the same theory to a clearly 2D system is not consistent. If the authors believe that nothing changes with dimensionality, then why not rederive the same physics with 2D bands in mind? This would help clarify the issue.

Overall, I am sympathetic to the central idea, which is interesting, and the experimental results are not without merit. However, the theoretical framework needs to be placed on a more systematic footing before the proposed mechanism can be considered established.

Reviewer #3

(Remarks to the Author)

I have carefully read the revised manuscript by M. B. Silva Neto et al, and found that the authors had modified the manuscript according to all the comments of three reviewers one by one. I agree to recommend the revised manuscript to be published in Nature Communication.

Version 2:

Reviewer comments:

Reviewer #1

(Remarks to the Author)
The authors have addressed all my comments. I can therefore fully support the publication of the manuscript in Nature Communications.

Reviewer #2

(Remarks to the Author)
The authors have addressed the two points I raised in my last report. The theory is now much more controlled. I think the manuscript can now be published.

Open Access This Peer Review File is licensed under a Creative Commons Attribution 4.0 International License, which permits use, sharing, adaptation, distribution and reproduction in any medium or format, as long as you give appropriate credit to the original author(s) and the source, provide a link to the Creative Commons license, and indicate if changes were made.
In cases where reviewers are anonymous, credit should be given to 'Anonymous Referee' and the source.
The images or other third party material in this Peer Review File are included in the article's Creative Commons license, unless indicated otherwise in a credit line to the material. If material is not included in the article's Creative Commons license and your intended use is not permitted by statutory regulation or exceeds the permitted use, you will need to obtain permission directly from the copyright holder.
To view a copy of this license, visit https://creativecommons.org/licenses/by/4.0/

Response to Reviewer #1

We thank the Reviewer #1 for the careful evaluation of our and for the valuable suggestions. In the following, we provide detailed, point-by-point responses to all the issues raised. The corresponding changes have been made in the revised manuscript and supplemental information.

1. *"Figure 4 makes a comprehensive qualitative illustration to the "spin-drift locking" mechanism proposed by the authors. However, the energy scale provided by an external magnetic field is usually quite weak. It is unclear to me whether an external field of 20T is sufficient to completely deplete the inner Fermi surface and drastically transform the spin texture. I would suggest the authors to include additional quantitative analysis based on the experimental data (carrier density, mobility, gate voltage, etc), showing that indeed, external magnetic field can produce significant effect on the specific electronic structure of the conduction band Weyl node."*

   Answer: We thank the Reviewer #1 for this important observation. We would like to emphasize that the giant negative magnetoresistance (GNMR) reported in our work does not rely on a complete depletion of the inner Fermi surface nor on a drastic reconstruction of the spin texture by the magnetic field. Fig. 4 is merely illustrative of what would happen upon an extremely large coercive magnetic field. This is now clearly stated in the manuscript after Eq. (7) in Section 3.3.

   Following the suggestion of Reviewer #1, we provide two quantitative estimates for this effect, derived from two different perspectives.

   First, since it is technically challenging to include magnetic fields in DFT calculations, we adopted an alternative approach. We ran a new DFT simulation forcing the system to feature a nonzero magnetization along the helical axis, $M_z \neq 0$, and letting it relax until it reaches its new ground state. The resulting band structure including both conduction and the two uppermost valence bands is shown in Fig. 1. As the Reviewer #1 can observe, the primary effect of inducing $M_z \neq 0$ in the conduction band of tellurene is a shift of the Weyl node along k_z. This numerical result is in precise agreement with the shift predicted by the proposed Drift-Zeeman coupling, $H_{DZ} = \gamma(\boldsymbol{\mathcal{E}} \times \mathbf{B}) \cdot \sigma$. Given that the typical energy scales provided by an external

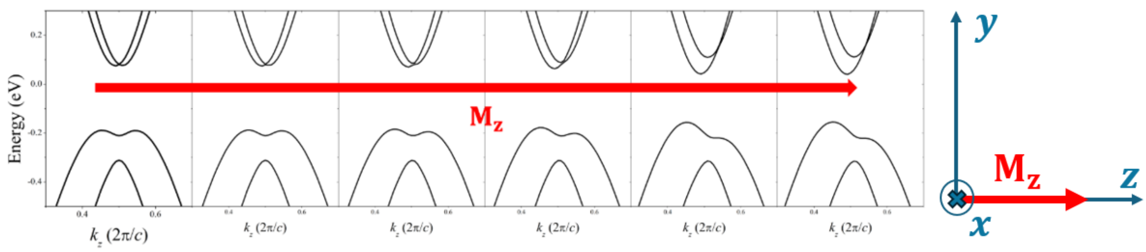

Figure 1. Evolution of the band structures obtained from the new DFT simulation, relaxing the structure while increasing the magnetization, M_z.

magnetic field are usually quite weak, the Weyl node remains always very close to the high-symmetry H point, corresponding to the first two panels in Fig. 1. Consequently, the GNMR is driven almost entirely by the strongly diverging character of the quantum metric near the Weyl node.

Second, by using experimentally determined parameters, we provide an independent quantitative estimate for the validity of the low-field regime. For our typical devices, the carrier density obtained upon gating is $n \simeq 10^{12}\,\mathrm{cm}^{-2}$, corresponding to a Fermi wave vector $k_F \sim 10^6\,\mathrm{cm}^{-1}$, while the mobility is $\mu \sim 2000 - 4000\,\mathrm{cm}^2/\mathrm{Vs}$ and the flake thickness is 2–20 nm. From DFT we estimate the lone-pair polarization is of order $\mathcal{P} \sim 20\mu\mathrm{C/cm}^2$, and from the microscopic model we obtain $\gamma \sim 2e^2\lambda_z^3/\hbar\Delta^3$, with $\lambda_z \sim 10^{-9}\,\mathrm{eV\,cm}$ and a conservative bandwidth $\Delta \simeq 10 - 50\,\mathrm{meV}$. These values yield a characteristic field scale $B^* \sim \lambda_z k_F/\gamma_z \mathcal{E}_x$ — defined as the field at which the shift in k_z produced by H_{DZ} becomes of the order of k_F and the field-dependent quantum metric begins to saturate — given by $B^* \sim 40$–$50\,\mathrm{T}$. Thus, since the experimentally explored regime of magnetic fields ranges up to $\sim 0 - 20\,\mathrm{T}$, the system remains safely in the weak field regime $B \ll B^*$ (first two/three panels of the figure above) when the Weyl node is closer to the H-point than to the Fermi wave vector k_F.

2. *"Unlike the definition of positive MR that allows infinitely large values, negative MR, by definition, will approach and never reach -1 (or -100%). This is clearly incompatible with the diverging trend of $-B^2$ dependence. The applicable range of such dependence (deriving from the perturbation theory) deserves further discussion."*

   Answer: We fully agree with Reviewer #1 that a negative MR of the form $\Delta\rho \propto -B^2$, if extrapolated to arbitrarily large magnetic fields, would be unphysical, since by definition the resistivity cannot decrease without bound. We emphasize, however, that the $-B^2$ dependence discussed in the manuscript is explicitly a low-field expansion, derived within perturbation theory and therefore valid only in the regime $B \ll B^* \approx$ 40–50 T, the characteristic saturation field, derived above, of the quantum-geometric mechanism.

   At higher magnetic fields, beyond the perturbative regime, the theory predicts a rapid saturation of the MR rather than an unbounded decrease. This saturation arises from the field dependence of the quantum metric and from the necessity of calculating the (coupled) band-structure and magnetization self-consistently. More importantly, for $B \gtrsim 20$ T and even before the onset of saturation at $\approx$ 40–50 T, the system enters the quantum limit, where a semiclassical description based on Fermi-surface dynamics breaks down and a proper Landau-level treatment is required. Since our experimental data showing negative MR lie below this scale, the present work focuses on the controlled low-field regime where the quadratic negative MR naturally emerges.

3. *"Previously, appreciable NMR along z-axis in hole-doped Te nanowire/nanofilm devices was also reported by several groups, e.g. PNAS 117 (21) 11337 (2020), Nature Materials 21, 526–532 (2022), which was attributed to the Weyl physics and chiral anomaly. Can the authors fully exclude the contribution of chiral anomaly on the transport of n-doped 2D Te here? If not, the estimation of the anisotropic spin-orbit*

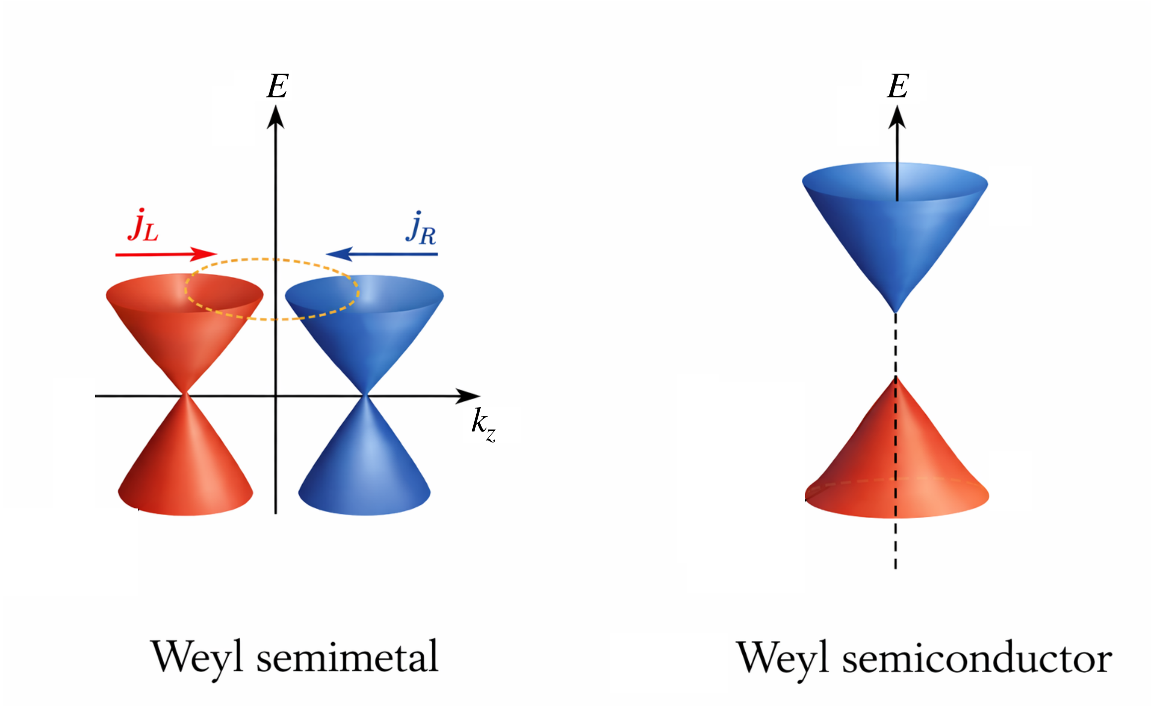

Figure 2. Generic band structures for a Weyl semimetal and a Weyl semiconductor.

interaction will be largely affected."

Answer: We can safely exclude the chiral anomaly as a relevant mechanism for the negative MR reported here in the conduction band of tellurene on both conceptual and experimental grounds. Chiral anomaly is a transport phenomenon specific to Weyl semimetals, see Fig. 2, where pairs of Weyl nodes of opposite topological charge are separated in momentum space (typically due to broken time-reversal symmetry). In that case, a finite $\mathbf{E} \cdot \mathbf{B}$ generates an imbalance of chemical potential between nodes of opposite chirality, leading to charge pumping between them and a negative MR for $\mathbf{B} \parallel \mathbf{I}$.

Tellurene, by contrast, is a Weyl semiconductor: time-reversal symmetry is preserved, and the Weyl nodes are separated in energy, not in momentum. In the conduction band relevant to the present experiment there is a single Weyl node, carrying a single topological charge, with no partner node of opposite chirality at the same energy. As a result, there is no meaningful notion of chiral charge imbalance or internode pumping, and the chiral anomaly mechanism (as described in the previous paragraph) is fundamentally absent.

Experimentally, the absence of other chiral effects is reinforced by the observed symmetry of another previously studied effect, the chiral unidirectional magnetoresistance or electrical magnetochiral anisotropy ($\chi-$eMChA). Chiral magnetotransport $\mathbf{B} \cdot \mathbf{I}$ has been discussed and thoroughly demonstrated for hole-doped bulk Te and nanowires, through charge to spin conversion or current induced magnetization. This is usually associated to a very large and almost collinear magnetic texture $\mathbf{m}(\mathbf{k}) \parallel k_z$ in the valence band H_4 which shifts the Fermi surface uppon an externally applied magnetic field. In n-doped 2D Te devices, however, the magnetic texture is radial and no eMChA

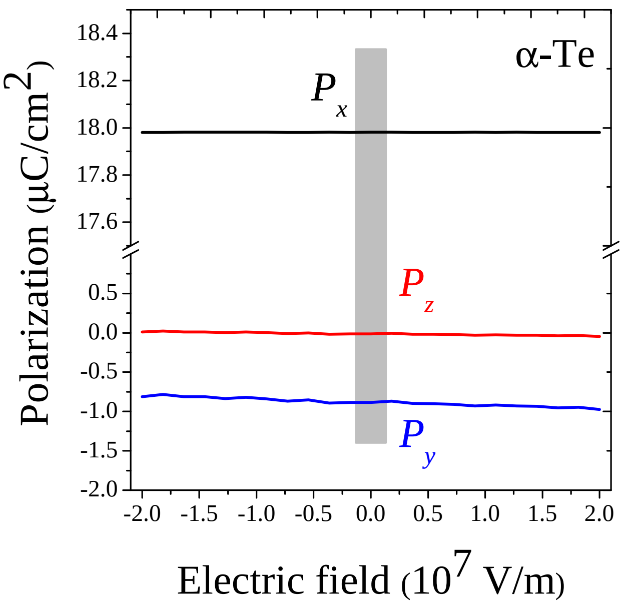

Figure 3. Polarization components as a function of electric field in the new DFT analysis.

of the chiral type has been observed for $\mathbf{B} \parallel \mathbf{I}$, where a chiral contribution would be maximal. Instead, the eMChA is identically zero for $\mathbf{B} \parallel \mathbf{I}$, appears only for $\mathbf{B} \perp \mathbf{I}$ and $\mathbf{B} \perp \mathbf{P}$, and has been explained recently by a polar, quantum-geometric mechanism, $(\mathbf{P} \times \mathbf{B}) \cdot \mathbf{I}$, a nonlinear extension to the one proposed here for the quantum-geometric, linear GNMR (see our Phys. Rev. Lett. 135, 106602 (2025)).

4. *"The authors should compare the size of the crystallographic polarization electric field along x with the electric field along z applied to the device when top gate and bottom gate voltages are not equal. Will this external electric field add to the inherent polarization field and affect the results?"*

   Answer: We thank the Reviewer #1 for this insightful point. The relevant comparison is between the intrinsic crystallographic polarization field (associated with the lone-pair polar axis) and the out-of-plane displacement field generated when top- and back-gate voltages are not equal. In our coordinate system, the out-of-plane direction is $\hat{y}$ (perpendicular to the film), while the current flows along $\hat{z}$ and the dominant polarization lies along $\hat{x}$. Therefore, unequal top/back gating primarily produces a component $\mathcal{E}_y$ across the thickness.

   In our dual gated device, if we assume the neutral channel approximation the displacement field can be expressed as

$$D = \frac{C_t C_b}{C_t + C_b}(V_{TG} - V_{BG}), \quad (1)$$

   where C_t and C_b are the gate capacitances from the top and back gate respectively. We estimate the capacitance value using 20 nm Al_2O_3 as top gate and 90 nm SiO_2 as back gate. ($C_t = 3.98 \times 10^{-3}\,\mathrm{F/m^2}$ and $C_b = 3.84 \times 10^{-4}\,\mathrm{F/m^2}$). The largest displacement field applied to our sample with a back gate voltage 16 V and top gate voltage -4 V,

corresponds to

$$D = -7.0 \times 10^{-3}\,\mathrm{C/m^2}. \tag{2}$$

If we assume the relative dielectric constant of Tellurium is 38.3 in the y direction, we obtain

$$E = 2.06 \times 10^{7}\,\mathrm{V/m}. \tag{3}$$

We ran new DFT calculations and they confirm that the spontaneous polarization is strongly dominated by the in-plane x component, with a much smaller out-of-plane y component (see Fig. 3). We tested the robustness of the polarization by applying in DFT an external, perpendicular electric field along the $y-$axis and by computing the corresponding dielectric response. The results are shown in Fig. 3: the polarization components are found to be stiff (small susceptibility). Consequently, gate-induced displacement fields perpendicular to the film mainly control electrostatics (carrier distribution) rather than renormalizing the intrinsic crystallographic polarization components.

5. *"The magnitude and uniformity of this internal polarization electric field have not been directly measured. Due to the symmetric square dependence, left-handed and right-handed Te devices are expected to exhibit similar NMR. Can one design an experiment to directly apply an electric field along x which not only show a direct control of the internal electric field and also demonstrate its dependence on the chirality?"*

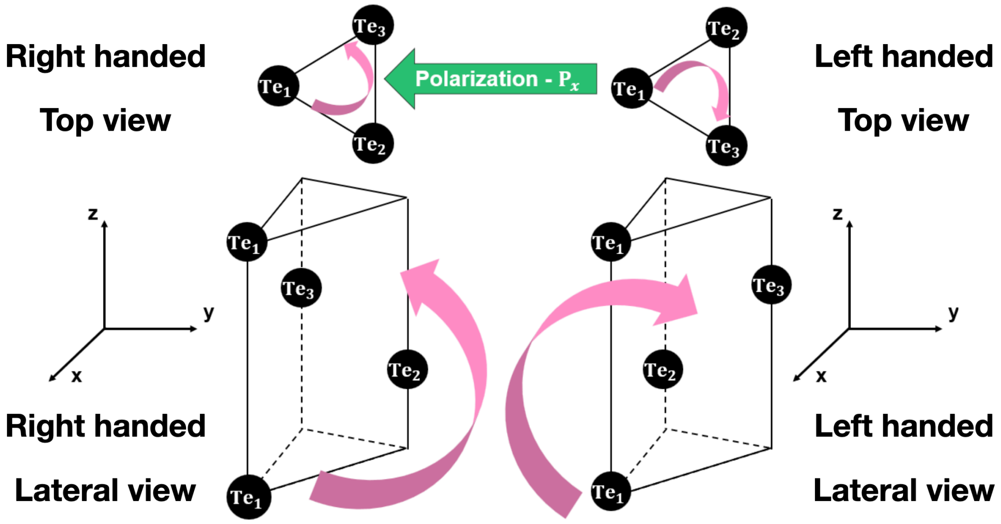

Figure 4. Tellurium helices in the $\alpha-$ structure (with the triangle apex along the $+\hat{\mathbf{x}}$ direction) are featured with the same polarization component P_x even for the case of two opposite chiralities.

Answer: We agree that, because our dominant NMR channel scales as $(\boldsymbol{\mathcal{E}} \times \mathbf{B})^2$, the response is even under $\boldsymbol{\mathcal{E}} \to -\boldsymbol{\mathcal{E}}$ and therefore one expects that $\alpha-$type and $\alpha'-$type samples (related by an inversion operation) to exhibit identical NMR in the geometry governed by the dominant in-plane polarization component. Furthermore, the leading polarization component is along $\hat{x}$ and is chirality-blind: for a given structural variant

(e.g. the two stacking/orientation variants often denoted α or α', in which the triangular motifs are oriented toward $+x$ or $-x$, the magnitude of the x component is the same for left- and right-handed helices, see Fig. 4). This is precisely why the main effect reported here does not, by itself, provide a direct chirality discriminator.

Concerning chirality sensitivity, we fully agree with the Reviewer #1 that one can in principle devise experiments to externally control perpendicular electric field component $\mathcal{E}_y$, since chirality-dependent signatures are expected to couple most directly to the subleading polarization components permitted by symmetry (in particular such out-of-plane component), whose magnitude may depend on the handedness and stacking of the helical motifs. A dedicated study designed to resolve these smaller components would be required to unambiguously demonstrate chirality selectivity. While this is beyond the scope of the present manuscript (which focuses on the dominant x-polar axis and its consequences for magnetotransport), the proposed direction is promising and motivates future experiments.

Response to Reviewer #2

We thank the Reviewer #2 for their careful evaluation of our manuscript and for the insightful criticisms, that help us to significantly improve the clarity and rigor of our work.

In the following, we provide detailed, point-by-point responses to all the issues raised. We have carefully incorporated the corresponding changes into the revised manuscript and supplemental information.

1. Drift-Zeeman derivation – *"The derivation of the drift–Zeeman interaction term is not gauge-invariant. Gauge-dependent terms appear in the intermediate steps, and one of them is explicitly discarded. This is not acceptable. The appearance of gauge-dependent contributions indicates an inconsistency in the truncated perturbation theory. Gauge invariance must emerge from a manifestly covariant formulation or from explicit cancellation of all terms at the given order. Simply discarding one term does not restore gauge invariance and leaves the coefficient of the retained term uncontrolled.*

   Further, the internal polarization field is modeled as a uniform scalar potential $-e\mathbf{E}\cdot\mathbf{r}$. Formally, this corresponds to a continuum Stark problem rather than to a periodic inversion-breaking crystal potential. A systematic treatment would require a gauge-covariant Bloch or multiband $\mathbf{k}\cdot\mathbf{p}$ framework. Since the entire magnetotransport mechanism hinges on this drift–Zeeman term, a fully gauge-consistent derivation is essential. As presented, this is not rigorously established, making its emergence doubtful."

   Answer: We agree with Reviewer #2 that gauge invariance cannot be broken perturbatively. In the revised manuscript we have adopted a manifestly gauge invariant approach through the use of a Schrieffer–Wolff transformation. As a result, the spurious gauge non-invariant terms present in the previous Rayleigh-Schrödinger perturbation scheme are exactly canceled, as demonstrated in the Supplementary Information. Consequently, the $\lambda\mathbf{k}\cdot\boldsymbol{\sigma}$ part of the Hamiltonian yields only the gauge-invariant, Drift-Zeeman coupling

   $$H_{DZ} = \gamma(\boldsymbol{\mathcal{E}}\times\mathbf{B})\cdot\boldsymbol{\sigma}. \tag{4}$$

   Regarding the second point by Reviewer #2, we emphasize that the revised derivation is fully consistent with a gauge-covariant multiband Bloch framework. The Schrieffer–Wolff (SW) transformation is precisely the standard tool used to derive effective low-energy Hamiltonians in multiband $\mathbf{k}\cdot\mathbf{p}$ theory while preserving gauge invariance order by order. In this approach, the coupling to external electromagnetic fields is introduced through the kinetic momentum and projected onto the low-energy Bloch subspace in a manifestly covariant manner, ensuring that all gauge-dependent contributions generated at intermediate steps cancel exactly. As a result, the effective drift–Zeeman term emerges as a well-defined, gauge-invariant operator acting within the Bloch-band manifold, independent of the choice of gauge or representation. The use of the scalar potential $-e\boldsymbol{\mathcal{E}}\cdot\mathbf{r}$ should therefore be understood as the long-wavelength limit of the periodic inversion-breaking crystal potential already encoded in the multiband $\mathbf{k}\cdot\mathbf{p}$

description, rather than as a continuum Stark problem.

2. Uncontrolled approximations – *"Several approximations are introduced without identifying a clear small parameter. The quadratic part of the spectrum is discarded while deriving the new term. However, that part also generates magnetic-field-dependent contributions under minimal coupling, which may compete with the proposed mechanism. What justifies neglecting it? Is there a controlled expansion parameter?*

   Similarly, in deriving the quantum metric contribution, field dependence of the band structure is neglected and zero-field odd-in-momentum symmetry is invoked to simplify expressions. Why is this justified once finite fields are present? Ultimately, when calculating the conductivity, all such terms contribute. Many of them introduce their own field dependence — which is precisely the quantity being measured and analyzed. It must be demonstrated that the neglected field-dependent terms are parametrically smaller than the contribution attributed to the $E \times B$ term."

   Answer: In the revised manuscript we have addressed these important points explicitly.

   First, following the Reviewer's suggestion, we have included the previously neglected quadratic contribution to the Hamiltonian and repeated the gauge-invariant perturbation theory based on the Schrieffer–Wolff transformation. As anticipated by the Reviewer, this produces an additional drift-related term in the effective Hamiltonian, which we denote as the *Drift–Orbit interaction*

$$H_{DO} = \kappa(\boldsymbol{\mathcal{E}} \times \mathbf{B}) \cdot \mathbf{k}, \tag{5}$$

   which naturally accompanies the spin-dependent drift–Zeeman term

$$H_{DZ} = \gamma(\boldsymbol{\mathcal{E}} \times \mathbf{B}) \cdot \sigma. \tag{6}$$

   The term H_{DO} produces a uniform shift of the group velocity

$$v(\mathbf{k}) \to v(\mathbf{k}) + \frac{\kappa}{\hbar}(\boldsymbol{\mathcal{E}} \times \mathbf{B}), \tag{7}$$

   consistent with the classical drift velocity of a charged particle in crossed electric and magnetic fields and with the shifted band structure. The full derivation is now presented in the revised Supplementary Information.

   Second, regarding the field dependence of the quantum metric, the semiclassical expansion is controlled by the small momentum scale

$$\delta = \gamma_z \mathcal{E}_x B / \lambda_z. \tag{8}$$

   Expanding the field-dependent quantum metric yields

$$g_{zz}(\mathbf{k}, B) = g_{zz}^{(0)} + \delta\, \partial_{k_z} g_{zz}^{(0)} + \frac{\delta^2}{2} \partial_{k_z}^2 g_{zz}^{(0)} + \mathcal{O}(\delta^3). \tag{9}$$

   Since $g_{zz}^{(0)}$ is even in k_z, the first derivative is odd and therefore its Fermi-surface average vanishes. The leading correction to the Fermi-surface average is therefore quadratic in the field,

$$\langle g_{zz}(\mathbf{k}, B)\rangle_{FS} B^2 = \langle g_{zz}^{(0)}\rangle_{FS} B^2 + \mathcal{O}(B^4). \tag{10}$$

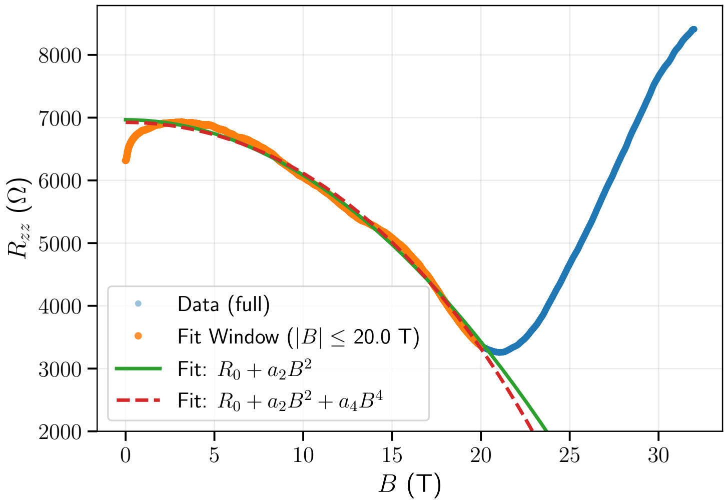

Figure 5. Field dependence in the quantum metric. Fit of the giant negative magnetoresistance for $V_{bg} = 16$V and $V_{tg} = -4$V as a function of the magnetic field $\mathbf{B}$ using both quadratic, B^2, and quartic, B^4, powers in the expansion of $g_{zz}(\mathbf{B})$. As we can see, below the crossover field $B^* \sim \lambda_z k_F/\gamma_z \mathcal{E}_x$ the behavior corresponds to an exact parabolic drop of resistance, as obtained from the simplified $g_{zz}(0)$ limit.

Thus the coefficient of the quadratic magnetoresistance is controlled entirely by the zero-field quantum metric, while field dependence of the metric produces only higher-order corrections. A detailed derivation of this expansion and of the associated symmetry analysis is provided in the Supplementary Information, and we show the result of the B^2 and B^4 quantum metric fits of the experimental data in Fig. 5, for clarity.

Third, we clarify the role of the field-dependent Fermi surface. In the present geometry ($\boldsymbol{\mathcal{E}} \parallel \hat{x}$ and $\mathbf{B} \parallel \hat{y}$), the field enters the band dispersion only through the time-reversal-even combination $(\boldsymbol{\mathcal{E}} \times \mathbf{B})^2$. Consequently,

$$\delta\varepsilon(\mathbf{k}; B) = c_2(\mathbf{k})B^2 + c_4(\mathbf{k})B^4 + \cdots , \tag{11}$$

so that the shift of the Fermi surface generates only even-in-B corrections to transport coefficients. Therefore it cannot produce a linear-in-B longitudinal magnetoresistance. The detailed expansion of the Fermi-surface average confirming this statement is given in the Supplementary Information.

Finally, regarding the small antisymmetric component observed experimentally for rotations in the yz plane, we show that it is naturally explained by a small Hall admixture due to contact misalignment. Writing

$$R_{zz}^{\text{meas}}(\theta, B) \simeq R_{zz}(\theta, B) + \delta\, R_{zy}(\theta, B), \tag{12}$$

with $\delta \ll 1$, one obtains

$$R_{zz}^{\text{AS}}(\theta, B) \approx \delta R_H B \cos\theta, \tag{13}$$

which reproduces the observed $\cos\theta$ dependence, which we display in Fig. 6 for completeness. Since intrinsic longitudinal magnetoresistance in a time-reversal-invariant

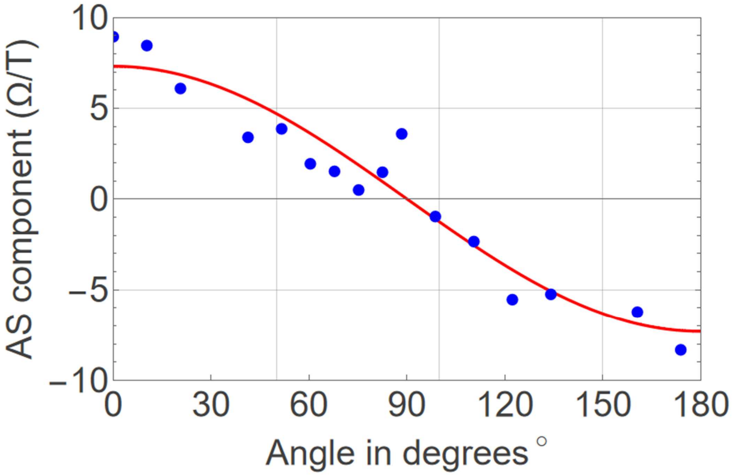

Figure 6. Angular dependence of the coefficient associated with the antisymmetric (odd-in-B) component of the longitudinal magnetoresistance for field rotations in the y–z plane.

system is even in B, this antisymmetric component is most consistently interpreted as an extrinsic Hall admixture rather than an intrinsic transport mechanism.

Additional derivations and symmetry analyses supporting these statements are now included in the revised Supplementary Information.

3. Regime of validity – *"The experiment reaches very high magnetic fields and appears to approach the quantum limit. The theory, however, is perturbative in field by construction. Even if one discards this quantum limit, the experimental data span the entire range of semiclassical low to high field values. Why one should expect the low-field theory to remain applicable across this full range is not clear."*

   Answer: As mentioned in the previous item, the field dependence of the quantum metric enters through the shift $\lambda_z k_z \to \lambda_z k_z + \gamma_z \mathcal{E}_x B$ in the denominator of $g_{zz}(\mathbf{k}, B)$. A natural crossover (saturation) scale B^* is therefore set by the condition that the shift becomes comparable to the typical Fermi momentum k_F

$$\gamma_z \mathcal{E}_x B^* \sim \lambda_z k_F \qquad \Rightarrow \qquad B^* \sim \frac{\lambda_z k_F}{\gamma_z \mathcal{E}_x}. \tag{14}$$

   The microscopic estimate for the drift–Zeeman coefficient, $\gamma_z \simeq 2e^2\lambda_z^3/(\hbar\Delta^3)$, gives us

$$B^* \sim \frac{\hbar\Delta^3}{2e^2\lambda_z^2}\,\frac{k_F}{\mathcal{E}_x}. \tag{15}$$

   With representative parameters for tellurene, $\lambda_z \simeq 10^{-9}\,\mathrm{eV\,cm}$, $k_F \simeq 10^6\,\mathrm{cm}^{-1}$, and taking a conservative bandwidth scale $\Delta = 50$ meV, one obtains

$$B^* \approx \frac{4.1 \times 10^{10}}{\mathcal{E}_x\,[\mathrm{V/m}]}\ \mathrm{T} \sim 40 - 50\ \mathrm{T}, \tag{16}$$

for an internal effective electric field of order $\mathcal{E}_x \sim 10^9$ V/m (a GV/m scale is natural for microscopic polar/lone-pair fields even after screening, and agrees with our own DFT calculations for the polarization $\mathbf{P} \sim 20\mu\mathrm{C/cm}^2$), i.e. at least twice larger than the field scale (~ 20 T) where we concentrated our fitting procedures and where the experimental curves exhibit a minimum associated to the onset of the quantum regime and Landau level physics (which is beyond the scope of this research work).

4. Geometric contribution – *"Interband/geometric corrections to transport are standard in multiband systems. While the separation of these contributions is clearly presented and useful, it should be clarified what is genuinely new here, otherwise, it reads as somewhat oversold."*

   Answer: While interband effects are common, a sizable quantum metric is *not* generic. In ordinary semiconductors such as Si, Ge, or GaAs, with interband gaps of order 0.5–1 eV or larger, the quantum metric either vanishes identically or is parametrically suppressed by large energy denominators. A simple estimate illustrates this point: quantum-metric contributions scale roughly as $(v/\Delta)^2$, where v is an interband velocity matrix element and Δ is the relevant band gap. For GaAs or Si, taking $\Delta \sim 1$ eV and typical velocities yields corrections that are orders of magnitude smaller than the Drude conductivity and therefore experimentally negligible in longitudinal transport.

   In contrast, tellurene hosts low-energy Weyl fermions in the conduction band enforced by crystal symmetry. Near a Weyl node the Bloch wavefunctions vary rapidly in momentum space, and the quantum metric becomes large and sharply peaked. This enhancement is not accidental: it is tied to the topological band structure and to chiral nature of the material. Consequently, the quantum-metric contribution survives in the semiclassical regime and can compete with (or even dominate over) conventional transport channels.

   We also emphasize that the mechanism discussed here is qualitatively distinct from previously studied geometric transport effects. It does not rely on Berry curvature dipoles, anomalous velocities, or transverse responses, but instead on a quantum-metric correction to diffusion enabled by an intrinsic drift–Zeeman–type coupling in a polar Weyl system. To the best of our knowledge, this is the first experimental realization of a regime in which the quantum metric—rather than the Berry curvature—controls the dominant magnetoresistance signal. Most importantly, in linear response; while the majority of quantum metric related publications have been associated to nonlinear effects.

5. Dimensional consistency – *"The theoretical derivation employs a 3D Weyl-type model, whereas the experimental system appears effectively two- dimensional. If the sample is treated as a quasi-2D thin film (2–20 nm), this must be compared with the magnetic length to assess whether 3D orbital physics is relevant. On the other hand, experimentally, the SdH frequencies do not change upon tilting the magnetic field direction, suggesting strong 2D character (perhaps checking $1/\cos\theta$ scaling of the SdH frequency would bolster this). How does one reconcile this?"*

   Answer: We thank the Reviewer #2 for raising this important point and for giving

us the opportunity to clarify a key aspect of our analysis. While the microscopic Hamiltonian we employ is three-dimensional, all transport quantities relevant to the experiments—including the Fermi-surface averages entering the symmetric magnetoresistance coefficient $C(\mu)$—are evaluated for a quasi-two-dimensional Fermi surface. This is precisely what controls the gate-voltage dependence of the magnetoresistance, and it is this quasi-2D treatment that leads to the excellent quantitative agreement between theory and experiment.

The use of a 3D Weyl-type Hamiltonian is motivated by the internal structure of the Bloch states in tellurene. Tellurene consists of stacked helical chains and hosts symmetry-protected Weyl nodes in its bulk band structure; even in thin flakes (2–20 nm), the low-energy Bloch wavefunctions retain their three-dimensional orbital and spin texture. The thin-film geometry primarily quantizes motion perpendicular to the plane but does not eliminate the underlying 3D Hilbert-space geometry that governs the quantum metric and interband couplings. Our Hamiltonian is therefore designed to faithfully capture this internal structure.

At the same time, the transport itself is explicitly treated as quasi-2D. This is consistent with the experimental observation that the Shubnikov–de Haas frequencies are insensitive to magnetic-field tilt, indicating a two-dimensional Fermi surface. Accordingly, in our calculations the momentum integration is performed over a 2D Fermi contour, which directly determines the scaling of $C(\mu)$ with gate voltage. This quasi-2D treatment is now stated explicitly in the revised manuscript.

In summary, there is no inconsistency between the two descriptions: the system exhibits quasi-2D transport while inheriting its quantum-metric properties from an underlying 3D Weyl band structure. This separation between transport dimensionality and Bloch-state geometry is precisely what allows the quantum-geometric mechanism to operate in thin tellurene devices and is borne out by the quantitative agreement with experiment.

6. In-plane magnetic field response – *“The magnetoresistance is not zero when B is parallel to the 2D plane. In the strict 2D limit, orbital Lorentz effects should be suppressed. What remains is spin or spin–orbit physics. In particular, when B is parallel to the internal polarization direction, a small positive MR (3%) is observed. The origin of this contribution should be discussed, especially since the proposed mechanism appears inactive in this configuration.”*

   Answer: Indeed, when $\mathbf{B} \parallel \boldsymbol{\mathcal{E}}$ the intrinsic quantum-geometric mechanism of diffusion identified in our work is forbidden by symmetry, since it scales as $(\boldsymbol{\mathcal{E}} \times \mathbf{B})^2$ it vanishes identically in this configuration. The remaining positive MR is therefore extrinsic and orbital in origin.

   A natural and well-established explanation is the Parish–Littlewood MR, arising from spatial inhomogeneities of carrier density and/or mobility in an otherwise classical conductor. In tellurene devices, such inhomogeneity is expected due to finite thickness combined with top and back gating, imperfect screening, and electrostatic band bending across the film. While each locally homogeneous region obeys standard Drude

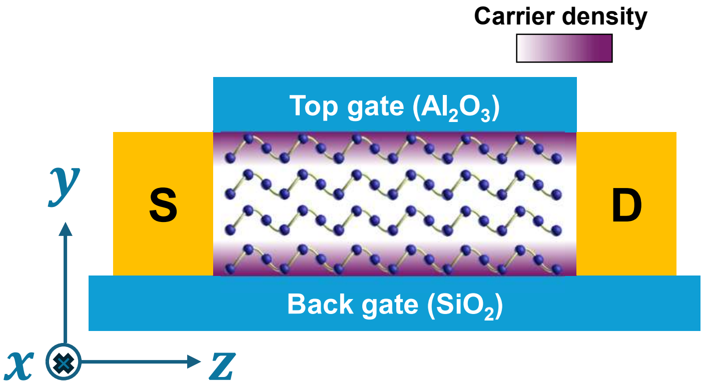

Figure 7. Inhomogeneous charge distribution. Due to the presence of both top and back gates the charge distribution accross the thickness of the tellurene flakes is not homogeneous.

transport and exhibits no longitudinal MR for $\mathbf{B} \perp \mathbf{I}$, spatial variations of the local Hall response force current streamlines to bend, generating additional dissipation at the macroscopic level.

Within effective-medium theory, this mechanism leads to a symmetric positive MR

$$\rho_{zz}(B) \simeq \sqrt{\rho_0^2 + C(\delta\rho_\perp)^2 B^2}, \tag{17}$$

which rapdily crosses over to a linear-in-$|B|$ behavior in the case of large inhomogeneities. This Parish–Littlewood contribution is purely classical, spin independent, and does not rely on band topology or quantum geometry. Its magnitude, symmetry, and $\sin(\theta)$ dependencies are fully consistent with the small positive MR observed experimentally when $\mathbf{B} \parallel \boldsymbol{\mathcal{E}}$. Full details of the effective-medium analysis are provided in the Supplementary Information.

Finally, the linear and symmetric component observed experimentally for rotations in the xy plane: is a Parish–Littlewood MR ($\propto |\sin\theta|$). For the in-plane rotation $\mathbf{B} = B(\sin\theta\,\hat{x} + \cos\theta\,\hat{y})$ (from $\hat{y}$ to $\hat{x}$ and back to $-\hat{y}$), the observed symmetric linear-in-$|B|$ component follows a $\sin\theta$ envelope. This angular dependence is expected for the Parish–Littlewood (effective-medium) magnetoresistance, which is a purely orbital and extrinsic mechanism arising from spatial inhomogeneity of carrier density and/or mobility (see Fig. 7). In this scenario the magnitude of the positive linear MR is controlled by the Lorentz deflection of current streamlines and therefore scales with the component of magnetic field perpendicular to the current $\mathbf{J} \parallel \hat{z}$, namely $B_\perp = |\mathbf{B} \times \hat{z}|$. For the xy rotation one finds $B_\perp = B|\sin\theta|$, and in the Hall-dominated effective-medium regime the longitudinal response takes the robust form $\Delta R_{zz}^{\mathrm{PL}} \propto |B_\perp|$, hence

$$\Delta R_{zz}^{\mathrm{PL}}(\theta, B) \propto |B|\,|\sin\theta|. \tag{18}$$

Importantly, this contribution is symmetric in B (orbital, dissipative) and persists even when the intrinsic quantum-geometric negative MR channel proportional to ($\boldsymbol{\mathcal{E}} \times$

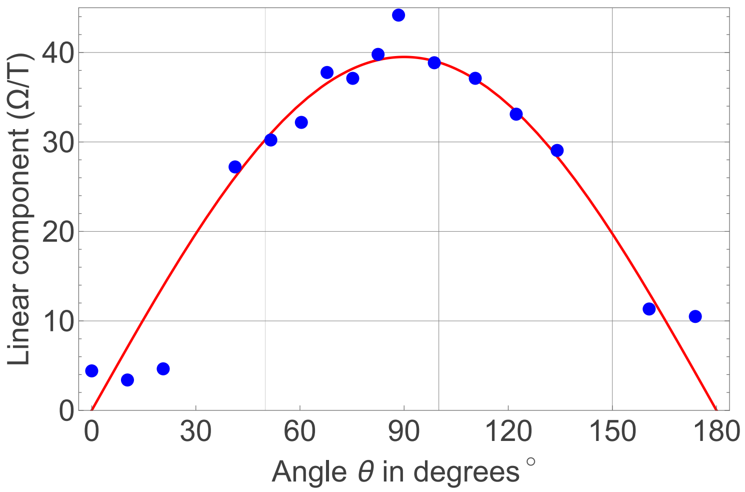

Figure 8. Parish–Littlewood magnetoresistance.

$\mathbf{B})^2$ is symmetry-suppressed in the relevant geometry. Therefore, a $\sin\theta$-modulated linear symmetric MR is most naturally attributed to the Parish–Littlewood mechanism rather than to an intrinsic band-structure effect (see Fig. 8).

RESPONSE TO Reviewer #3

We thank the Reviewer #3 for the careful evaluation of our manuscript.

1. *Why the R_{zz}/R_0 is only dependent with the macroscopic polarization electric field, $\boldsymbol{\mathcal{E}}$? Which may include the applied electric field along the current?*

   Answer: The resistance R_{zz} discussed in this work is a linear-response transport coefficient. By definition, in linear response the applied electric field along the current direction, E_z, enters only through Ohm's law,

   $$j_z = \sigma_{zz} E_z, \tag{19}$$

   and therefore factors out of the resistance $R_{zz} = E_z/j_z$. If the resistance itself depended explicitly on E_z, the transport would be nonlinear. Indeed, quantum-geometric effects are well known to play a prominent role in nonlinear transport; however, the central result of the present work is precisely to demonstrate that quantum geometry can also leave a strong and experimentally observable imprint on linear magnetotransport.

   The electric field $\boldsymbol{\mathcal{E}}$ that appears in our expression for R_{zz}/R_0 is not the externally applied driving field, but the intrinsic crystallographic polarization field associated with the lone-pair polar structure of tellurene. This internal field is a static property of the crystal and exists even in equilibrium, independently of the transport measurement. It therefore legitimately enters the material-dependent prefactor of the linear-response conductivity via the drift–Zeeman coupling and the resulting geometric diffusion. In contrast, the externally applied field along the current direction serves only as the probe of the linear response and does not renormalize the resistance itself.

Response to Reviewer #1

We thank Reviewer #1 for the careful reading of our revised manuscript and for the positive assessment. We are grateful for the two final comments, which helped us further improve the clarity and accuracy of our work. Our point-by-point responses are provided below.

1. *"The authors have addressed all my comments in the response letter. Before fully supporting the publication of the manuscript in Nature Communications, I would like the authors to include their response to my comment #4 (regarding the role of applied gate voltage E_y and the built-in electric polarization E_x) into supplementary."*

   Response: We thank the Reviewer for this suggestion. We agree that this clarification belongs in the Supplementary Information rather than only in the response letter.

   Accordingly, we have now incorporated a dedicated discussion into the Supplementary Information (Sec. 4.5.) regarding the distinction between the built-in inversion-breaking polar field and the gate-induced out-of-plane displacement field.

2. *"Besides, I also notice a minor caveat in the introduction part, 'Most remarkably, in Weyl semimetals (e.g., WTe_2 films), NMR reaches -60% at 60 T under pulsed fields, driven by the chiral anomaly, in the ultraquantum limit [13].' I thought Ref. 13 mainly reported colossal positive MR in ultraclean WTe_2 single crystal. Can the authors check again this statement and citation?"*

   Response: The Reviewer is absolutely correct: Ref. 13 [Ali *et al.*, Nature **514**, 205–208 (2014)] reports extremely large, non-saturating *positive* magnetoresistance in bulk WTe_2, rather than negative magnetoresistance. We have corrected the Introduction and removed the incorrect attribution of giant NMR/chiral-anomaly physics to that specific reference.

   To preserve the intended progression of the Introduction, moving from weaker to stronger NMR responses reported in the literature, we have replaced that example with a more appropriate one: the giant negative magnetoresistance observed in individual Cd_3As_2 nanowires, where NMR reaches approximately -63% and is attributed to the chiral anomaly. The Introduction and bibliography have been revised accordingly.

Response to Reviewer #2

We thank Reviewer #2 for the thoughtful and constructive report. We appreciate the Reviewer's positive assessment of the central idea and experimental results, and we are grateful for the two remaining criticisms, which helped us place the theoretical framework on a substantially more systematic footing. In the revised manuscript and Supplementary Information we have addressed both points in depth, as detailed below.

1. *"Derivation of the drift–Zeeman term. While the revised derivation removes the explicit gauge inconsistency, it still relies on treating the internal polarization as a uni-*

form scalar potential $-e\,\mathbf{E}\cdot\mathbf{r}$. In my opinion, this is not the correct starting point... In a crystal, inversion breaking enters through the periodic potential and generates spin–orbit coupling via $(\nabla V\times p)\cdot\sigma$. A systematic multiband Bloch or $k\cdot p$ treatment, followed by projection, would in general produce all symmetry-allowed B-dependent terms at the same order..."

Response: We thank the Reviewer for this important comment. We agree that the correct microscopic starting point is the multiband crystal Hamiltonian, in which inversion breaking is encoded in the inversion-odd part of the periodic crystal potential, rather than in a uniform scalar potential $-e\,\mathbf{E}\cdot\mathbf{r}$.

Following the Reviewer's suggestion, we have completely reformulated the derivation in the Supplementary Information using this proper microscopic starting point. The revised treatment has significantly strengthened our findings and is summarized as follows:

We begin from a multiband $k{\cdot}p$ Hamiltonian that contains the periodic crystal potential $V(\mathbf{r}) = V_{\text{even}}(\mathbf{r}) + V_{\text{odd}}(\mathbf{r})$. We then perform a systematic Löwdin (Schrieffer–Wolff) downfolding onto the low-energy conduction-band Kramers doublet. The perturbations retained in the projection are: (i) the $k{\cdot}p$ term, H_{kp}; (ii) the scalar inversion-odd term, H_{Π}; (iii) the inversion-odd spin-orbit interaction term, H_{inv}; (iv) the B-linear spin-orbit term, H_B^{so}, generated by minimal substitution; and (v) the B-linear orbital perturbation, H_B^{orb}. This approach yields, in a single gauge-consistent multiband framework, the complete set of leading projected terms relevant to our low-energy theory.

The resulting bulk effective Hamiltonian takes the tensor structure

$$H_{\text{eff}}^{(3D)}(\mathbf{k}) = \varepsilon_0(\mathbf{k})\,\mathbb{I}_2 + \beta_{ij}k_i\sigma_j + \gamma_{kij}\Pi_iB_j\sigma_k + \kappa_{ijm}\Pi_iB_jk_m\,\mathbb{I}_2 + \zeta_{kijm}\Pi_iB_jk_m\sigma_k + \chi_{ij}\Pi_iB_j\,\mathbb{I}_2 + \dots . \quad (1)$$

where Π_i represents the components of the projected polar strength due to the inversion-odd crystal potential. The tensors are defined as follows: β_{ij} is the zero-field Weyl tensor, γ_{kij} is the generalized Drift–Zeeman tensor, κ_{ijm} is the generalized Drift–Orbit tensor, ζ_{kijm} describes field-induced renormalization of the Weyl spin texture, and χ_{ij} is a scalar, field-dependent band-origin shift. The dots denote higher-order terms. In agreement with the Reviewer's observation, thus systematic derivation indeed generates *all* symmetry-allowed leading B-dependent terms at the same order, rather than isolating a specific one by construction.

In particular, the spin-dependent Drift–Zeeman sector is no longer attributed to a single microscopic channel. In the revised derivation, the tensor γ_{kij} is shown to receive contributions from three distinct mixed projections,

$$\gamma_{kij} = \gamma_{kij}^{(\text{inv},so)} + \gamma_{kij}^{(\Pi,so)} + \gamma_{kij}^{(\text{inv},orb-p)},$$

origination from the channels $H_{\text{inv}}\times H_B^{\text{so}}$, $H_{\Pi}\times H_B^{\text{so}}$, and $H_{\text{inv}}\times H_{B,p}^{\text{orb}}$. Similarly, the Drift–Orbit tensor κ_{ijm} emerges from the scalar channel $H_{\Pi}\times H_{B,k}^{\text{orb}}$. In this way, the

revised Supplementary Information makes explicit that the Drift–Zeeman term is a physical, gauge-invariant interaction, and is part of a broader multiband projected structure that also contains other terms of the same formal order.

After symmetry reduction near the H point of bulk tellurium, the projected tensors reduce to the following leading forms:

**Tensor**	**Symmetry-reduced form**	**Meaning**
β_{ij}	$\lambda_\perp(k_x\sigma_x + k_y\sigma_y) + \lambda_z k_z\sigma_z$	Zero-field anisotropic Weyl spin texture
γ_{kij}	$\gamma_\perp \Pi_x B_z\sigma_y - \gamma_z\Pi_x B_y\sigma_z = -\gamma\cdot(\Pi\times\mathbf{B})\cdot\sigma$	Drift–Zeeman splitting
κ_{ijm}	$\kappa(\mathbf{\Pi}\times\mathbf{B})\cdot\mathbf{k}\,\mathbb{I}_2$	Drift–Orbit scalar shift
ζ_{kijm}	$\zeta_\perp[(\Pi\times B)_x k_x\sigma_x + (\Pi\times B)_y k_y\sigma_y]$ $+\,\zeta_z(\Pi\times B)_z k_z\sigma_z$	Field-induced renormalization of Weyl SOC
χ_{ij}	Scalar $\Pi_i B_j\,\mathbb{I}_2$ shift	Band-origin shift (not retained in the minimal model)

The main text has been updated to reflect these improvements. We now explicitly state the resulting projected low-energy Hamiltonian and its leading terms, while referring the reader to the Supplementary Information for the full multiband derivation. In particular, we clarify that the effective internal field $\boldsymbol{\mathcal{E}}$ now serves only as a compact low-energy parameterization of the projected inversion-breaking polar strength already encoded in the crystal potential and in the downfolded Bloch Hamiltonian. We are confident that this revised treatment places the microscopic origin of the proposed mechanism on the systematic footing requested by the Reviewer.

2. *"Dimensionality and effective Hamiltonian. The authors have commented, 'the system exhibits quasi-2D transport while inheriting its quantum-metric properties from an underlying 3D Weyl band structure.' This does not make sense to me... In this regime, motion along the confinement direction should be quantized, and the appropriate low-energy description should be a reduced Hamiltonian* $H_{\text{eff}}(k_x, k_z)$*..."*

   Response: We appreciate the Reviewer raising this important issue. We fully agree that the quasi-2D character of the transport regime warranted implementation within our theoretical framework. In the revised manuscript and Supplementary Information, we now clearly distinguish between: (i) the bulk 3D crystal Hamiltonian, which serves as the microscopic parent theory from which the symmetry-allowed low-energy operators are derived, and (ii) the reduced quasi-2D Hamiltonian, which provides the appropriate transport theory for the confined device geometry.

   As noted in our response to the first query of Reviewer #2, the multiband downfolding in the revised Supplementary Information is performed at the level of the bulk crystal Hamiltonian. This systematic procedure yields the general projected 3D low-energy structure presented in Eq. (1).

We then explicitly reduce this bulk parent Hamiltonian to the quasi-2D transport regime relevant to the experiment. Motivated by the electron-side Shubnikov–de Haas data, which are consistent with a single occupied confinement subband and a 2D Fermi surface, we introduce a confinement potential along $\hat{y}$ and factorize the wavefunction as

$$\Psi(\mathbf{r}) = \psi(x, z)\phi_1(y),$$

where $\phi_1(y)$ is the lowest confinement subband envelope. The effective transport Hamiltonian is obtained by projecting the 3D model onto this subband,

$$H_{\text{eff}}^{(2\text{D})} = \langle \phi_1 | H_{\text{eff}}^{(3\text{D})} | \phi_1 \rangle_y .$$

In this reduction, all terms linear in k_y vanish because $\langle \phi_1 | -i\partial_y | \phi_1 \rangle = 0$, whereas quadratic terms in k_y manifest only as a constant confinement energy shift. Consequently, the resulting transport Hamiltonian is not obtained by arbitrarily setting $k_y = 0$; rather, it arises naturally from a controlled, systematic subband projection.

The resulting quasi-2D Hamiltonian depends only on (k_x, k_z) and contains the same symmetry-allowed operator structures as the bulk parent theory, now evaluated within the confined subspace. Specifically, the two leading magnetoelectric terms retained in the main text remain

$$H_{DZ} \propto (\boldsymbol{\mathcal{E}} \times \mathbf{B}) \cdot \boldsymbol{\sigma}, \qquad H_{DO} \propto (\boldsymbol{\mathcal{E}} \times \mathbf{B}) \cdot \mathbf{k}\, \mathbb{I}_2,$$

where the effective coefficients are renormalized by the confinement. This approach ensures that the 3D microscopic derivation and the quasi-2D transport theory are fully consistent with one another.

We have also revised the main text to ensure this logical progression is fully transparent. We now first present the multiband downfolding and the resulting 3D projected Hamiltonian, followed by the confinement reduction to the quasi-2D Hamiltonian used for the transport and quantum-metric analysis. Consequently, all subsequent band-splitting formulas and quantum-metric expressions are now formulated directly in the reduced (k_x, k_z) subspace, appropriate to describe the experimental results.

We believe these revisions resolve the dimensionality concerns raised by the Reviewer. The bulk 3D Hamiltonian is the microscopic parent theory required by symmetry and multiband projection, whereas the actual transport model analyzed in the paper is a controlled, dimensionally reduced quasi-2D Hamiltonian in full accord with the experimental evidence.

Response to Reviewer #3

We thank Reviewer #3 for the careful reading of our revised manuscript and for the positive assessment. We are grateful for the Reviewer's support and recommendation for publication in *Nature Communications*.
